# Association of *PTPRT* Mutations with Cancer Metastasis in Multiple Cancer Types

**DOI:** 10.1155/2022/9386477

**Published:** 2022-06-25

**Authors:** Chao Chen, Haozhen Liu, Qumiao Xu, Xiuqing Zhang, Feng Mu, Jixian Liu

**Affiliations:** ^1^Department of Thoracic Surgery, Peking University Shenzhen Hospital, Shenzhen Peking University-The Hong Kong University of Science and Technology Medical Center, Shenzhen 518035, China; ^2^BGI-Shenzhen, Shenzhen 518083, China; ^3^University of Chinese Academy of Sciences, Shenzhen 518083, China; ^4^BGI, Shenzhen 518083, China

## Abstract

Metastasis is one of the characteristics of advanced cancer and the primary cause of cancer-related deaths from cancer, but the mechanism underlying metastasis is unclear, and there is a lack of metastasis markers. *PTPRT* is a protein-coding gene involved in both signal transduction and cellular adhesion. It is also known as a tumor suppressor gene that inhibits cell malignant proliferation by inhibiting the STAT3 pathway. Recent studies have reported that *PTPRT* is involved in the early metastatic seeding of colorectal cancer; however, the correlation between *PTPRT* and metastasis in other types of cancer has not been revealed. A combined analysis using a dataset from the genomics evidence neoplasia information exchange (GENIE) and cBioPortal revealed that *PTPRT* mutation is associated with poor prognosis in pan-cancer and non-small-cell lung cancer. The mutations of *PTPRT* or “gene modules” containing *PTPRT* are significantly enriched in patients with metastatic cancer in multiple cancers, suggesting that the *PTPRT* mutations serve as potential biomarkers of cancer metastasis.

## 1. Introduction

Metastasis is the main cause of death in patients with cancer; however, the mechanisms and molecular markers of this process are yet uncharacterized [1, 2]. Receptor-type tyrosine-protein phosphatase T is an enzyme encoded by the *PTPRT* gene. It is a well-known tumor suppressor gene that is frequently mutated in several cancers [3]. The gene may be involved in both signal transduction and cellular adhesion and is also known to inhibit malignant cell proliferation by inhibiting the STAT3 pathway [4–7]. Recent studies have reported that *PTPRT* mutations may be associated with the tumor mutation burden (TMB) and could provide clinically predictive implications for immune checkpoint inhibitor (ICI) therapies [8, 9]. Hu et al. reported that *PTPRT* may be involved in the early metastatic seeding of colorectal cancer [10]. However, to the best of our knowledge, the association between *PTPRT* and cancer metastasis has not been investigated through a comprehensive analysis of large clinical datasets.

To address this question, we retrospectively analyzed the somatic mutations and cancer prognostic status from the previously published data [7–9]. The integration of somatic mutations and clinical prognostic information from multiple cohorts retrieved 16,182 metastatic and/or stage IV cancers and 26,480 early primary cancers. Subsequently, we found that *PTPRT* mutation was significantly associated with cancer metastasis in 6 common cancers, including breast cancer (BRCA), colorectal cancer (CRC), esophageal gastric cancer (EGC), non-small-cell lung cancer (NSCLC), skin cutaneous melanoma (SKCM), and skin cutaneous nonmelanoma (SKCNM). Furthermore, *PTPRT* mutation is associated with poor progression-free survival in pan-cancer and NSCLC. These results confirmed the effect of *PTPRT* mutation on tumor development and progression.

## 2. Materials and Methods

### 2.1. Genomic Data

All cancer samples and somatic mutation data were downloaded from cBioPortal (https://www.cbioportal.org) and GENIE databases (v6.1, http://synapse.org/genie) [11]. All nonsilent mutations, including missense, frameshift, nonsense, nonstop, splice site, and translation start site mutations, were considered. To ensure the consistency of data sources for finding potential metastasis markers, we screened the samples in the cBioPortal as follows: (1) the samples were sequenced on an MSK-impact panel; (2) the samples should be identified for whether they are primary tumors or metastasis tissues and the tumor stage; (3) because the mutation characteristics of MSI-H samples are different, we excluded the samples known to be MSI-H [12, 13]. The samples of unknown tissue origins in the GENIE database were excluded. Next, we obtained 16,182 metastatic and/or stage IV cancers (hereafter denoted as “metastatic cancer”) and 26,480 early primary cancers (primary cancers with stages I–III). To remove noise from the analysis, *PTPRT* or other driver genes with nonsilent mutations that occurred in at least five metastatic cancers were selected, resulting in 6 types of cancers with 10,068 metastatic cancer samples and 13,487 early primary cancer samples, respectively. These 23,555 samples were used for further analysis (Table 1).

### 2.2. Gene Module Mutation Enrichment Analysis

**Figure 1:**
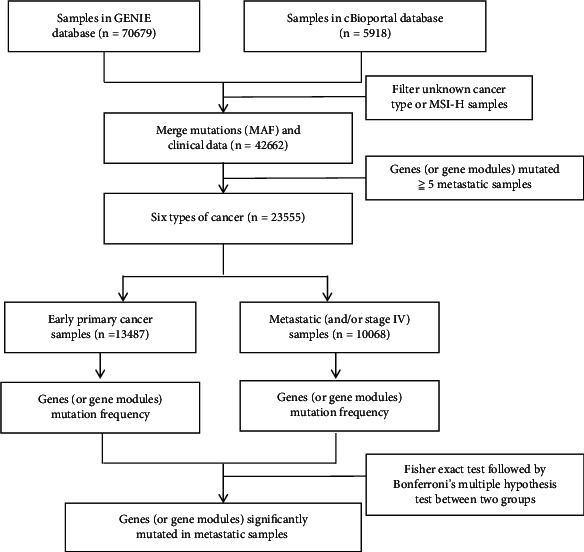
The pipeline for gene module mutation enrichment analysis. Samples in GENIE (v6.1) were downloaded from https://www.synapse.org/genie. Samples in cBioPortal were downloaded from https://www.cbioportal.org. Early primary cancer: primary cancer samples of stages I–III; metastatic samples: primary cancer samples of stage IV or metastasis cancers.

The enrichment analysis pipeline is shown in Figure 1. After collecting the genomic mutation data from the database, we searched for the biomarkers of metastatic cancer by comparing the mutation frequency difference of a single driver gene (or “gene modules”) between early primary cancer samples (*n* = 13,487) and metastatic cancer samples (*n* = 10,068). A gene module is defined as a combination of two or more driver genes mutated in the same sample. The driver genes of each cancer type predicted by Matthew et al. were selected for candidate gene models [14]. The differential analysis used the two-sided Fisher's exact test, followed by Bonferroni's multiple hypothesis tests.

### 2.3. The Neoantigen Prediction for Recurrent Mutations in *PTPRT*

The human leukocyte antigen (HLA) alleles of patients were downloaded from TCIA database [15], the mutations of patients in TCGA pan-cancer cohort (*N* = 10967) were downloaded from the cBioPortal database [16], and the recurrent mutations (frequency ≥ 2) of PTPRT were selected for neoantigen prediction using NetMHC [17], NetMHCpan [18], PickPocket [19], PSSMHCpan [20], and SMM [21]. The peptides with a length of 8–11 mers and an affinity (IC50) < 500 nM in at least two tools were regarded neoantigens.

### 2.4. Statistical Analysis

Statistical analyses were carried out using R studio (R 4.0.2), and the differential significance of mutation frequency between primary cancer and metastatic samples was determined by Fisher's exact test. The *P* value was adjusted to *q* value by Bonferroni's multiple hypothesis tests. For survival analysis, we used survival (v3.1-12) and survminer (v0.4.9), and the difference in survival was analyzed using the log-rank test, and the survival data were downloaded from cBioPortal. The different gene expressions of *PTPRT* were analyzed in tumor and normal samples using online tools (https://www.proteinatlas.org and https://cistrome.shinyapps.io/timer), and the significance of differential expression was evaluated using the Wilcoxon test (^∗^*P* < 0.05; ^∗∗^*P* < 0.01; ^∗∗∗^*P* < 0.001).

## 3. Results

### 3.1. *PTPRT* Is Downregulated in Multiple Cancer Types

**Figure 2:**
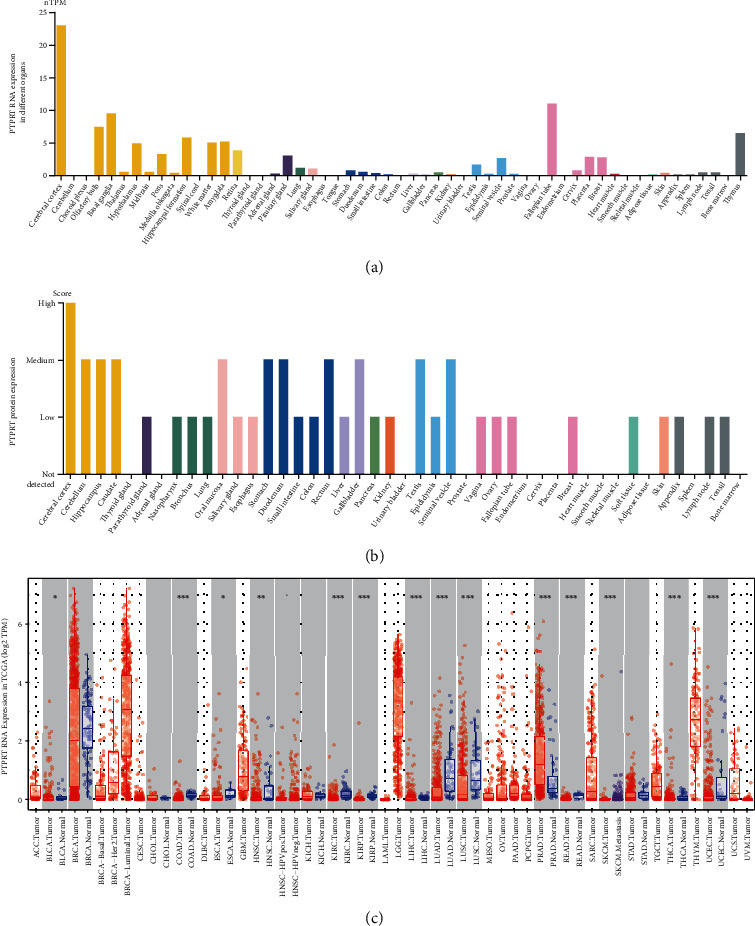
Expression level of *PTPRT* across different organs and in TCGA database. (a) *PTPRT* RNA expression level in different organs and colors refer to the various origins of tissue types. (b) PTPRT protein level in different organs. (c) PTPRT level in tumor and normal samples in TCGA cohort. A total of 17 cancer types with paired expression data; *PTPRT* was significantly downregulated in 12/17 cancer types (^∗^*P* < 0.05; ^∗∗^*P* < 0.01; ^∗∗∗^*P* < 0.001).

Since *PTPRT* is a tumor suppressor in cancer, we elucidated the expression landscape of *PTPRT* in tumorigenesis. PTPRT is mainly expressed in the brain tissues, and that in the other tissues is lower (Figure 2(a)). However, the protein level of PTPRT was medium in multiple organs (Figure 2(b)). The analysis of the expression data of samples in TCGA database revealed that *PTPRT* is downregulated in tumors compared to the paired normal samples. As shown in Figure 2(c), a total of 17 cancer types had significantly downregulated *PTPRT* levels in cancer tissues compared to normal tissue in most cancer types (the expression of *PTPRT* in 12/17 cancer types was downregulated, Figure 2(c)). In addition, some studies reported that the downregulation of *PTPRT* expression is associated with poor prognosis [22, 23].

### 3.2. The Mutation Landscape of *PTPRT* across Different Cancer Types

**Figure 3:**
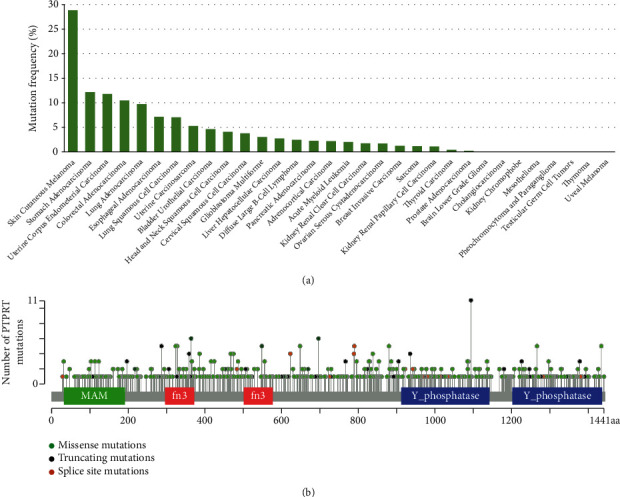
Mutation spectrum of *PTPRT* in cancers. (a) *PTPRT* mutation across different types of cancers. (b) The lollipop plot shows the protein domain and location of mutations in *PTPRT*. The color of the circles indicates the corresponding mutation type.

*PTPRT* is mutated in various cancers, such as melanoma and gastric cancer. To comprehensively depict the mutation landscape of *PTPRT* in different cancers, the mutation datasets from TCGA pan-cancer cohort (10967 samples), containing 32 cancer types, were collected. *PTPRT* mutations were detected in 24 cancer types, including SKCM, gastric adenocarcinoma, uterine corpus endometrial carcinoma, colorectal adenocarcinoma, and lung adenocarcinoma (Figure 3(a)).

Next, we analyzed the distribution of mutations in *PTPRT*. The lollipop plot showed that the missense and truncating mutations (nonsense, nonstop, frameshift deletion, and frameshift) were randomly distributed in the various functional regions of the gene (Figure 3(b)) without hotspot mutations.

### 3.3. Mutation Enrichment of *PTPRT* and the Associated Gene Modules in Metastatic Cancers

**Figure 4:**
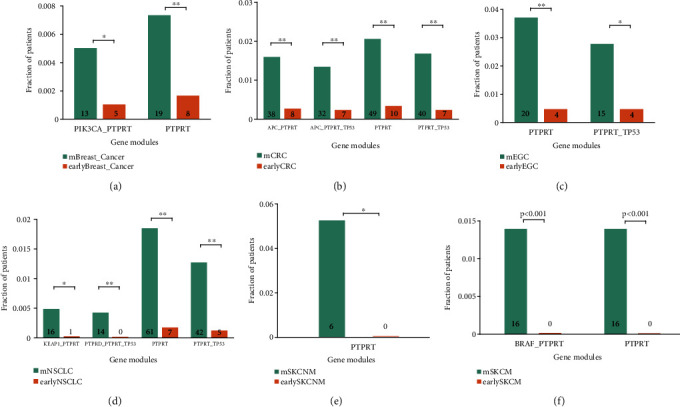
Comparison of mutations in different gene modules between early and metastatic cancers. (a) Breast cancer (BRCA). (b) Colorectal cancer (CRC). (c) Esophagogastric cancer (EGC). (d) Non-small-cell lung cancer (NSCLC). (e) Skin cancer, nonmelanoma (SKCNM). (f) Melanoma (SKCM). Fisher exact test followed by Bonferroni's multiple hypothesis tests, ^∗^*P* < 0.05; ^∗∗^*q* < 0.01. mbreast_cancer, “m” means metastatic and/or stage IV cancers; earlybreast_cancer, “early” means stage I-III primary cancers.

As mentioned in Figure 1, we obtained the mutation data of *PTPRT* and other gene modules in early primary and metastatic tumors from 6 cancer types. Among these, *PTPRT* mutations were significantly mutated in metastatic cancers (Figures 4(a)–4(f)). In melanoma, the *q* value is >0.05, which could be attributed to the small number of primary cancer samples (Figure 4(f), 14/1148 *vs*. 0/669, *q* = 0.156, *P* = 0.00097).

Additionally, many gene modules involved in *PTPRT* were significantly enriched in metastatic cancers. In breast cancer, the combined mutation frequency of *PTPRT* and *PIK3CA* in metastatic breast cancer was significantly higher than in primary cancer (Figure 4(a); *q* = 0.025). In colorectal cancer, the combined mutation frequency of *APC-PTPRT*, *APC-PTPRT-TP53*, and *PTPRT-TP53* was significantly higher in metastatic colorectal cancer than in primary cancer (Figure 4(b); *q* = 3.4*E* − 05, *q* = 0.0006, and *q* = 2.6*E* − 06, respectively). In esophagogastric cancer, the combination mutation frequency of *PTPRT* and *TP53* in metastatic cancer is significantly higher than that in primary cancer (Figure 4(c), *q* = 0.023). The combined alteration of *KEAP1-PTPRT*, *PTPRD-PTPRT-TP53*, and *PTPRT-TP53* was significantly higher in metastatic NSCLC than in primary cancer (Figure 4(d), *q* = 0.02, *q* = 0.0097, and *q* = 1.68*E* − 07, respectively).

Conversely, the mutation frequency of other cancer driver genes or gene modules (such as *TP53*, *PIK3CA*, *ARID1A*, and *BRAF*) was not significantly different between the two groups (*q* > 0.05) or had a slightly higher mutation frequency in primary cancer than in metastatic cancer samples (Supplementary Table S1–6). This demonstrated the specificity of *PTPRT* mutation as a candidate biomarker for cancer metastasis across multiple cancer types.

### 3.4. The Association of *PTPRT* Mutation and the Prognosis of Cancer

**Figure 5:**
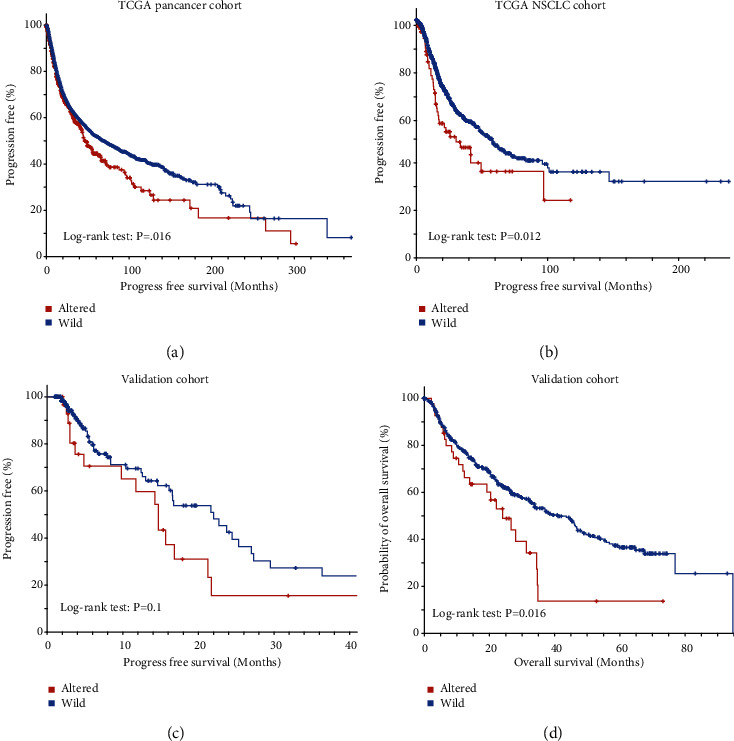
Association of *PTPRT* mutation with cancer prognosis. (a) Correlation between *PTPRT* mutation and progression-free survival in pan-cancer cohort (TCGA pan-cancer atlas, *n* = 10967). (b) Correlation between *PTPRT* mutation and progression-free survival in NSCLC (TCGA NSCLC, *n* = 1053). The red line represents *PTPRT*-altered group, and the blue line represents the *PTPRT* wild-type group. (c, d) Correlation between *PTPRT* mutation with (c) progression-free and (d) overall survival in an independent validation pan-cancer cohort (log-rank test, *P* = 0.1 and *P* = 0.016, respectively).

Next, we explored the effect of *PTPRT* mutation on tumor prognosis. In TCGA pan-cancer cohort (*n* = 10967), the *PTPRT* mutations were associated with poor prognosis of cancers (log-rank test, *P* = 0.016; Figure 5(a)). Similarly, in TCGA NSCLC cohort (TCGA LUAD and LSCC, *n* = 1053), the *PTPRT* mutations were associated with poor progression-free survival in NSCLC (log-rank test, *P* = 0.012; Figure 5(b)). We further analyzed *PTPRT* mutation in another combined pan-cancer cohort conducted by ICGC/TCGA and MSK (validation cohort, *n* = 3418) with similar observations that *PTPRT*-altered groups tend to have poor progression-free survival and overall survival (log-rank test, *P* = 0.1 and *P* = 0.016, respectively, Figures 5(c) and 5(d)).

### 3.5. The Neoantigens Derived from Recurrent *PTPRT* Mutations as Potential Drug Targets

Tumor suppressor genes are difficult to target by conventional drug modalities and are commonly regarded as “undruggable.” Deniger et al. found that some neoantigens derived from hotspot mutations in *TP53* (p.Y220C and p.G245S) had strong immunogenicity, and the transfer of *TP53* “hotspot” mutation-reactive T cell receptors into peripheral blood T cells could be evaluated as a potential therapy for various cancer types [24, 25]. Similarly, we investigated the potential neoantigens from the recurrent mutations of *PTPRT.* Some recurrent mutations (p.G826R and p.R1117C) of *PTPRT* were predicted to generate high-affinity neoantigens in pan-cancer that could be used as potential targets for immunotherapies in the future (Table 2).

## 4. Discussions and Conclusion

**Figure 6:**
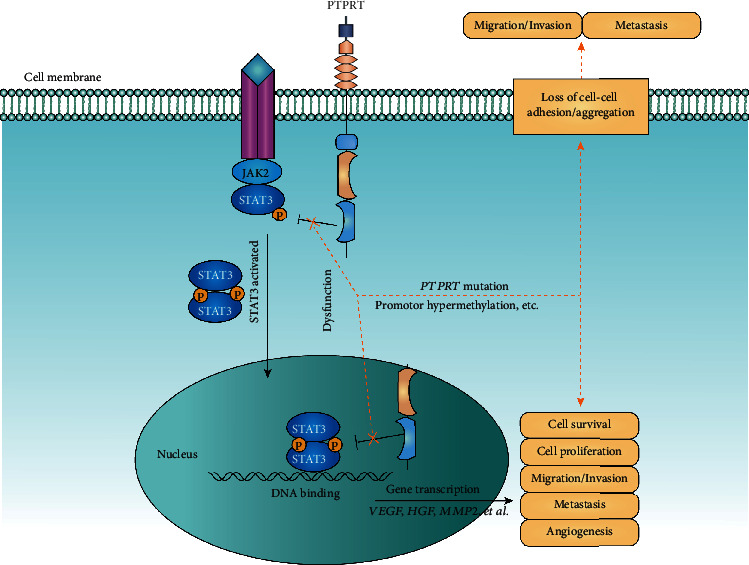
The potential mechanism of *PTPRT* underlying cancer metastasis. *PTPRT* mutations or hypermethylation of the promoter region leads to gene dysfunction, which in turn affects adhesion and aggregation between cells and leads to tumor invasion/migration and metastasis. On the other hand, the dysfunction of PTPRT activates the STAT3 pathway, which promotes the gene transcription and leads to cell survival, proliferation, migration, and metastasis.

Previous studies have demonstrated the functional impact of *PTPRT* mutation in tumor progression and metastasis [6, 26–28]. Wang et al. first identified and confirmed that PTPRT functions as a tumor suppressor [29]. *PTPRT* mutations are often loss-of-function mutations that disrupt cell-cell adhesion, leading to tumor progression and metastasis [30]. Some studies showed that *PTPRT* regulates the STAT3 signaling pathway by dephosphorylation of pSTAT3 [7, 31, 32], thus promoting cell survival, proliferation, angiogenesis, migration, invasion, and metastasis [31, 32]. In the present study, the comprehensive analysis of the mutation data from GENIE and cBioPortal databases revealed that *PTPRT* mutations are significantly enriched in metastatic samples across multiple cancer types, deeming the mutations as risk factors for cancer metastasis in various cancers. In addition to mutations, another factor that affects the function of *PTPRT* is hypermethylation of the promoter region [31], which also leads to the dysfunction of *PTPRT*. Based on the previous results and our analysis, we propose the potential mechanism of *PTPRT* dysfunction leading to tumor metastasis in Figure 6.

Since *PTPRT* acts as a tumor suppressor in cancer, direct targeting of *PTPRT* for cancer therapy has not been reported. Conversely, STAT3 represents a promising therapeutic target in clinical trials [32]. Peyser et al. reported that patients with frequent promoter hypermethylation or mutations of *PTPRT* are sensitive to STAT3 inhibitors [7, 31]. Thus, *PTPRT* functional status might have implications for the efficacy of therapies targeting STAT3.

Several studies suggested that *PTPRT* mutation is positively correlated with TMB, and patients with *PTPRT* mutation might benefit from ICI therapy, indicating that *PTPRT* is a potential biomarker in immunotherapy [8, 9]. Based on the prediction based on recurrent mutations in *PTPRT*, we identified potential *PTPRT* neoantigens (Table 2) that might have a therapeutic value in immunotherapies, for example, cancer vaccines or T cell therapies.

In conclusion, the integration of mutation data and clinical information from multiple cohorts suggested that *PTPRT* mutations have a significant influence on cancer prognosis and may serve as potential biomarkers for cancer metastasis. This finding emphasizes on *PTPRT* as a specific therapeutic target for advanced cancers.

## Figures and Tables

**Table 1:** Distribution of cancer samples.

Cancer type	Metastatic and/or stage IV (#)	Early primary cancer (#)
Breast cancer (BRCA)	2587	4814
Colorectal cancer (CRC)	2382	2992
Esophagogastric cancer (EGC)	539	849
Non-small-cell lung cancer (NSCLC)	3298	4012
Skin cancer, melanoma (SKCM)	1148	669
Skin cancer, nonmelanoma (SKCNM)	114	151

**Table 2:** The neoantigens derived from recurrent *PTPRT* mutations in pan-cancer.

Chr	Location	AA-change	Peptide	Frequency	HLA types
chr20	40790168	G826R	RELSQPTLTI	5	HLA-B44:02
chr20	40735467	R1117C	GVVDIFNCVC	4	HLA-A02:06
chr20	41385177	V262I	ADTAQRSISK	4	HLA-A11:01
chr20	40727077	E1280V	VMLNVMDTA	3	HLA-A02:05; HLA-B35:01; HLA-B42:01; HLA-C07:01; HLA-C08:02
chr20	41419951	V124M	RSSPGALNVYM	3	HLA-A30:02
chr20	41420025	D99G	LLLPTLKENGT	2	HLA-A02:01
chr20	40747103	M977I	TVKDFWRIIW	2	HLA-B57:01
chr20	41101110	R416C	EPFGYAVTCCH	2	HLA-B07:02; HLA-A02:01
chr20	40864873	Y780H	YSYSYHLKLA	2	HLA-A30:01; HLA-C06:02; HLA-C03:03
chr20	40911144	R721C	GETKINCVC	2	HLA-B40:01
chr20	41306583	R359Q	YEIQVLLTR	2	HLA-B40:01
chr20	40980816	Y557C	HLFVGLCPGT	2	HLA-A02:01
chr20	40877418	V741M	KQMDNTVKMA	2	HLA-A02:06; HLA-B40:03; HLA-C03:04
chr20	40713337	T1393I	REGRIVVHCL	2	HLA-B40:01
chr20	40713431	Y1343N	WPANRDTPP	2	HLA-B55:01
chr20	40730915	R1188H	TLNIVTPHV	2	HLA-A02:01

## Data Availability

The data reported in this study are available in the supplementary materials, and other data can be obtained by contacting the corresponding author. The code used for this manuscript is available at https://github.com/gkdsuperchan/PTPRT-analysis.

## Supplementary Materials

Supplementary Materials Supplementary Table S1: differences in the mutational frequency between genes (or gene modules) in early and metastatic breast cancer (BRCA). Supplementary Table S2: differences in the mutational frequency between genes (or gene modules) in early and metastatic colorectal cancer (CRC). Supplementary Table S3: differences in the mutational frequency between genes (or gene modules) in early and metastatic esophagogastric cancer (EGC). Supplementary Table S4: differences in the mutational frequency between genes (or gene modules) in early and metastatic non-small-cell lung cancer (NSCLC). Supplementary Table S5: differences in the mutational frequency between genes (or gene modules) in early and metastatic skin cancer nonmelanoma (SKCNM). Supplementary Table S6: differences in the mutational frequency between genes (or gene modules) in early and metastatic skin cancer nonmelanoma (SKCM).
