# Supplementary material for: Association of PTPRT Mutations with Cancer Metastasis in Multiple Cancer Types
Source: Biomed Res Int. 2022 Jun 25;2022:9386477. doi: 10.1155/2022/9386477 (PMC9250438; doi:10.1155/2022/9386477)
Supplement: Supplementary Materials — Supplementary Table S1: differences in the mutational frequency between genes (or gene modules) in early and metastatic breast cancer (BRCA). Supplementary Table S2: differences in the mutational frequency between genes (or gene modules) in early and metastatic colorectal cancer (CRC). Supplementary Table S3: differences in the mutational frequency between genes (or gene modules) in early and metastatic esophagogastric cancer (EGC). Supplementary Table S4: differences in the mutational frequency between genes (or gene modules) in early and metastatic non-small-cell lung cancer (NSCLC). Supplementary Table S5: differences in the mutational frequency between genes (or gene modules) in early and metastatic skin cancer nonmelanoma (SKCNM). Supplementary Table S6: differences in the mutational frequency between genes (or gene modules) in early and metastatic skin cancer nonmelanoma (SKCM). [file 9386477.f1.docx]

# Association of *PTPRT* Mutations with Cancer Metastasis in Multiple Cancer Types

Chao Chen^1, 2, *^, Haozhen Liu^1^, Qumiao Xu^2,3^, Xiuqing Zhang^2,3^, Feng Mu^4, *^, Jixian Liu^1, *^

^1^ Department of Thoracic Surgery, Peking University Shenzhen Hospital, Shenzhen Peking University-The Hong Kong University of Science and Technology Medical Center, Shenzhen 518035, China.

^2^ BGI-Shenzhen, Shenzhen 518083, China.

^3^ University of Chinese Academy of Sciences, Shenzhen, 518083, China.

^4^ BGI, Shenzhen 518083, China.

^*^Correspondence should be addressed to Chao Chen (gkd.chaochen@foxmail.com), Feng Mu (mufeng@mgi-tech.com), and Jixian Liu (252110465@qq.com).

**Supplementary Materials**

**Supplementary Table S1.**

Differences in the mutational frequency between genes (or gene modules) in early and metastatic Breast Cancer (BRCA).

| Gene_Module | #mut_mBreast_Cancer | #wild_mBreast_Cancer | mRatio | #mut_earlyBreast_Cancer | #wild_earlyBreast_Cancer | early_Ratio | pvalue | qvalue |
| --- | --- | --- | --- | --- | --- | --- | --- | --- |
| PIK3CA | 872 | 1715 | 0.33707 | 1774 | 3040 | 0.368508517 | 0.007569 | 0.105967 |
| PIK3CA_PTPN11 | 6 | 2581 | 0.002319 | 3 | 4811 | 0.000623182 | 0.07394 | 1 |
| PIK3CA_PTPRD | 11 | 2576 | 0.004252 | 31 | 4783 | 0.006439551 | 0.25945 | 1 |
| PIK3CA_PTPRD_TP53 | 7 | 2580 | 0.002706 | 8 | 4806 | 0.00166182 | 0.41713 | 1 |
| PIK3CA_PTPRT | 13 | 2574 | 0.005025 | 5 | 4809 | 0.001038637 | 0.00183 | 0.025623 |
| PIK3CA_PTPRT_TP53 | 8 | 2579 | 0.003092 | 3 | 4811 | 0.000623182 | 0.020865 | 0.292112 |
| PIK3CA_TP53 | 303 | 2284 | 0.117124 | 435 | 4379 | 0.090361446 | 0.000291 | 0.004068 |
| PTPN11 | 14 | 2573 | 0.005412 | 7 | 4807 | 0.001454092 | 0.004465 | 0.062516 |
| PTPN11_TP53 | 5 | 2582 | 0.001933 | 3 | 4811 | 0.000623182 | 0.137426 | 1 |
| PTPRD | 41 | 2546 | 0.015848 | 87 | 4727 | 0.018072289 | 0.51393 | 1 |
| PTPRD_TP53 | 26 | 2561 | 0.01005 | 37 | 4777 | 0.007685916 | 0.291236 | 1 |
| PTPRT | 19 | 2568 | 0.007344 | 8 | 4806 | 0.00166182 | 0.000323 | 0.004519 |

Note (the header of table SII-SVI are similar to table SI)：

Gene_Module: genes or combine of several genes with somatic mutations
#mut_mBreast_Cancer: number of samples with somatic mutations of the gene module in metastatic samples.
#wild_mBreast_Cancer: number of samples with wildtype genotype of the gene module in metastatic samples.
mRatio: #mut_mBreast_Cancer/(#mut_mBreast_Cancer+#wild_mBreast_Cancer)
#mut_earlyBreast_Cancer: number of samples with somatic mutations of the gene module in early primary tumor samples.
#wild_earlyBreast_Cancer: number of samples with wildtype genotype of the gene module in early primary tumor samples.
early_Ratio: #mut_earlyBreast_Cancer/(#mut_earlyBreast_Cancer+#wild_earlyBreast_Cancer)
pvalue: the value obtained by Fisher exact test
qvalue: the value obtained by Bonferroni's multiple hypothesis correction of p value

**Supplementary Table S2.**

Differences in the mutational frequency between genes (or gene modules) in early and metastatic Colorectal Cancer (CRC).

| Gene_Module | #mut_mColorectal_Cancer | #wild_mColorectal_Cancer | mRatio | #mut_earlyColorectal_Cancer | #wild_earlyColorectal_Cancer | early_Ratio | pvalue | qvalue |
| --- | --- | --- | --- | --- | --- | --- | --- | --- |
| APC | 1485 | 897 | 0.623426 | 1772 | 1220 | 0.592246 | 0.021208 | 1 |
| APC_ARID1A | 68 | 2314 | 0.028547 | 123 | 2869 | 0.04111 | 0.014282 | 1 |
| APC_ARID1A_BRAF | 9 | 2373 | 0.003778 | 22 | 2970 | 0.007353 | 0.102781 | 1 |
| APC_ARID1A_FBXW7 | 5 | 2377 | 0.002099 | 34 | 2958 | 0.011364 | 3.26E-05 | 0.010044 |
| APC_ARID1A_KRAS | 26 | 2356 | 0.010915 | 60 | 2932 | 0.020053 | 0.008414 | 1 |
| APC_ARID1A_KRAS_PIK3CA | 10 | 2372 | 0.004198 | 28 | 2964 | 0.009358 | 0.031907 | 1 |
| APC_ARID1A_KRAS_TP53 | 17 | 2365 | 0.007137 | 30 | 2962 | 0.010027 | 0.302759 | 1 |
| APC_ARID1A_PIK3CA | 17 | 2365 | 0.007137 | 50 | 2942 | 0.016711 | 0.001779 | 0.548034 |
| APC_ARID1A_PIK3CA_TP53 | 9 | 2373 | 0.003778 | 24 | 2968 | 0.008021 | 0.053434 | 1 |
| APC_ARID1A_PTEN | 7 | 2375 | 0.002939 | 21 | 2971 | 0.007019 | 0.054597 | 1 |
| APC_ARID1A_PTEN_TP53 | 5 | 2377 | 0.002099 | 9 | 2983 | 0.003008 | 0.597978 | 1 |
| APC_ARID1A_SMAD4 | 6 | 2376 | 0.002519 | 22 | 2970 | 0.007353 | 0.020376 | 1 |
| APC_ARID1A_SMAD4_TP53 | 5 | 2377 | 0.002099 | 11 | 2981 | 0.003676 | 0.325541 | 1 |
| APC_ARID1A_SOX9 | 9 | 2373 | 0.003778 | 32 | 2960 | 0.010695 | 0.004023 | 1 |
| APC_ARID1A_TCF7L2 | 7 | 2375 | 0.002939 | 26 | 2966 | 0.00869 | 0.007724 | 1 |
| APC_ARID1A_TCF7L2_TP53 | 6 | 2376 | 0.002519 | 12 | 2980 | 0.004011 | 0.4772 | 1 |
| APC_ARID1A_TP53 | 48 | 2334 | 0.020151 | 65 | 2927 | 0.021725 | 0.703162 | 1 |
| APC_BRAF | 85 | 2297 | 0.035684 | 126 | 2866 | 0.042112 | 0.257964 | 1 |
| APC_BRAF_FBXW7 | 10 | 2372 | 0.004198 | 25 | 2967 | 0.008356 | 0.062315 | 1 |
| APC_BRAF_FBXW7_PIK3CA | 6 | 2376 | 0.002519 | 15 | 2977 | 0.005013 | 0.187385 | 1 |
| APC_BRAF_FBXW7_TP53 | 6 | 2376 | 0.002519 | 9 | 2983 | 0.003008 | 0.800031 | 1 |
| APC_BRAF_KRAS | 11 | 2371 | 0.004618 | 22 | 2970 | 0.007353 | 0.222563 | 1 |
| APC_BRAF_KRAS_PIK3CA | 7 | 2375 | 0.002939 | 10 | 2982 | 0.003342 | 1 | 1 |
| APC_BRAF_KRAS_TP53 | 6 | 2376 | 0.002519 | 9 | 2983 | 0.003008 | 0.800031 | 1 |
| APC_BRAF_NRAS | 5 | 2377 | 0.002099 | 5 | 2987 | 0.001671 | 0.758373 | 1 |
| APC_BRAF_PIK3CA | 31 | 2351 | 0.013014 | 45 | 2947 | 0.01504 | 0.562618 | 1 |
| APC_BRAF_PIK3CA_PTEN | 5 | 2377 | 0.002099 | 13 | 2979 | 0.004345 | 0.234025 | 1 |
| APC_BRAF_PIK3CA_SMAD4 | 7 | 2375 | 0.002939 | 12 | 2980 | 0.004011 | 0.6454 | 1 |
| APC_BRAF_PIK3CA_TP53 | 20 | 2362 | 0.008396 | 24 | 2968 | 0.008021 | 0.880208 | 1 |
| APC_BRAF_PTEN | 7 | 2375 | 0.002939 | 21 | 2971 | 0.007019 | 0.054597 | 1 |
| APC_BRAF_SMAD4 | 17 | 2365 | 0.007137 | 25 | 2967 | 0.008356 | 0.643497 | 1 |
| APC_BRAF_SMAD4_TP53 | 10 | 2372 | 0.004198 | 14 | 2978 | 0.004679 | 0.839697 | 1 |
| APC_BRAF_SOX9 | 8 | 2374 | 0.003359 | 16 | 2976 | 0.005348 | 0.309596 | 1 |
| APC_BRAF_TP53 | 54 | 2328 | 0.02267 | 67 | 2925 | 0.022393 | 1 | 1 |
| APC_CTNNB1 | 32 | 2350 | 0.013434 | 63 | 2929 | 0.021056 | 0.037181 | 1 |
| APC_CTNNB1_KRAS | 15 | 2367 | 0.006297 | 31 | 2961 | 0.010361 | 0.135498 | 1 |
| APC_CTNNB1_KRAS_PIK3CA | 6 | 2376 | 0.002519 | 17 | 2975 | 0.005682 | 0.093024 | 1 |
| APC_CTNNB1_KRAS_TP53 | 8 | 2374 | 0.003359 | 14 | 2978 | 0.004679 | 0.523168 | 1 |
| APC_CTNNB1_PIK3CA | 9 | 2373 | 0.003778 | 29 | 2963 | 0.009693 | 0.012937 | 1 |
| APC_CTNNB1_SMAD4 | 5 | 2377 | 0.002099 | 16 | 2976 | 0.005348 | 0.07679 | 1 |
| APC_CTNNB1_TP53 | 21 | 2361 | 0.008816 | 29 | 2963 | 0.009693 | 0.776544 | 1 |
| APC_FBXW7 | 130 | 2252 | 0.054576 | 238 | 2754 | 0.079545 | 0.000324 | 0.099743 |
| APC_FBXW7_KRAS | 79 | 2303 | 0.033165 | 137 | 2855 | 0.045789 | 0.020923 | 1 |
| APC_FBXW7_KRAS_PIK3CA | 25 | 2357 | 0.010495 | 49 | 2943 | 0.016377 | 0.076789 | 1 |
| APC_FBXW7_KRAS_PIK3CA_SMAD4 | 8 | 2374 | 0.003359 | 10 | 2982 | 0.003342 | 1 | 1 |
| APC_FBXW7_KRAS_PIK3CA_SMAD4_TP53 | 5 | 2377 | 0.002099 | 6 | 2986 | 0.002005 | 1 | 1 |
| APC_FBXW7_KRAS_PIK3CA_TP53 | 14 | 2368 | 0.005877 | 22 | 2970 | 0.007353 | 0.61437 | 1 |
| APC_FBXW7_KRAS_PTEN | 6 | 2376 | 0.002519 | 16 | 2976 | 0.005348 | 0.132873 | 1 |
| APC_FBXW7_KRAS_SMAD4 | 14 | 2368 | 0.005877 | 27 | 2965 | 0.009024 | 0.209084 | 1 |
| APC_FBXW7_KRAS_SMAD4_TP53 | 10 | 2372 | 0.004198 | 17 | 2975 | 0.005682 | 0.561178 | 1 |
| APC_FBXW7_KRAS_SOX9 | 6 | 2376 | 0.002519 | 13 | 2979 | 0.004345 | 0.355897 | 1 |
| APC_FBXW7_KRAS_TP53 | 50 | 2332 | 0.020991 | 78 | 2914 | 0.02607 | 0.242317 | 1 |
| APC_FBXW7_NRAS | 5 | 2377 | 0.002099 | 20 | 2972 | 0.006684 | 0.014842 | 1 |
| APC_FBXW7_PIK3CA | 36 | 2346 | 0.015113 | 81 | 2911 | 0.027072 | 0.003368 | 1 |
| APC_FBXW7_PIK3CA_PTEN | 5 | 2377 | 0.002099 | 17 | 2975 | 0.005682 | 0.051608 | 1 |
| APC_FBXW7_PIK3CA_SMAD4 | 9 | 2373 | 0.003778 | 18 | 2974 | 0.006016 | 0.331832 | 1 |
| APC_FBXW7_PIK3CA_SMAD4_TP53 | 5 | 2377 | 0.002099 | 12 | 2980 | 0.004011 | 0.233884 | 1 |
| APC_FBXW7_PIK3CA_SOX9 | 5 | 2377 | 0.002099 | 11 | 2981 | 0.003676 | 0.325541 | 1 |
| APC_FBXW7_PIK3CA_TP53 | 22 | 2360 | 0.009236 | 42 | 2950 | 0.014037 | 0.128397 | 1 |
| APC_FBXW7_PTEN | 7 | 2375 | 0.002939 | 26 | 2966 | 0.00869 | 0.007724 | 1 |
| APC_FBXW7_SMAD4 | 16 | 2366 | 0.006717 | 42 | 2950 | 0.014037 | 0.011081 | 1 |
| APC_FBXW7_SMAD4_TP53 | 11 | 2371 | 0.004618 | 29 | 2963 | 0.009693 | 0.037073 | 1 |
| APC_FBXW7_SOX9 | 10 | 2372 | 0.004198 | 24 | 2968 | 0.008021 | 0.085255 | 1 |
| APC_FBXW7_SOX9_TP53 | 7 | 2375 | 0.002939 | 10 | 2982 | 0.003342 | 1 | 1 |
| APC_FBXW7_TCF7L2 | 6 | 2376 | 0.002519 | 22 | 2970 | 0.007353 | 0.020376 | 1 |
| APC_FBXW7_TCF7L2_TP53 | 5 | 2377 | 0.002099 | 12 | 2980 | 0.004011 | 0.233884 | 1 |
| APC_FBXW7_TP53 | 95 | 2287 | 0.039882 | 151 | 2841 | 0.050468 | 0.066132 | 1 |
| APC_KRAS | 715 | 1667 | 0.300168 | 885 | 2107 | 0.295789 | 0.741199 | 1 |
| APC_KRAS_NRAS | 5 | 2377 | 0.002099 | 18 | 2974 | 0.006016 | 0.03434 | 1 |
| APC_KRAS_PIK3CA | 175 | 2207 | 0.073468 | 235 | 2757 | 0.078543 | 0.501614 | 1 |
| APC_KRAS_PIK3CA_PTEN | 10 | 2372 | 0.004198 | 22 | 2970 | 0.007353 | 0.155194 | 1 |
| APC_KRAS_PIK3CA_SMAD4 | 42 | 2340 | 0.017632 | 49 | 2943 | 0.016377 | 0.750158 | 1 |
| APC_KRAS_PIK3CA_SMAD4_SOX9 | 7 | 2375 | 0.002939 | 6 | 2986 | 0.002005 | 0.58047 | 1 |
| APC_KRAS_PIK3CA_SMAD4_TP53 | 17 | 2365 | 0.007137 | 18 | 2974 | 0.006016 | 0.613575 | 1 |
| APC_KRAS_PIK3CA_SOX9 | 22 | 2360 | 0.009236 | 25 | 2967 | 0.008356 | 0.769149 | 1 |
| APC_KRAS_PIK3CA_SOX9_TP53 | 5 | 2377 | 0.002099 | 9 | 2983 | 0.003008 | 0.597978 | 1 |
| APC_KRAS_PIK3CA_TCF7L2 | 12 | 2370 | 0.005038 | 21 | 2971 | 0.007019 | 0.384808 | 1 |
| APC_KRAS_PIK3CA_TP53 | 71 | 2311 | 0.029807 | 114 | 2878 | 0.038102 | 0.113536 | 1 |
| APC_KRAS_PTEN | 37 | 2345 | 0.015533 | 50 | 2942 | 0.016711 | 0.745728 | 1 |
| APC_KRAS_PTEN_SMAD4 | 8 | 2374 | 0.003359 | 15 | 2977 | 0.005013 | 0.4057 | 1 |
| APC_KRAS_PTEN_TP53 | 22 | 2360 | 0.009236 | 22 | 2970 | 0.007353 | 0.450867 | 1 |
| APC_KRAS_PTPN11 | 6 | 2376 | 0.002519 | 18 | 2974 | 0.006016 | 0.064407 | 1 |
| APC_KRAS_PTPN11_TP53 | 6 | 2376 | 0.002519 | 10 | 2982 | 0.003342 | 0.624806 | 1 |
| APC_KRAS_PTPRD | 10 | 2372 | 0.004198 | 19 | 2973 | 0.00635 | 0.350057 | 1 |
| APC_KRAS_PTPRD_TP53 | 8 | 2374 | 0.003359 | 11 | 2981 | 0.003676 | 1 | 1 |
| APC_KRAS_PTPRT | 16 | 2366 | 0.006717 | 4 | 2988 | 0.001337 | 0.002338 | 0.720122 |
| APC_KRAS_PTPRT_TP53 | 13 | 2369 | 0.005458 | 3 | 2989 | 0.001003 | 0.00405 | 1 |
| APC_KRAS_SMAD4 | 120 | 2262 | 0.050378 | 163 | 2829 | 0.054479 | 0.538843 | 1 |
| APC_KRAS_SMAD4_SOX9 | 16 | 2366 | 0.006717 | 11 | 2981 | 0.003676 | 0.124489 | 1 |
| APC_KRAS_SMAD4_TP53 | 68 | 2314 | 0.028547 | 84 | 2908 | 0.028075 | 0.934121 | 1 |
| APC_KRAS_SOX9 | 61 | 2321 | 0.025609 | 73 | 2919 | 0.024398 | 0.79213 | 1 |
| APC_KRAS_SOX9_TP53 | 32 | 2350 | 0.013434 | 31 | 2961 | 0.010361 | 0.309817 | 1 |
| APC_KRAS_TCF7L2 | 34 | 2348 | 0.014274 | 65 | 2927 | 0.021725 | 0.051892 | 1 |
| APC_KRAS_TCF7L2_TP53 | 16 | 2366 | 0.006717 | 32 | 2960 | 0.010695 | 0.144667 | 1 |
| APC_KRAS_TP53 | 459 | 1923 | 0.192695 | 523 | 2469 | 0.174799 | 0.094973 | 1 |
| APC_NRAS | 69 | 2313 | 0.028967 | 83 | 2909 | 0.027741 | 0.804142 | 1 |
| APC_NRAS_PIK3CA | 13 | 2369 | 0.005458 | 18 | 2974 | 0.006016 | 0.857407 | 1 |
| APC_NRAS_PIK3CA_TP53 | 6 | 2376 | 0.002519 | 12 | 2980 | 0.004011 | 0.4772 | 1 |
| APC_NRAS_SMAD4 | 8 | 2374 | 0.003359 | 17 | 2975 | 0.005682 | 0.232894 | 1 |
| APC_NRAS_SMAD4_TP53 | 6 | 2376 | 0.002519 | 13 | 2979 | 0.004345 | 0.355897 | 1 |
| APC_NRAS_TP53 | 50 | 2332 | 0.020991 | 66 | 2926 | 0.022059 | 0.850273 | 1 |
| APC_PIK3CA | 262 | 2120 | 0.109992 | 383 | 2609 | 0.128008 | 0.047014 | 1 |
| APC_PIK3CA_PTEN | 16 | 2366 | 0.006717 | 43 | 2949 | 0.014372 | 0.008024 | 1 |
| APC_PIK3CA_PTEN_SMAD4 | 5 | 2377 | 0.002099 | 13 | 2979 | 0.004345 | 0.234025 | 1 |
| APC_PIK3CA_PTEN_TP53 | 7 | 2375 | 0.002939 | 18 | 2974 | 0.006016 | 0.109837 | 1 |
| APC_PIK3CA_PTPRT | 5 | 2377 | 0.002099 | 6 | 2986 | 0.002005 | 1 | 1 |
| APC_PIK3CA_PTPRT_TP53 | 5 | 2377 | 0.002099 | 5 | 2987 | 0.001671 | 0.758373 | 1 |
| APC_PIK3CA_SMAD4 | 50 | 2332 | 0.020991 | 76 | 2916 | 0.025401 | 0.318496 | 1 |
| APC_PIK3CA_SMAD4_SOX9 | 7 | 2375 | 0.002939 | 11 | 2981 | 0.003676 | 0.813113 | 1 |
| APC_PIK3CA_SMAD4_TP53 | 22 | 2360 | 0.009236 | 33 | 2959 | 0.011029 | 0.586081 | 1 |
| APC_PIK3CA_SOX9 | 27 | 2355 | 0.011335 | 41 | 2951 | 0.013703 | 0.46354 | 1 |
| APC_PIK3CA_SOX9_TP53 | 7 | 2375 | 0.002939 | 17 | 2975 | 0.005682 | 0.152951 | 1 |
| APC_PIK3CA_TCF7L2 | 17 | 2365 | 0.007137 | 34 | 2958 | 0.011364 | 0.120864 | 1 |
| APC_PIK3CA_TCF7L2_TP53 | 6 | 2376 | 0.002519 | 15 | 2977 | 0.005013 | 0.187385 | 1 |
| APC_PIK3CA_TP53 | 140 | 2242 | 0.058774 | 213 | 2779 | 0.07119 | 0.076012 | 1 |
| APC_PTEN | 54 | 2328 | 0.02267 | 91 | 2901 | 0.030414 | 0.090033 | 1 |
| APC_PTEN_SMAD4 | 9 | 2373 | 0.003778 | 28 | 2964 | 0.009358 | 0.018841 | 1 |
| APC_PTEN_TP53 | 34 | 2348 | 0.014274 | 41 | 2951 | 0.013703 | 0.907012 | 1 |
| APC_PTPN11 | 11 | 2371 | 0.004618 | 30 | 2962 | 0.010027 | 0.026579 | 1 |
| APC_PTPN11_TP53 | 10 | 2372 | 0.004198 | 15 | 2977 | 0.005013 | 0.6926 | 1 |
| APC_PTPRD | 18 | 2364 | 0.007557 | 33 | 2959 | 0.011029 | 0.205202 | 1 |
| APC_PTPRD_SMAD4 | 6 | 2376 | 0.002519 | 3 | 2989 | 0.001003 | 0.197213 | 1 |
| APC_PTPRD_TP53 | 14 | 2368 | 0.005877 | 17 | 2975 | 0.005682 | 1 | 1 |
| APC_PTPRT | 38 | 2344 | 0.015953 | 8 | 2984 | 0.002674 | 1.10E-07 | 3.40E-05 |
| APC_PTPRT_SMAD4 | 8 | 2374 | 0.003359 | 2 | 2990 | 0.000668 | 0.027489 | 1 |
| APC_PTPRT_SMAD4_TP53 | 8 | 2374 | 0.003359 | 2 | 2990 | 0.000668 | 0.027489 | 1 |
| APC_PTPRT_TP53 | 32 | 2350 | 0.013434 | 7 | 2985 | 0.00234 | 1.96E-06 | 0.000603 |
| APC_SMAD4 | 193 | 2189 | 0.081024 | 254 | 2738 | 0.084893 | 0.619391 | 1 |
| APC_SMAD4_SOX9 | 21 | 2361 | 0.008816 | 26 | 2966 | 0.00869 | 1 | 1 |
| APC_SMAD4_SOX9_TP53 | 7 | 2375 | 0.002939 | 10 | 2982 | 0.003342 | 1 | 1 |
| APC_SMAD4_TCF7L2 | 10 | 2372 | 0.004198 | 24 | 2968 | 0.008021 | 0.085255 | 1 |
| APC_SMAD4_TCF7L2_TP53 | 8 | 2374 | 0.003359 | 12 | 2980 | 0.004011 | 0.822942 | 1 |
| APC_SMAD4_TP53 | 125 | 2257 | 0.052477 | 147 | 2845 | 0.049131 | 0.61639 | 1 |
| APC_SOX9 | 102 | 2280 | 0.042821 | 141 | 2851 | 0.047126 | 0.467801 | 1 |
| APC_SOX9_TP53 | 61 | 2321 | 0.025609 | 70 | 2922 | 0.023396 | 0.656359 | 1 |
| APC_TCF7L2 | 77 | 2305 | 0.032326 | 113 | 2879 | 0.037767 | 0.298461 | 1 |
| APC_TCF7L2_TP53 | 55 | 2327 | 0.02309 | 67 | 2925 | 0.022393 | 0.926604 | 1 |
| APC_TP53 | 1100 | 1282 | 0.461797 | 1206 | 1786 | 0.403075 | 1.70E-05 | 0.00524 |
| ARID1A | 82 | 2300 | 0.034425 | 179 | 2813 | 0.059826 | 1.70E-05 | 0.005223 |
| ARID1A_BRAF | 12 | 2370 | 0.005038 | 47 | 2945 | 0.015709 | 0.000171 | 0.052757 |
| ARID1A_BRAF_TP53 | 5 | 2377 | 0.002099 | 16 | 2976 | 0.005348 | 0.07679 | 1 |
| ARID1A_FBXW7 | 5 | 2377 | 0.002099 | 52 | 2940 | 0.01738 | 7.18E-09 | 2.21E-06 |
| ARID1A_KRAS | 30 | 2352 | 0.012594 | 78 | 2914 | 0.02607 | 0.000401 | 0.123583 |
| ARID1A_KRAS_PIK3CA | 12 | 2370 | 0.005038 | 30 | 2962 | 0.010027 | 0.042703 | 1 |
| ARID1A_KRAS_PIK3CA_TP53 | 6 | 2376 | 0.002519 | 13 | 2979 | 0.004345 | 0.355897 | 1 |
| ARID1A_KRAS_TP53 | 20 | 2362 | 0.008396 | 38 | 2954 | 0.012701 | 0.144505 | 1 |
| ARID1A_PIK3CA | 21 | 2361 | 0.008816 | 61 | 2931 | 0.020388 | 0.00048 | 0.147914 |
| ARID1A_PIK3CA_TP53 | 12 | 2370 | 0.005038 | 27 | 2965 | 0.009024 | 0.10541 | 1 |
| ARID1A_PTEN | 8 | 2374 | 0.003359 | 30 | 2962 | 0.010027 | 0.004659 | 1 |
| ARID1A_PTEN_TP53 | 5 | 2377 | 0.002099 | 9 | 2983 | 0.003008 | 0.597978 | 1 |
| ARID1A_SMAD4 | 11 | 2371 | 0.004618 | 31 | 2961 | 0.010361 | 0.01892 | 1 |
| ARID1A_SMAD4_TP53 | 8 | 2374 | 0.003359 | 17 | 2975 | 0.005682 | 0.232894 | 1 |
| ARID1A_SOX9 | 10 | 2372 | 0.004198 | 50 | 2942 | 0.016711 | 9.45E-06 | 0.002911 |
| ARID1A_TCF7L2 | 8 | 2374 | 0.003359 | 31 | 2961 | 0.010361 | 0.003071 | 0.945902 |
| ARID1A_TCF7L2_TP53 | 6 | 2376 | 0.002519 | 13 | 2979 | 0.004345 | 0.355897 | 1 |
| ARID1A_TP53 | 57 | 2325 | 0.023929 | 84 | 2908 | 0.028075 | 0.390425 | 1 |
| BRAF | 219 | 2163 | 0.09194 | 340 | 2652 | 0.113636 | 0.010319 | 1 |
| BRAF_CTNNB1 | 5 | 2377 | 0.002099 | 22 | 2970 | 0.007353 | 0.006258 | 1 |
| BRAF_FBXW7 | 16 | 2366 | 0.006717 | 50 | 2942 | 0.016711 | 0.001034 | 0.31857 |
| BRAF_FBXW7_PIK3CA | 9 | 2373 | 0.003778 | 21 | 2971 | 0.007019 | 0.140387 | 1 |
| BRAF_FBXW7_TP53 | 9 | 2373 | 0.003778 | 18 | 2974 | 0.006016 | 0.331832 | 1 |
| BRAF_KRAS | 20 | 2362 | 0.008396 | 33 | 2959 | 0.011029 | 0.404728 | 1 |
| BRAF_KRAS_PIK3CA | 9 | 2373 | 0.003778 | 12 | 2980 | 0.004011 | 1 | 1 |
| BRAF_KRAS_TP53 | 13 | 2369 | 0.005458 | 14 | 2978 | 0.004679 | 0.702074 | 1 |
| BRAF_NRAS | 8 | 2374 | 0.003359 | 6 | 2986 | 0.002005 | 0.421794 | 1 |
| BRAF_NRAS_TP53 | 5 | 2377 | 0.002099 | 4 | 2988 | 0.001337 | 0.521644 | 1 |
| BRAF_PIK3CA | 45 | 2337 | 0.018892 | 83 | 2909 | 0.027741 | 0.038137 | 1 |
| BRAF_PIK3CA_PTEN | 6 | 2376 | 0.002519 | 20 | 2972 | 0.006684 | 0.030036 | 1 |
| BRAF_PIK3CA_SMAD4 | 7 | 2375 | 0.002939 | 19 | 2973 | 0.00635 | 0.078047 | 1 |
| BRAF_PIK3CA_SOX9 | 5 | 2377 | 0.002099 | 12 | 2980 | 0.004011 | 0.233884 | 1 |
| BRAF_PIK3CA_TP53 | 28 | 2354 | 0.011755 | 38 | 2954 | 0.012701 | 0.803864 | 1 |
| BRAF_PTEN | 20 | 2362 | 0.008396 | 54 | 2938 | 0.018048 | 0.002968 | 0.914151 |
| BRAF_PTEN_SMAD4 | 6 | 2376 | 0.002519 | 17 | 2975 | 0.005682 | 0.093024 | 1 |
| BRAF_PTEN_SMAD4_TP53 | 5 | 2377 | 0.002099 | 9 | 2983 | 0.003008 | 0.597978 | 1 |
| BRAF_PTEN_TP53 | 13 | 2369 | 0.005458 | 22 | 2970 | 0.007353 | 0.495324 | 1 |
| BRAF_PTPRD | 9 | 2373 | 0.003778 | 15 | 2977 | 0.005013 | 0.54334 | 1 |
| BRAF_PTPRD_TP53 | 7 | 2375 | 0.002939 | 5 | 2987 | 0.001671 | 0.389695 | 1 |
| BRAF_PTPRT | 6 | 2376 | 0.002519 | 3 | 2989 | 0.001003 | 0.197213 | 1 |
| BRAF_PTPRT_TP53 | 5 | 2377 | 0.002099 | 2 | 2990 | 0.000668 | 0.252969 | 1 |
| BRAF_SMAD4 | 45 | 2337 | 0.018892 | 69 | 2923 | 0.023061 | 0.340688 | 1 |
| BRAF_SMAD4_TP53 | 30 | 2352 | 0.012594 | 44 | 2948 | 0.014706 | 0.556745 | 1 |
| BRAF_SOX9 | 12 | 2370 | 0.005038 | 34 | 2958 | 0.011364 | 0.01615 | 1 |
| BRAF_SOX9_TP53 | 5 | 2377 | 0.002099 | 9 | 2983 | 0.003008 | 0.597978 | 1 |
| BRAF_TP53 | 144 | 2238 | 0.060453 | 176 | 2816 | 0.058824 | 0.816617 | 1 |
| CTNNB1 | 81 | 2301 | 0.034005 | 125 | 2867 | 0.041778 | 0.152668 | 1 |
| CTNNB1_KRAS | 39 | 2343 | 0.016373 | 58 | 2934 | 0.019385 | 0.470535 | 1 |
| CTNNB1_KRAS_PIK3CA | 11 | 2371 | 0.004618 | 28 | 2964 | 0.009358 | 0.051306 | 1 |
| CTNNB1_KRAS_PIK3CA_TP53 | 6 | 2376 | 0.002519 | 14 | 2978 | 0.004679 | 0.260416 | 1 |
| CTNNB1_KRAS_SMAD4 | 5 | 2377 | 0.002099 | 11 | 2981 | 0.003676 | 0.325541 | 1 |
| CTNNB1_KRAS_SOX9 | 5 | 2377 | 0.002099 | 9 | 2983 | 0.003008 | 0.597978 | 1 |
| CTNNB1_KRAS_TP53 | 24 | 2358 | 0.010076 | 28 | 2964 | 0.009358 | 0.781418 | 1 |
| CTNNB1_PIK3CA | 20 | 2362 | 0.008396 | 48 | 2944 | 0.016043 | 0.01369 | 1 |
| CTNNB1_PIK3CA_TP53 | 13 | 2369 | 0.005458 | 22 | 2970 | 0.007353 | 0.495324 | 1 |
| CTNNB1_SMAD4 | 10 | 2372 | 0.004198 | 22 | 2970 | 0.007353 | 0.155194 | 1 |
| CTNNB1_SMAD4_TP53 | 8 | 2374 | 0.003359 | 8 | 2984 | 0.002674 | 0.802051 | 1 |
| CTNNB1_SOX9 | 7 | 2375 | 0.002939 | 19 | 2973 | 0.00635 | 0.078047 | 1 |
| CTNNB1_TP53 | 59 | 2323 | 0.024769 | 61 | 2931 | 0.020388 | 0.306869 | 1 |
| FBXW7 | 163 | 2219 | 0.06843 | 334 | 2658 | 0.111631 | 4.49E-08 | 1.38E-05 |
| FBXW7_KRAS | 94 | 2288 | 0.039463 | 176 | 2816 | 0.058824 | 0.001313 | 0.404528 |
| FBXW7_KRAS_PIK3CA | 25 | 2357 | 0.010495 | 56 | 2936 | 0.018717 | 0.017424 | 1 |
| FBXW7_KRAS_PIK3CA_SMAD4 | 8 | 2374 | 0.003359 | 10 | 2982 | 0.003342 | 1 | 1 |
| FBXW7_KRAS_PIK3CA_SMAD4_TP53 | 5 | 2377 | 0.002099 | 6 | 2986 | 0.002005 | 1 | 1 |
| FBXW7_KRAS_PIK3CA_TP53 | 14 | 2368 | 0.005877 | 24 | 2968 | 0.008021 | 0.413942 | 1 |
| FBXW7_KRAS_PTEN | 6 | 2376 | 0.002519 | 19 | 2973 | 0.00635 | 0.044167 | 1 |
| FBXW7_KRAS_SMAD4 | 16 | 2366 | 0.006717 | 35 | 2957 | 0.011698 | 0.066193 | 1 |
| FBXW7_KRAS_SMAD4_TP53 | 11 | 2371 | 0.004618 | 23 | 2969 | 0.007687 | 0.170152 | 1 |
| FBXW7_KRAS_SOX9 | 6 | 2376 | 0.002519 | 16 | 2976 | 0.005348 | 0.132873 | 1 |
| FBXW7_KRAS_TP53 | 60 | 2322 | 0.025189 | 101 | 2891 | 0.033757 | 0.076168 | 1 |
| FBXW7_NRAS | 5 | 2377 | 0.002099 | 21 | 2971 | 0.007019 | 0.009663 | 1 |
| FBXW7_PIK3CA | 42 | 2340 | 0.017632 | 102 | 2890 | 0.034091 | 0.000172 | 0.053051 |
| FBXW7_PIK3CA_PTEN | 5 | 2377 | 0.002099 | 21 | 2971 | 0.007019 | 0.009663 | 1 |
| FBXW7_PIK3CA_SMAD4 | 9 | 2373 | 0.003778 | 20 | 2972 | 0.006684 | 0.189523 | 1 |
| FBXW7_PIK3CA_SMAD4_TP53 | 5 | 2377 | 0.002099 | 14 | 2978 | 0.004679 | 0.163856 | 1 |
| FBXW7_PIK3CA_SOX9 | 5 | 2377 | 0.002099 | 15 | 2977 | 0.005013 | 0.112935 | 1 |
| FBXW7_PIK3CA_TP53 | 24 | 2358 | 0.010076 | 49 | 2943 | 0.016377 | 0.057098 | 1 |
| FBXW7_PTEN | 9 | 2373 | 0.003778 | 36 | 2956 | 0.012032 | 0.000791 | 0.24349 |
| FBXW7_PTEN_TP53 | 5 | 2377 | 0.002099 | 13 | 2979 | 0.004345 | 0.234025 | 1 |
| FBXW7_SMAD4 | 20 | 2362 | 0.008396 | 55 | 2937 | 0.018382 | 0.002138 | 0.658623 |
| FBXW7_SMAD4_TP53 | 13 | 2369 | 0.005458 | 40 | 2952 | 0.013369 | 0.003336 | 1 |
| FBXW7_SOX9 | 10 | 2372 | 0.004198 | 36 | 2956 | 0.012032 | 0.001634 | 0.503412 |
| FBXW7_SOX9_TP53 | 7 | 2375 | 0.002939 | 13 | 2979 | 0.004345 | 0.501208 | 1 |
| FBXW7_TCF7L2 | 6 | 2376 | 0.002519 | 28 | 2964 | 0.009358 | 0.001507 | 0.464169 |
| FBXW7_TCF7L2_TP53 | 5 | 2377 | 0.002099 | 14 | 2978 | 0.004679 | 0.163856 | 1 |
| FBXW7_TP53 | 113 | 2269 | 0.047439 | 203 | 2789 | 0.067848 | 0.001599 | 0.492414 |
| KRAS | 1057 | 1325 | 0.443745 | 1329 | 1663 | 0.444184 | 0.977961 | 1 |
| KRAS_NRAS | 5 | 2377 | 0.002099 | 21 | 2971 | 0.007019 | 0.009663 | 1 |
| KRAS_PIK3CA | 238 | 2144 | 0.099916 | 324 | 2668 | 0.108289 | 0.323952 | 1 |
| KRAS_PIK3CA_PTEN | 15 | 2367 | 0.006297 | 29 | 2963 | 0.009693 | 0.222445 | 1 |
| KRAS_PIK3CA_PTEN_SMAD4 | 6 | 2376 | 0.002519 | 7 | 2985 | 0.00234 | 1 | 1 |
| KRAS_PIK3CA_PTEN_TP53 | 7 | 2375 | 0.002939 | 15 | 2977 | 0.005013 | 0.285609 | 1 |
| KRAS_PIK3CA_SMAD4 | 55 | 2327 | 0.02309 | 61 | 2931 | 0.020388 | 0.509578 | 1 |
| KRAS_PIK3CA_SMAD4_SOX9 | 7 | 2375 | 0.002939 | 7 | 2985 | 0.00234 | 0.789483 | 1 |
| KRAS_PIK3CA_SMAD4_TP53 | 24 | 2358 | 0.010076 | 21 | 2971 | 0.007019 | 0.231295 | 1 |
| KRAS_PIK3CA_SOX9 | 22 | 2360 | 0.009236 | 30 | 2962 | 0.010027 | 0.888624 | 1 |
| KRAS_PIK3CA_SOX9_TP53 | 5 | 2377 | 0.002099 | 10 | 2982 | 0.003342 | 0.445597 | 1 |
| KRAS_PIK3CA_TCF7L2 | 13 | 2369 | 0.005458 | 22 | 2970 | 0.007353 | 0.495324 | 1 |
| KRAS_PIK3CA_TP53 | 103 | 2279 | 0.043241 | 147 | 2845 | 0.049131 | 0.328343 | 1 |
| KRAS_PTEN | 53 | 2329 | 0.02225 | 66 | 2926 | 0.022059 | 1 | 1 |
| KRAS_PTEN_SMAD4 | 11 | 2371 | 0.004618 | 17 | 2975 | 0.005682 | 0.704021 | 1 |
| KRAS_PTEN_SMAD4_TP53 | 5 | 2377 | 0.002099 | 9 | 2983 | 0.003008 | 0.597978 | 1 |
| KRAS_PTEN_TP53 | 31 | 2351 | 0.013014 | 30 | 2962 | 0.010027 | 0.364364 | 1 |
| KRAS_PTPN11 | 7 | 2375 | 0.002939 | 20 | 2972 | 0.006684 | 0.078537 | 1 |
| KRAS_PTPN11_TP53 | 7 | 2375 | 0.002939 | 10 | 2982 | 0.003342 | 1 | 1 |
| KRAS_PTPRD | 12 | 2370 | 0.005038 | 25 | 2967 | 0.008356 | 0.183652 | 1 |
| KRAS_PTPRD_SMAD4 | 5 | 2377 | 0.002099 | 2 | 2990 | 0.000668 | 0.252969 | 1 |
| KRAS_PTPRD_TP53 | 10 | 2372 | 0.004198 | 14 | 2978 | 0.004679 | 0.839697 | 1 |
| KRAS_PTPRT | 20 | 2362 | 0.008396 | 5 | 2987 | 0.001671 | 0.000378 | 0.116538 |
| KRAS_PTPRT_TP53 | 16 | 2366 | 0.006717 | 3 | 2989 | 0.001003 | 0.000652 | 0.200833 |
| KRAS_SMAD4 | 174 | 2208 | 0.073048 | 227 | 2765 | 0.075869 | 0.714732 | 1 |
| KRAS_SMAD4_SOX9 | 18 | 2364 | 0.007557 | 14 | 2978 | 0.004679 | 0.211595 | 1 |
| KRAS_SMAD4_SOX9_TP53 | 5 | 2377 | 0.002099 | 0 | 2992 | 0 | 0.017069 | 1 |
| KRAS_SMAD4_TP53 | 106 | 2276 | 0.0445 | 124 | 2868 | 0.041444 | 0.587983 | 1 |
| KRAS_SOX9 | 70 | 2312 | 0.029387 | 87 | 2905 | 0.029078 | 1 | 1 |
| KRAS_SOX9_TP53 | 38 | 2344 | 0.015953 | 34 | 2958 | 0.011364 | 0.153165 | 1 |
| KRAS_TCF7L2 | 38 | 2344 | 0.015953 | 71 | 2921 | 0.02373 | 0.051054 | 1 |
| KRAS_TCF7L2_TP53 | 18 | 2364 | 0.007557 | 35 | 2957 | 0.011698 | 0.164078 | 1 |
| KRAS_TP53 | 675 | 1707 | 0.283375 | 758 | 2234 | 0.253342 | 0.014165 | 1 |
| NRAS | 99 | 2283 | 0.041562 | 130 | 2862 | 0.043449 | 0.785762 | 1 |
| NRAS_PIK3CA | 14 | 2368 | 0.005877 | 23 | 2969 | 0.007687 | 0.50761 | 1 |
| NRAS_PIK3CA_TP53 | 6 | 2376 | 0.002519 | 14 | 2978 | 0.004679 | 0.260416 | 1 |
| NRAS_SMAD4 | 13 | 2369 | 0.005458 | 20 | 2972 | 0.006684 | 0.602658 | 1 |
| NRAS_SMAD4_TP53 | 11 | 2371 | 0.004618 | 16 | 2976 | 0.005348 | 0.846564 | 1 |
| NRAS_TP53 | 71 | 2311 | 0.029807 | 94 | 2898 | 0.031417 | 0.750844 | 1 |
| PIK3CA | 372 | 2010 | 0.156171 | 563 | 2429 | 0.188168 | 0.002082 | 0.641398 |
| PIK3CA_PTEN | 22 | 2360 | 0.009236 | 61 | 2931 | 0.020388 | 0.001114 | 0.343101 |
| PIK3CA_PTEN_SMAD4 | 7 | 2375 | 0.002939 | 17 | 2975 | 0.005682 | 0.152951 | 1 |
| PIK3CA_PTEN_TP53 | 11 | 2371 | 0.004618 | 24 | 2968 | 0.008021 | 0.128751 | 1 |
| PIK3CA_PTPN11 | 5 | 2377 | 0.002099 | 16 | 2976 | 0.005348 | 0.07679 | 1 |
| PIK3CA_PTPN11_TP53 | 5 | 2377 | 0.002099 | 10 | 2982 | 0.003342 | 0.445597 | 1 |
| PIK3CA_PTPRT | 5 | 2377 | 0.002099 | 6 | 2986 | 0.002005 | 1 | 1 |
| PIK3CA_PTPRT_TP53 | 5 | 2377 | 0.002099 | 5 | 2987 | 0.001671 | 0.758373 | 1 |
| PIK3CA_SMAD4 | 63 | 2319 | 0.026448 | 101 | 2891 | 0.033757 | 0.129613 | 1 |
| PIK3CA_SMAD4_SOX9 | 7 | 2375 | 0.002939 | 12 | 2980 | 0.004011 | 0.6454 | 1 |
| PIK3CA_SMAD4_TP53 | 29 | 2353 | 0.012175 | 44 | 2948 | 0.014706 | 0.477339 | 1 |
| PIK3CA_SOX9 | 28 | 2354 | 0.011755 | 55 | 2937 | 0.018382 | 0.057977 | 1 |
| PIK3CA_SOX9_TP53 | 8 | 2374 | 0.003359 | 19 | 2973 | 0.00635 | 0.172969 | 1 |
| PIK3CA_TCF7L2 | 19 | 2363 | 0.007976 | 39 | 2953 | 0.013035 | 0.08413 | 1 |
| PIK3CA_TCF7L2_TP53 | 6 | 2376 | 0.002519 | 16 | 2976 | 0.005348 | 0.132873 | 1 |
| PIK3CA_TP53 | 204 | 2178 | 0.085642 | 289 | 2703 | 0.096591 | 0.168195 | 1 |
| PTEN | 86 | 2296 | 0.036104 | 154 | 2838 | 0.051471 | 0.007721 | 1 |
| PTEN_PTPN11 | 5 | 2377 | 0.002099 | 9 | 2983 | 0.003008 | 0.597978 | 1 |
| PTEN_PTPN11_TP53 | 5 | 2377 | 0.002099 | 5 | 2987 | 0.001671 | 0.758373 | 1 |
| PTEN_SMAD4 | 17 | 2365 | 0.007137 | 44 | 2948 | 0.014706 | 0.009342 | 1 |
| PTEN_SMAD4_TP53 | 9 | 2373 | 0.003778 | 22 | 2970 | 0.007353 | 0.102781 | 1 |
| PTEN_SOX9 | 6 | 2376 | 0.002519 | 16 | 2976 | 0.005348 | 0.132873 | 1 |
| PTEN_TP53 | 53 | 2329 | 0.02225 | 67 | 2925 | 0.022393 | 1 | 1 |
| PTPN11 | 15 | 2367 | 0.006297 | 44 | 2948 | 0.014706 | 0.003454 | 1 |
| PTPN11_SMAD4 | 5 | 2377 | 0.002099 | 9 | 2983 | 0.003008 | 0.597978 | 1 |
| PTPN11_TP53 | 11 | 2371 | 0.004618 | 21 | 2971 | 0.007019 | 0.28785 | 1 |
| PTPRD | 29 | 2353 | 0.012175 | 48 | 2944 | 0.016043 | 0.249796 | 1 |
| PTPRD_SMAD4 | 9 | 2373 | 0.003778 | 8 | 2984 | 0.002674 | 0.476564 | 1 |
| PTPRD_SMAD4_TP53 | 5 | 2377 | 0.002099 | 5 | 2987 | 0.001671 | 0.758373 | 1 |
| PTPRD_TP53 | 22 | 2360 | 0.009236 | 23 | 2969 | 0.007687 | 0.549982 | 1 |
| PTPRT | 49 | 2333 | 0.020571 | 10 | 2982 | 0.003342 | 1.01E-09 | 3.12E-07 |
| PTPRT_SMAD4 | 10 | 2372 | 0.004198 | 2 | 2990 | 0.000668 | 0.007739 | 1 |
| PTPRT_SMAD4_TP53 | 9 | 2373 | 0.003778 | 2 | 2990 | 0.000668 | 0.014686 | 1 |
| PTPRT_TP53 | 40 | 2342 | 0.016793 | 7 | 2985 | 0.00234 | 8.40E-09 | 2.59E-06 |
| SMAD4 | 312 | 2070 | 0.130982 | 409 | 2583 | 0.136698 | 0.545965 | 1 |
| SMAD4_SOX9 | 25 | 2357 | 0.010495 | 32 | 2960 | 0.010695 | 1 | 1 |
| SMAD4_SOX9_TP53 | 9 | 2373 | 0.003778 | 12 | 2980 | 0.004011 | 1 | 1 |
| SMAD4_TCF7L2 | 10 | 2372 | 0.004198 | 26 | 2966 | 0.00869 | 0.062573 | 1 |
| SMAD4_TCF7L2_TP53 | 8 | 2374 | 0.003359 | 13 | 2979 | 0.004345 | 0.662682 | 1 |
| SMAD4_TP53 | 207 | 2175 | 0.086902 | 252 | 2740 | 0.084225 | 0.731181 | 1 |
| SOX9 | 123 | 2259 | 0.051637 | 187 | 2805 | 0.0625 | 0.099045 | 1 |
| SOX9_TP53 | 73 | 2309 | 0.030647 | 83 | 2909 | 0.027741 | 0.56718 | 1 |
| TCF7L2 | 82 | 2300 | 0.034425 | 128 | 2864 | 0.042781 | 0.119527 | 1 |
| TCF7L2_TP53 | 57 | 2325 | 0.023929 | 75 | 2917 | 0.025067 | 0.859275 | 1 |
| TP53 | 1668 | 714 | 0.700252 | 1860 | 1132 | 0.621658 | 1.76E-09 | 5.42E-07 |

**Supplementary Table S3.**

Differences in the mutational frequency between genes (or gene modules) in early and metastatic Esophagogastric Cancer (EGC).

| Gene_Module | #mut_mEsophagogastric_Cancer | #wild_mEsophagogastric_Cancer | mRatio | #mut_earlyEsophagogastric_Cancer | #wild_earlyEsophagogastric_Cancer | early_Ratio | pvalue | qvalue |
| --- | --- | --- | --- | --- | --- | --- | --- | --- |
| APC | 35 | 504 | 0.064935 | 72 | 777 | 0.084806 | 0.181464 | 1 |
| APC_KRAS | 5 | 534 | 0.009276 | 10 | 839 | 0.011779 | 0.793163 | 1 |
| APC_TP53 | 26 | 513 | 0.048237 | 54 | 795 | 0.063604 | 0.240213 | 1 |
| ARID1A | 55 | 484 | 0.102041 | 109 | 740 | 0.128386 | 0.14745 | 1 |
| ARID1A_CDH1 | 6 | 533 | 0.011132 | 19 | 830 | 0.022379 | 0.149188 | 1 |
| ARID1A_KRAS | 6 | 533 | 0.011132 | 10 | 839 | 0.011779 | 1 | 1 |
| ARID1A_KRAS_TP53 | 6 | 533 | 0.011132 | 6 | 843 | 0.007067 | 0.553654 | 1 |
| ARID1A_PIK3CA | 5 | 534 | 0.009276 | 18 | 831 | 0.021201 | 0.129267 | 1 |
| ARID1A_TP53 | 43 | 496 | 0.079777 | 57 | 792 | 0.067138 | 0.394965 | 1 |
| ARID2 | 10 | 529 | 0.018553 | 33 | 816 | 0.038869 | 0.038161 | 1 |
| ARID2_TP53 | 6 | 533 | 0.011132 | 20 | 829 | 0.023557 | 0.10719 | 1 |
| CDH1 | 47 | 492 | 0.087199 | 60 | 789 | 0.070671 | 0.258452 | 1 |
| CDH1_TP53 | 32 | 507 | 0.059369 | 29 | 820 | 0.034158 | 0.031104 | 1 |
| CDKN2A | 52 | 487 | 0.096475 | 106 | 743 | 0.124853 | 0.118492 | 1 |
| CDKN2A_KMT2D | 5 | 534 | 0.009276 | 10 | 839 | 0.011779 | 0.793163 | 1 |
| CDKN2A_KMT2D_TP53 | 5 | 534 | 0.009276 | 7 | 842 | 0.008245 | 1 | 1 |
| CDKN2A_KRAS | 5 | 534 | 0.009276 | 9 | 840 | 0.010601 | 1 | 1 |
| CDKN2A_KRAS_TP53 | 5 | 534 | 0.009276 | 5 | 844 | 0.005889 | 0.523388 | 1 |
| CDKN2A_TP53 | 47 | 492 | 0.087199 | 84 | 765 | 0.09894 | 0.510093 | 1 |
| CTNNB1 | 9 | 530 | 0.016698 | 30 | 819 | 0.035336 | 0.045297 | 1 |
| CTNNB1_TP53 | 6 | 533 | 0.011132 | 17 | 832 | 0.020024 | 0.281029 | 1 |
| ERBB2 | 18 | 521 | 0.033395 | 37 | 812 | 0.043581 | 0.397995 | 1 |
| ERBB2_TP53 | 11 | 528 | 0.020408 | 26 | 823 | 0.030624 | 0.305914 | 1 |
| ERBB4 | 30 | 509 | 0.055659 | 58 | 791 | 0.068316 | 0.367911 | 1 |
| ERBB4_TP53 | 24 | 515 | 0.044527 | 40 | 809 | 0.047114 | 0.895856 | 1 |
| FBXW7 | 17 | 522 | 0.03154 | 34 | 815 | 0.040047 | 0.465951 | 1 |
| FBXW7_TP53 | 11 | 528 | 0.020408 | 21 | 828 | 0.024735 | 0.714693 | 1 |
| KMT2D | 19 | 520 | 0.03525 | 86 | 763 | 0.101296 | 3.29E-06 | 0.000155 |
| KMT2D_TP53 | 13 | 526 | 0.024119 | 57 | 792 | 0.067138 | 0.000234 | 0.011001 |
| KRAS | 37 | 502 | 0.068646 | 49 | 800 | 0.057715 | 0.425257 | 1 |
| KRAS_PIK3CA | 5 | 534 | 0.009276 | 7 | 842 | 0.008245 | 1 | 1 |
| KRAS_TP53 | 28 | 511 | 0.051948 | 28 | 821 | 0.03298 | 0.092864 | 1 |
| NOTCH1 | 17 | 522 | 0.03154 | 83 | 766 | 0.097762 | 1.22E-06 | 5.74E-05 |
| NOTCH1_TP53 | 12 | 527 | 0.022263 | 60 | 789 | 0.070671 | 5.27E-05 | 0.002476 |
| PIK3CA | 33 | 506 | 0.061224 | 68 | 781 | 0.080094 | 0.204107 | 1 |
| PIK3CA_TP53 | 19 | 520 | 0.03525 | 30 | 819 | 0.035336 | 1 | 1 |
| PTPRD | 19 | 520 | 0.03525 | 24 | 825 | 0.028269 | 0.525504 | 1 |
| PTPRD_SMAD4 | 5 | 534 | 0.009276 | 0 | 849 | 0 | 0.008731 | 0.410343 |
| PTPRD_SMAD4_TP53 | 5 | 534 | 0.009276 | 0 | 849 | 0 | 0.008731 | 0.410343 |
| PTPRD_TP53 | 16 | 523 | 0.029685 | 20 | 829 | 0.023557 | 0.492545 | 1 |
| PTPRT | 20 | 519 | 0.037106 | 4 | 845 | 0.004711 | 1.53E-05 | 0.000718 |
| PTPRT_TP53 | 15 | 524 | 0.027829 | 4 | 845 | 0.004711 | 0.000486 | 0.022831 |
| RHOA | 13 | 526 | 0.024119 | 5 | 844 | 0.005889 | 0.005734 | 0.269497 |
| RHOA_TP53 | 11 | 528 | 0.020408 | 3 | 846 | 0.003534 | 0.003822 | 0.179626 |
| SMAD4 | 37 | 502 | 0.068646 | 55 | 794 | 0.064782 | 0.824975 | 1 |
| SMAD4_TP53 | 31 | 508 | 0.057514 | 43 | 806 | 0.050648 | 0.624349 | 1 |
| TP53 | 378 | 161 | 0.701299 | 580 | 269 | 0.683157 | 0.512461 | 1 |

**Supplementary Table S4.**

Differences in the mutational frequency between genes (or gene modules) in early and metastatic Non-Small Cell Lung Cancer (NSCLC).

| Gene_Module | #mut_mNon.Small_Cell_Lung_Cancer | #wild_mNon.Small_Cell_Lung_Cancer | mRatio | #mut_earlyNon.Small_Cell_Lung_Cancer | #wild_earlyNon.Small_Cell_Lung_Cancer | early_Ratio | pvalue | qvalue |
| --- | --- | --- | --- | --- | --- | --- | --- | --- |
| ARID1A | 191 | 3107 | 0.057914 | 182 | 3830 | 0.045364 | 0.016206 | 1 |
| ARID1A_ATM | 34 | 3264 | 0.010309 | 27 | 3985 | 0.00673 | 0.120342 | 1 |
| ARID1A_ATM_KEAP1 | 7 | 3291 | 0.002122 | 1 | 4011 | 0.000249 | 0.026579 | 1 |
| ARID1A_ATM_KRAS | 14 | 3284 | 0.004245 | 10 | 4002 | 0.002493 | 0.22043 | 1 |
| ARID1A_ATM_KRAS_TP53 | 7 | 3291 | 0.002122 | 3 | 4009 | 0.000748 | 0.201315 | 1 |
| ARID1A_ATM_NF1 | 6 | 3292 | 0.001819 | 5 | 4007 | 0.001246 | 0.558601 | 1 |
| ARID1A_ATM_NF1_TP53 | 5 | 3293 | 0.001516 | 1 | 4011 | 0.000249 | 0.097192 | 1 |
| ARID1A_ATM_SMARCA4 | 5 | 3293 | 0.001516 | 2 | 4010 | 0.000499 | 0.255537 | 1 |
| ARID1A_ATM_STK11 | 9 | 3289 | 0.002729 | 4 | 4008 | 0.000997 | 0.09713 | 1 |
| ARID1A_ATM_STK11_TP53 | 5 | 3293 | 0.001516 | 3 | 4009 | 0.000748 | 0.480506 | 1 |
| ARID1A_ATM_TP53 | 23 | 3275 | 0.006974 | 12 | 4000 | 0.002991 | 0.016608 | 1 |
| ARID1A_BRAF | 14 | 3284 | 0.004245 | 8 | 4004 | 0.001994 | 0.088998 | 1 |
| ARID1A_BRAF_KRAS | 5 | 3293 | 0.001516 | 1 | 4011 | 0.000249 | 0.097192 | 1 |
| ARID1A_BRAF_NF1 | 6 | 3292 | 0.001819 | 0 | 4012 | 0 | 0.008412 | 1 |
| ARID1A_BRAF_NF1_TP53 | 5 | 3293 | 0.001516 | 0 | 4012 | 0 | 0.018661 | 1 |
| ARID1A_BRAF_SETD2 | 5 | 3293 | 0.001516 | 4 | 4008 | 0.000997 | 0.739872 | 1 |
| ARID1A_BRAF_TP53 | 10 | 3288 | 0.003032 | 4 | 4008 | 0.000997 | 0.059853 | 1 |
| ARID1A_CDKN2A | 30 | 3268 | 0.009096 | 21 | 3991 | 0.005234 | 0.065357 | 1 |
| ARID1A_CDKN2A_KEAP1 | 10 | 3288 | 0.003032 | 5 | 4007 | 0.001246 | 0.119521 | 1 |
| ARID1A_CDKN2A_KEAP1_TP53 | 10 | 3288 | 0.003032 | 5 | 4007 | 0.001246 | 0.119521 | 1 |
| ARID1A_CDKN2A_KMT2D | 6 | 3292 | 0.001819 | 6 | 4006 | 0.001496 | 0.777757 | 1 |
| ARID1A_CDKN2A_KMT2D_TP53 | 6 | 3292 | 0.001819 | 5 | 4007 | 0.001246 | 0.558601 | 1 |
| ARID1A_CDKN2A_KRAS | 6 | 3292 | 0.001819 | 6 | 4006 | 0.001496 | 0.777757 | 1 |
| ARID1A_CDKN2A_KRAS_TP53 | 5 | 3293 | 0.001516 | 2 | 4010 | 0.000499 | 0.255537 | 1 |
| ARID1A_CDKN2A_NF1 | 8 | 3290 | 0.002426 | 3 | 4009 | 0.000748 | 0.075396 | 1 |
| ARID1A_CDKN2A_NF1_TP53 | 7 | 3291 | 0.002122 | 2 | 4010 | 0.000499 | 0.088269 | 1 |
| ARID1A_CDKN2A_PIK3CA | 5 | 3293 | 0.001516 | 0 | 4012 | 0 | 0.018661 | 1 |
| ARID1A_CDKN2A_PIK3CA_TP53 | 5 | 3293 | 0.001516 | 0 | 4012 | 0 | 0.018661 | 1 |
| ARID1A_CDKN2A_RBM10 | 6 | 3292 | 0.001819 | 0 | 4012 | 0 | 0.008412 | 1 |
| ARID1A_CDKN2A_RBM10_TP53 | 6 | 3292 | 0.001819 | 0 | 4012 | 0 | 0.008412 | 1 |
| ARID1A_CDKN2A_SMARCA4 | 7 | 3291 | 0.002122 | 4 | 4008 | 0.000997 | 0.239779 | 1 |
| ARID1A_CDKN2A_SMARCA4_TP53 | 6 | 3292 | 0.001819 | 3 | 4009 | 0.000748 | 0.315191 | 1 |
| ARID1A_CDKN2A_STK11 | 5 | 3293 | 0.001516 | 3 | 4009 | 0.000748 | 0.480506 | 1 |
| ARID1A_CDKN2A_STK11_TP53 | 5 | 3293 | 0.001516 | 2 | 4010 | 0.000499 | 0.255537 | 1 |
| ARID1A_CDKN2A_TP53 | 29 | 3269 | 0.008793 | 14 | 3998 | 0.00349 | 0.003388 | 1 |
| ARID1A_CTNNB1 | 6 | 3292 | 0.001819 | 5 | 4007 | 0.001246 | 0.558601 | 1 |
| ARID1A_CTNNB1_TP53 | 6 | 3292 | 0.001819 | 3 | 4009 | 0.000748 | 0.315191 | 1 |
| ARID1A_EGFR | 30 | 3268 | 0.009096 | 23 | 3989 | 0.005733 | 0.097653 | 1 |
| ARID1A_EGFR_PIK3CA | 8 | 3290 | 0.002426 | 3 | 4009 | 0.000748 | 0.075396 | 1 |
| ARID1A_EGFR_PIK3CA_TP53 | 6 | 3292 | 0.001819 | 2 | 4010 | 0.000499 | 0.151704 | 1 |
| ARID1A_EGFR_TP53 | 25 | 3273 | 0.00758 | 15 | 3997 | 0.003739 | 0.037039 | 1 |
| ARID1A_KEAP1 | 32 | 3266 | 0.009703 | 26 | 3986 | 0.006481 | 0.144756 | 1 |
| ARID1A_KEAP1_KMT2D | 8 | 3290 | 0.002426 | 8 | 4004 | 0.001994 | 0.803112 | 1 |
| ARID1A_KEAP1_KMT2D_TP53 | 5 | 3293 | 0.001516 | 7 | 4005 | 0.001745 | 1 | 1 |
| ARID1A_KEAP1_KRAS | 10 | 3288 | 0.003032 | 5 | 4007 | 0.001246 | 0.119521 | 1 |
| ARID1A_KEAP1_KRAS_STK11 | 7 | 3291 | 0.002122 | 1 | 4011 | 0.000249 | 0.026579 | 1 |
| ARID1A_KEAP1_MGA | 5 | 3293 | 0.001516 | 1 | 4011 | 0.000249 | 0.097192 | 1 |
| ARID1A_KEAP1_NF1 | 7 | 3291 | 0.002122 | 1 | 4011 | 0.000249 | 0.026579 | 1 |
| ARID1A_KEAP1_NF1_TP53 | 6 | 3292 | 0.001819 | 1 | 4011 | 0.000249 | 0.051102 | 1 |
| ARID1A_KEAP1_PIK3CA | 6 | 3292 | 0.001819 | 2 | 4010 | 0.000499 | 0.151704 | 1 |
| ARID1A_KEAP1_SMARCA4 | 5 | 3293 | 0.001516 | 4 | 4008 | 0.000997 | 0.739872 | 1 |
| ARID1A_KEAP1_STK11 | 12 | 3286 | 0.003639 | 6 | 4006 | 0.001496 | 0.09464 | 1 |
| ARID1A_KEAP1_STK11_TP53 | 6 | 3292 | 0.001819 | 3 | 4009 | 0.000748 | 0.315191 | 1 |
| ARID1A_KEAP1_TP53 | 22 | 3276 | 0.006671 | 20 | 3992 | 0.004985 | 0.354913 | 1 |
| ARID1A_KMT2D | 26 | 3272 | 0.007884 | 45 | 3967 | 0.011216 | 0.152656 | 1 |
| ARID1A_KMT2D_KRAS | 5 | 3293 | 0.001516 | 10 | 4002 | 0.002493 | 0.441537 | 1 |
| ARID1A_KMT2D_PTPRD | 5 | 3293 | 0.001516 | 5 | 4007 | 0.001246 | 0.762057 | 1 |
| ARID1A_KMT2D_STK11 | 5 | 3293 | 0.001516 | 9 | 4003 | 0.002243 | 0.595265 | 1 |
| ARID1A_KMT2D_TP53 | 18 | 3280 | 0.005458 | 33 | 3979 | 0.008225 | 0.203351 | 1 |
| ARID1A_KRAS | 61 | 3237 | 0.018496 | 54 | 3958 | 0.01346 | 0.089528 | 1 |
| ARID1A_KRAS_MET | 5 | 3293 | 0.001516 | 2 | 4010 | 0.000499 | 0.255537 | 1 |
| ARID1A_KRAS_NF1 | 9 | 3289 | 0.002729 | 5 | 4007 | 0.001246 | 0.182388 | 1 |
| ARID1A_KRAS_NF1_TP53 | 6 | 3292 | 0.001819 | 2 | 4010 | 0.000499 | 0.151704 | 1 |
| ARID1A_KRAS_PIK3CA | 6 | 3292 | 0.001819 | 5 | 4007 | 0.001246 | 0.558601 | 1 |
| ARID1A_KRAS_PTPRD | 9 | 3289 | 0.002729 | 1 | 4011 | 0.000249 | 0.007044 | 1 |
| ARID1A_KRAS_PTPRD_TP53 | 5 | 3293 | 0.001516 | 1 | 4011 | 0.000249 | 0.097192 | 1 |
| ARID1A_KRAS_RBM10 | 7 | 3291 | 0.002122 | 3 | 4009 | 0.000748 | 0.201315 | 1 |
| ARID1A_KRAS_SETD2 | 7 | 3291 | 0.002122 | 7 | 4005 | 0.001745 | 0.791309 | 1 |
| ARID1A_KRAS_SETD2_TP53 | 5 | 3293 | 0.001516 | 2 | 4010 | 0.000499 | 0.255537 | 1 |
| ARID1A_KRAS_SMARCA4 | 8 | 3290 | 0.002426 | 7 | 4005 | 0.001745 | 0.607342 | 1 |
| ARID1A_KRAS_STK11 | 12 | 3286 | 0.003639 | 7 | 4005 | 0.001745 | 0.164482 | 1 |
| ARID1A_KRAS_STK11_TP53 | 6 | 3292 | 0.001819 | 2 | 4010 | 0.000499 | 0.151704 | 1 |
| ARID1A_KRAS_TP53 | 38 | 3260 | 0.011522 | 19 | 3993 | 0.004736 | 0.0012 | 0.817362 |
| ARID1A_MET | 13 | 3285 | 0.003942 | 16 | 3996 | 0.003988 | 1 | 1 |
| ARID1A_MET_TP53 | 11 | 3287 | 0.003335 | 10 | 4002 | 0.002493 | 0.518426 | 1 |
| ARID1A_MGA | 10 | 3288 | 0.003032 | 5 | 4007 | 0.001246 | 0.119521 | 1 |
| ARID1A_MGA_NF1 | 5 | 3293 | 0.001516 | 1 | 4011 | 0.000249 | 0.097192 | 1 |
| ARID1A_MGA_NF1_TP53 | 5 | 3293 | 0.001516 | 1 | 4011 | 0.000249 | 0.097192 | 1 |
| ARID1A_MGA_TP53 | 9 | 3289 | 0.002729 | 3 | 4009 | 0.000748 | 0.044183 | 1 |
| ARID1A_NF1 | 29 | 3269 | 0.008793 | 23 | 3989 | 0.005733 | 0.126142 | 1 |
| ARID1A_NF1_PIK3CA | 5 | 3293 | 0.001516 | 3 | 4009 | 0.000748 | 0.480506 | 1 |
| ARID1A_NF1_RBM10 | 5 | 3293 | 0.001516 | 2 | 4010 | 0.000499 | 0.255537 | 1 |
| ARID1A_NF1_SMARCA4 | 6 | 3292 | 0.001819 | 1 | 4011 | 0.000249 | 0.051102 | 1 |
| ARID1A_NF1_TP53 | 22 | 3276 | 0.006671 | 16 | 3996 | 0.003988 | 0.140656 | 1 |
| ARID1A_PIK3CA | 19 | 3279 | 0.005761 | 14 | 3998 | 0.00349 | 0.163281 | 1 |
| ARID1A_PIK3CA_TP53 | 13 | 3285 | 0.003942 | 9 | 4003 | 0.002243 | 0.20357 | 1 |
| ARID1A_PTPRD | 20 | 3278 | 0.006064 | 10 | 4002 | 0.002493 | 0.025714 | 1 |
| ARID1A_PTPRD_TP53 | 15 | 3283 | 0.004548 | 10 | 4002 | 0.002493 | 0.159725 | 1 |
| ARID1A_PTPRT | 8 | 3290 | 0.002426 | 0 | 4012 | 0 | 0.001709 | 1 |
| ARID1A_PTPRT_TP53 | 6 | 3292 | 0.001819 | 0 | 4012 | 0 | 0.008412 | 1 |
| ARID1A_RB1 | 16 | 3282 | 0.004851 | 7 | 4005 | 0.001745 | 0.020881 | 1 |
| ARID1A_RB1_TP53 | 15 | 3283 | 0.004548 | 5 | 4007 | 0.001246 | 0.011309 | 1 |
| ARID1A_RBM10 | 16 | 3282 | 0.004851 | 7 | 4005 | 0.001745 | 0.020881 | 1 |
| ARID1A_RBM10_TP53 | 9 | 3289 | 0.002729 | 3 | 4009 | 0.000748 | 0.044183 | 1 |
| ARID1A_SETD2 | 21 | 3277 | 0.006367 | 26 | 3986 | 0.006481 | 1 | 1 |
| ARID1A_SETD2_TP53 | 14 | 3284 | 0.004245 | 12 | 4000 | 0.002991 | 0.431597 | 1 |
| ARID1A_SMARCA4 | 22 | 3276 | 0.006671 | 18 | 3994 | 0.004487 | 0.264529 | 1 |
| ARID1A_SMARCA4_TP53 | 18 | 3280 | 0.005458 | 10 | 4002 | 0.002493 | 0.055465 | 1 |
| ARID1A_STK11 | 26 | 3272 | 0.007884 | 22 | 3990 | 0.005484 | 0.244418 | 1 |
| ARID1A_STK11_TP53 | 18 | 3280 | 0.005458 | 12 | 4000 | 0.002991 | 0.140084 | 1 |
| ARID1A_TP53 | 137 | 3161 | 0.04154 | 103 | 3909 | 0.025673 | 0.000164 | 0.111616 |
| ATM | 239 | 3059 | 0.072468 | 273 | 3739 | 0.068046 | 0.461689 | 1 |
| ATM_BRAF | 16 | 3282 | 0.004851 | 23 | 3989 | 0.005733 | 0.632496 | 1 |
| ATM_BRAF_EGFR | 5 | 3293 | 0.001516 | 4 | 4008 | 0.000997 | 0.739872 | 1 |
| ATM_BRAF_KRAS | 5 | 3293 | 0.001516 | 9 | 4003 | 0.002243 | 0.595265 | 1 |
| ATM_BRAF_TP53 | 6 | 3292 | 0.001819 | 10 | 4002 | 0.002493 | 0.621057 | 1 |
| ATM_CDKN2A | 18 | 3280 | 0.005458 | 21 | 3991 | 0.005234 | 1 | 1 |
| ATM_CDKN2A_KEAP1 | 6 | 3292 | 0.001819 | 3 | 4009 | 0.000748 | 0.315191 | 1 |
| ATM_CDKN2A_KEAP1_TP53 | 6 | 3292 | 0.001819 | 2 | 4010 | 0.000499 | 0.151704 | 1 |
| ATM_CDKN2A_KMT2D | 5 | 3293 | 0.001516 | 5 | 4007 | 0.001246 | 0.762057 | 1 |
| ATM_CDKN2A_KMT2D_TP53 | 5 | 3293 | 0.001516 | 4 | 4008 | 0.000997 | 0.739872 | 1 |
| ATM_CDKN2A_NF1 | 6 | 3292 | 0.001819 | 3 | 4009 | 0.000748 | 0.315191 | 1 |
| ATM_CDKN2A_NF1_TP53 | 6 | 3292 | 0.001819 | 2 | 4010 | 0.000499 | 0.151704 | 1 |
| ATM_CDKN2A_SMARCA4 | 9 | 3289 | 0.002729 | 3 | 4009 | 0.000748 | 0.044183 | 1 |
| ATM_CDKN2A_SMARCA4_TP53 | 8 | 3290 | 0.002426 | 3 | 4009 | 0.000748 | 0.075396 | 1 |
| ATM_CDKN2A_TP53 | 17 | 3281 | 0.005155 | 14 | 3998 | 0.00349 | 0.284119 | 1 |
| ATM_CTNNB1 | 5 | 3293 | 0.001516 | 6 | 4006 | 0.001496 | 1 | 1 |
| ATM_EGFR | 39 | 3259 | 0.011825 | 37 | 3975 | 0.009222 | 0.297855 | 1 |
| ATM_EGFR_KMT2D | 6 | 3292 | 0.001819 | 1 | 4011 | 0.000249 | 0.051102 | 1 |
| ATM_EGFR_KRAS | 8 | 3290 | 0.002426 | 6 | 4006 | 0.001496 | 0.426098 | 1 |
| ATM_EGFR_PIK3CA | 5 | 3293 | 0.001516 | 6 | 4006 | 0.001496 | 1 | 1 |
| ATM_EGFR_PIK3CA_TP53 | 5 | 3293 | 0.001516 | 5 | 4007 | 0.001246 | 0.762057 | 1 |
| ATM_EGFR_PTPRD | 5 | 3293 | 0.001516 | 0 | 4012 | 0 | 0.018661 | 1 |
| ATM_EGFR_RB1 | 9 | 3289 | 0.002729 | 4 | 4008 | 0.000997 | 0.09713 | 1 |
| ATM_EGFR_RB1_TP53 | 7 | 3291 | 0.002122 | 3 | 4009 | 0.000748 | 0.201315 | 1 |
| ATM_EGFR_TP53 | 22 | 3276 | 0.006671 | 20 | 3992 | 0.004985 | 0.354913 | 1 |
| ATM_KEAP1 | 43 | 3255 | 0.013038 | 20 | 3992 | 0.004985 | 0.000297 | 0.202156 |
| ATM_KEAP1_KRAS | 21 | 3277 | 0.006367 | 6 | 4006 | 0.001496 | 0.000724 | 0.493353 |
| ATM_KEAP1_KRAS_SMARCA4 | 7 | 3291 | 0.002122 | 3 | 4009 | 0.000748 | 0.201315 | 1 |
| ATM_KEAP1_KRAS_STK11 | 12 | 3286 | 0.003639 | 6 | 4006 | 0.001496 | 0.09464 | 1 |
| ATM_KEAP1_KRAS_TP53 | 7 | 3291 | 0.002122 | 2 | 4010 | 0.000499 | 0.088269 | 1 |
| ATM_KEAP1_NF1 | 7 | 3291 | 0.002122 | 2 | 4010 | 0.000499 | 0.088269 | 1 |
| ATM_KEAP1_NF1_SMARCA4 | 5 | 3293 | 0.001516 | 1 | 4011 | 0.000249 | 0.097192 | 1 |
| ATM_KEAP1_NF1_TP53 | 5 | 3293 | 0.001516 | 2 | 4010 | 0.000499 | 0.255537 | 1 |
| ATM_KEAP1_PTPRD | 5 | 3293 | 0.001516 | 2 | 4010 | 0.000499 | 0.255537 | 1 |
| ATM_KEAP1_SETD2 | 6 | 3292 | 0.001819 | 10 | 4002 | 0.002493 | 0.621057 | 1 |
| ATM_KEAP1_SETD2_TP53 | 6 | 3292 | 0.001819 | 6 | 4006 | 0.001496 | 0.777757 | 1 |
| ATM_KEAP1_SMARCA4 | 15 | 3283 | 0.004548 | 7 | 4005 | 0.001745 | 0.032787 | 1 |
| ATM_KEAP1_SMARCA4_STK11 | 5 | 3293 | 0.001516 | 5 | 4007 | 0.001246 | 0.762057 | 1 |
| ATM_KEAP1_SMARCA4_TP53 | 8 | 3290 | 0.002426 | 5 | 4007 | 0.001246 | 0.272151 | 1 |
| ATM_KEAP1_STK11 | 19 | 3279 | 0.005761 | 11 | 4001 | 0.002742 | 0.064202 | 1 |
| ATM_KEAP1_STK11_TP53 | 5 | 3293 | 0.001516 | 3 | 4009 | 0.000748 | 0.480506 | 1 |
| ATM_KEAP1_TP53 | 21 | 3277 | 0.006367 | 11 | 4001 | 0.002742 | 0.021027 | 1 |
| ATM_KMT2D | 30 | 3268 | 0.009096 | 28 | 3984 | 0.006979 | 0.354176 | 1 |
| ATM_KMT2D_KRAS | 9 | 3289 | 0.002729 | 10 | 4002 | 0.002493 | 1 | 1 |
| ATM_KMT2D_PTPRD | 6 | 3292 | 0.001819 | 2 | 4010 | 0.000499 | 0.151704 | 1 |
| ATM_KMT2D_PTPRD_TP53 | 6 | 3292 | 0.001819 | 1 | 4011 | 0.000249 | 0.051102 | 1 |
| ATM_KMT2D_SMARCA4 | 8 | 3290 | 0.002426 | 5 | 4007 | 0.001246 | 0.272151 | 1 |
| ATM_KMT2D_SMARCA4_TP53 | 6 | 3292 | 0.001819 | 4 | 4008 | 0.000997 | 0.362025 | 1 |
| ATM_KMT2D_TP53 | 19 | 3279 | 0.005761 | 16 | 3996 | 0.003988 | 0.308816 | 1 |
| ATM_KRAS | 109 | 3189 | 0.03305 | 136 | 3876 | 0.033898 | 0.896126 | 1 |
| ATM_KRAS_PIK3CA | 7 | 3291 | 0.002122 | 7 | 4005 | 0.001745 | 0.791309 | 1 |
| ATM_KRAS_PTPN11 | 5 | 3293 | 0.001516 | 3 | 4009 | 0.000748 | 0.480506 | 1 |
| ATM_KRAS_PTPRD | 12 | 3286 | 0.003639 | 11 | 4001 | 0.002742 | 0.533855 | 1 |
| ATM_KRAS_RBM10 | 16 | 3282 | 0.004851 | 6 | 4006 | 0.001496 | 0.010033 | 1 |
| ATM_KRAS_RBM10_TP53 | 5 | 3293 | 0.001516 | 2 | 4010 | 0.000499 | 0.255537 | 1 |
| ATM_KRAS_SETD2 | 9 | 3289 | 0.002729 | 15 | 3997 | 0.003739 | 0.54014 | 1 |
| ATM_KRAS_SMARCA4 | 13 | 3285 | 0.003942 | 10 | 4002 | 0.002493 | 0.29892 | 1 |
| ATM_KRAS_SMARCA4_STK11 | 8 | 3290 | 0.002426 | 4 | 4008 | 0.000997 | 0.15444 | 1 |
| ATM_KRAS_STK11 | 33 | 3265 | 0.010006 | 39 | 3973 | 0.009721 | 0.905876 | 1 |
| ATM_KRAS_STK11_TP53 | 8 | 3290 | 0.002426 | 7 | 4005 | 0.001745 | 0.607342 | 1 |
| ATM_KRAS_TP53 | 31 | 3267 | 0.0094 | 27 | 3985 | 0.00673 | 0.233224 | 1 |
| ATM_MET | 14 | 3284 | 0.004245 | 15 | 3997 | 0.003739 | 0.852113 | 1 |
| ATM_MET_TP53 | 6 | 3292 | 0.001819 | 8 | 4004 | 0.001994 | 1 | 1 |
| ATM_MGA | 5 | 3293 | 0.001516 | 4 | 4008 | 0.000997 | 0.739872 | 1 |
| ATM_NF1 | 24 | 3274 | 0.007277 | 26 | 3986 | 0.006481 | 0.775835 | 1 |
| ATM_NF1_PTPRD | 5 | 3293 | 0.001516 | 2 | 4010 | 0.000499 | 0.255537 | 1 |
| ATM_NF1_PTPRD_TP53 | 5 | 3293 | 0.001516 | 2 | 4010 | 0.000499 | 0.255537 | 1 |
| ATM_NF1_SMARCA4 | 10 | 3288 | 0.003032 | 7 | 4005 | 0.001745 | 0.330321 | 1 |
| ATM_NF1_SMARCA4_TP53 | 8 | 3290 | 0.002426 | 5 | 4007 | 0.001246 | 0.272151 | 1 |
| ATM_NF1_TP53 | 19 | 3279 | 0.005761 | 12 | 4000 | 0.002991 | 0.073247 | 1 |
| ATM_PIK3CA | 14 | 3284 | 0.004245 | 22 | 3990 | 0.005484 | 0.504562 | 1 |
| ATM_PIK3CA_TP53 | 8 | 3290 | 0.002426 | 15 | 3997 | 0.003739 | 0.402857 | 1 |
| ATM_PTPN11 | 7 | 3291 | 0.002122 | 8 | 4004 | 0.001994 | 1 | 1 |
| ATM_PTPRD | 24 | 3274 | 0.007277 | 15 | 3997 | 0.003739 | 0.051589 | 1 |
| ATM_PTPRD_SMARCA4 | 8 | 3290 | 0.002426 | 2 | 4010 | 0.000499 | 0.050581 | 1 |
| ATM_PTPRD_SMARCA4_TP53 | 7 | 3291 | 0.002122 | 1 | 4011 | 0.000249 | 0.026579 | 1 |
| ATM_PTPRD_TP53 | 14 | 3284 | 0.004245 | 7 | 4005 | 0.001745 | 0.050743 | 1 |
| ATM_PTPRT | 7 | 3291 | 0.002122 | 0 | 4012 | 0 | 0.003792 | 1 |
| ATM_RB1 | 17 | 3281 | 0.005155 | 11 | 4001 | 0.002742 | 0.126871 | 1 |
| ATM_RB1_TP53 | 13 | 3285 | 0.003942 | 5 | 4007 | 0.001246 | 0.030231 | 1 |
| ATM_RBM10 | 22 | 3276 | 0.006671 | 11 | 4001 | 0.002742 | 0.01401 | 1 |
| ATM_RBM10_TP53 | 9 | 3289 | 0.002729 | 6 | 4006 | 0.001496 | 0.302316 | 1 |
| ATM_SETD2 | 19 | 3279 | 0.005761 | 30 | 3982 | 0.007478 | 0.391294 | 1 |
| ATM_SETD2_TP53 | 9 | 3289 | 0.002729 | 13 | 3999 | 0.00324 | 0.830965 | 1 |
| ATM_SMARCA4 | 33 | 3265 | 0.010006 | 22 | 3990 | 0.005484 | 0.029331 | 1 |
| ATM_SMARCA4_STK11 | 11 | 3287 | 0.003335 | 9 | 4003 | 0.002243 | 0.379793 | 1 |
| ATM_SMARCA4_TP53 | 19 | 3279 | 0.005761 | 13 | 3999 | 0.00324 | 0.111827 | 1 |
| ATM_STK11 | 49 | 3249 | 0.014857 | 62 | 3950 | 0.015454 | 0.848285 | 1 |
| ATM_STK11_TP53 | 18 | 3280 | 0.005458 | 14 | 3998 | 0.00349 | 0.21699 | 1 |
| ATM_TP53 | 103 | 3195 | 0.031231 | 96 | 3916 | 0.023928 | 0.060386 | 1 |
| BRAF | 168 | 3130 | 0.05094 | 205 | 3807 | 0.051097 | 1 | 1 |
| BRAF_CDKN2A | 12 | 3286 | 0.003639 | 11 | 4001 | 0.002742 | 0.533855 | 1 |
| BRAF_CDKN2A_TP53 | 8 | 3290 | 0.002426 | 8 | 4004 | 0.001994 | 0.803112 | 1 |
| BRAF_CTNNB1 | 7 | 3291 | 0.002122 | 9 | 4003 | 0.002243 | 1 | 1 |
| BRAF_CTNNB1_TP53 | 5 | 3293 | 0.001516 | 3 | 4009 | 0.000748 | 0.480506 | 1 |
| BRAF_EGFR | 16 | 3282 | 0.004851 | 12 | 4000 | 0.002991 | 0.253588 | 1 |
| BRAF_EGFR_TP53 | 12 | 3286 | 0.003639 | 6 | 4006 | 0.001496 | 0.09464 | 1 |
| BRAF_KEAP1 | 24 | 3274 | 0.007277 | 16 | 3996 | 0.003988 | 0.078405 | 1 |
| BRAF_KEAP1_KRAS | 8 | 3290 | 0.002426 | 5 | 4007 | 0.001246 | 0.272151 | 1 |
| BRAF_KEAP1_KRAS_STK11 | 5 | 3293 | 0.001516 | 1 | 4011 | 0.000249 | 0.097192 | 1 |
| BRAF_KEAP1_NF1 | 7 | 3291 | 0.002122 | 2 | 4010 | 0.000499 | 0.088269 | 1 |
| BRAF_KEAP1_NF1_TP53 | 5 | 3293 | 0.001516 | 1 | 4011 | 0.000249 | 0.097192 | 1 |
| BRAF_KEAP1_SMARCA4 | 5 | 3293 | 0.001516 | 4 | 4008 | 0.000997 | 0.739872 | 1 |
| BRAF_KEAP1_STK11 | 14 | 3284 | 0.004245 | 5 | 4007 | 0.001246 | 0.018591 | 1 |
| BRAF_KEAP1_STK11_TP53 | 6 | 3292 | 0.001819 | 1 | 4011 | 0.000249 | 0.051102 | 1 |
| BRAF_KEAP1_TP53 | 14 | 3284 | 0.004245 | 9 | 4003 | 0.002243 | 0.144789 | 1 |
| BRAF_KMT2D | 11 | 3287 | 0.003335 | 12 | 4000 | 0.002991 | 0.835861 | 1 |
| BRAF_KMT2D_TP53 | 6 | 3292 | 0.001819 | 8 | 4004 | 0.001994 | 1 | 1 |
| BRAF_KRAS | 32 | 3266 | 0.009703 | 37 | 3975 | 0.009222 | 0.903393 | 1 |
| BRAF_KRAS_PTPRD | 5 | 3293 | 0.001516 | 1 | 4011 | 0.000249 | 0.097192 | 1 |
| BRAF_KRAS_STK11 | 9 | 3289 | 0.002729 | 7 | 4005 | 0.001745 | 0.453469 | 1 |
| BRAF_KRAS_TP53 | 17 | 3281 | 0.005155 | 9 | 4003 | 0.002243 | 0.047278 | 1 |
| BRAF_MET | 6 | 3292 | 0.001819 | 7 | 4005 | 0.001745 | 1 | 1 |
| BRAF_MET_TP53 | 5 | 3293 | 0.001516 | 3 | 4009 | 0.000748 | 0.480506 | 1 |
| BRAF_NF1 | 18 | 3280 | 0.005458 | 8 | 4004 | 0.001994 | 0.016765 | 1 |
| BRAF_NF1_SMARCA4 | 5 | 3293 | 0.001516 | 4 | 4008 | 0.000997 | 0.739872 | 1 |
| BRAF_NF1_SMARCA4_TP53 | 5 | 3293 | 0.001516 | 4 | 4008 | 0.000997 | 0.739872 | 1 |
| BRAF_NF1_TP53 | 15 | 3283 | 0.004548 | 5 | 4007 | 0.001246 | 0.011309 | 1 |
| BRAF_PIK3CA | 10 | 3288 | 0.003032 | 13 | 3999 | 0.00324 | 1 | 1 |
| BRAF_PIK3CA_TP53 | 7 | 3291 | 0.002122 | 8 | 4004 | 0.001994 | 1 | 1 |
| BRAF_PTPRD | 14 | 3284 | 0.004245 | 5 | 4007 | 0.001246 | 0.018591 | 1 |
| BRAF_PTPRD_SMARCA4 | 5 | 3293 | 0.001516 | 1 | 4011 | 0.000249 | 0.097192 | 1 |
| BRAF_PTPRD_TP53 | 10 | 3288 | 0.003032 | 3 | 4009 | 0.000748 | 0.025437 | 1 |
| BRAF_RB1 | 9 | 3289 | 0.002729 | 6 | 4006 | 0.001496 | 0.302316 | 1 |
| BRAF_RB1_TP53 | 6 | 3292 | 0.001819 | 4 | 4008 | 0.000997 | 0.362025 | 1 |
| BRAF_RBM10 | 5 | 3293 | 0.001516 | 2 | 4010 | 0.000499 | 0.255537 | 1 |
| BRAF_SETD2 | 27 | 3271 | 0.008187 | 20 | 3992 | 0.004985 | 0.105359 | 1 |
| BRAF_SETD2_TP53 | 13 | 3285 | 0.003942 | 6 | 4006 | 0.001496 | 0.062031 | 1 |
| BRAF_SMARCA4 | 16 | 3282 | 0.004851 | 9 | 4003 | 0.002243 | 0.069676 | 1 |
| BRAF_SMARCA4_TP53 | 12 | 3286 | 0.003639 | 5 | 4007 | 0.001246 | 0.048542 | 1 |
| BRAF_STK11 | 24 | 3274 | 0.007277 | 30 | 3982 | 0.007478 | 1 | 1 |
| BRAF_STK11_TP53 | 11 | 3287 | 0.003335 | 10 | 4002 | 0.002493 | 0.518426 | 1 |
| BRAF_TP53 | 88 | 3210 | 0.026683 | 72 | 3940 | 0.017946 | 0.012604 | 1 |
| CDKN2A | 204 | 3094 | 0.061856 | 224 | 3788 | 0.055833 | 0.293172 | 1 |
| CDKN2A_CTNNB1 | 11 | 3287 | 0.003335 | 3 | 4009 | 0.000748 | 0.014426 | 1 |
| CDKN2A_CTNNB1_TP53 | 10 | 3288 | 0.003032 | 3 | 4009 | 0.000748 | 0.025437 | 1 |
| CDKN2A_EGFR | 25 | 3273 | 0.00758 | 30 | 3982 | 0.007478 | 1 | 1 |
| CDKN2A_EGFR_TP53 | 19 | 3279 | 0.005761 | 20 | 3992 | 0.004985 | 0.747446 | 1 |
| CDKN2A_KEAP1 | 36 | 3262 | 0.010916 | 25 | 3987 | 0.006231 | 0.037855 | 1 |
| CDKN2A_KEAP1_KMT2D | 9 | 3289 | 0.002729 | 8 | 4004 | 0.001994 | 0.627537 | 1 |
| CDKN2A_KEAP1_KMT2D_NF1 | 5 | 3293 | 0.001516 | 2 | 4010 | 0.000499 | 0.255537 | 1 |
| CDKN2A_KEAP1_KMT2D_NF1_TP53 | 5 | 3293 | 0.001516 | 1 | 4011 | 0.000249 | 0.097192 | 1 |
| CDKN2A_KEAP1_KMT2D_TP53 | 9 | 3289 | 0.002729 | 6 | 4006 | 0.001496 | 0.302316 | 1 |
| CDKN2A_KEAP1_NF1 | 14 | 3284 | 0.004245 | 3 | 4009 | 0.000748 | 0.002455 | 1 |
| CDKN2A_KEAP1_NF1_SETD2 | 5 | 3293 | 0.001516 | 1 | 4011 | 0.000249 | 0.097192 | 1 |
| CDKN2A_KEAP1_NF1_SETD2_TP53 | 5 | 3293 | 0.001516 | 1 | 4011 | 0.000249 | 0.097192 | 1 |
| CDKN2A_KEAP1_NF1_TP53 | 13 | 3285 | 0.003942 | 2 | 4010 | 0.000499 | 0.001251 | 0.852126 |
| CDKN2A_KEAP1_PIK3CA | 7 | 3291 | 0.002122 | 3 | 4009 | 0.000748 | 0.201315 | 1 |
| CDKN2A_KEAP1_PIK3CA_TP53 | 7 | 3291 | 0.002122 | 3 | 4009 | 0.000748 | 0.201315 | 1 |
| CDKN2A_KEAP1_PTPRD | 9 | 3289 | 0.002729 | 3 | 4009 | 0.000748 | 0.044183 | 1 |
| CDKN2A_KEAP1_PTPRD_TP53 | 9 | 3289 | 0.002729 | 3 | 4009 | 0.000748 | 0.044183 | 1 |
| CDKN2A_KEAP1_RBM10 | 5 | 3293 | 0.001516 | 2 | 4010 | 0.000499 | 0.255537 | 1 |
| CDKN2A_KEAP1_RBM10_TP53 | 5 | 3293 | 0.001516 | 0 | 4012 | 0 | 0.018661 | 1 |
| CDKN2A_KEAP1_SETD2 | 8 | 3290 | 0.002426 | 3 | 4009 | 0.000748 | 0.075396 | 1 |
| CDKN2A_KEAP1_SETD2_TP53 | 8 | 3290 | 0.002426 | 2 | 4010 | 0.000499 | 0.050581 | 1 |
| CDKN2A_KEAP1_SMARCA4 | 8 | 3290 | 0.002426 | 9 | 4003 | 0.002243 | 1 | 1 |
| CDKN2A_KEAP1_SMARCA4_TP53 | 8 | 3290 | 0.002426 | 5 | 4007 | 0.001246 | 0.272151 | 1 |
| CDKN2A_KEAP1_STK11 | 9 | 3289 | 0.002729 | 7 | 4005 | 0.001745 | 0.453469 | 1 |
| CDKN2A_KEAP1_STK11_TP53 | 7 | 3291 | 0.002122 | 2 | 4010 | 0.000499 | 0.088269 | 1 |
| CDKN2A_KEAP1_TP53 | 32 | 3266 | 0.009703 | 19 | 3993 | 0.004736 | 0.015544 | 1 |
| CDKN2A_KMT2D | 19 | 3279 | 0.005761 | 32 | 3980 | 0.007976 | 0.322991 | 1 |
| CDKN2A_KMT2D_NF1 | 7 | 3291 | 0.002122 | 6 | 4006 | 0.001496 | 0.584537 | 1 |
| CDKN2A_KMT2D_NF1_TP53 | 7 | 3291 | 0.002122 | 4 | 4008 | 0.000997 | 0.239779 | 1 |
| CDKN2A_KMT2D_PIK3CA | 5 | 3293 | 0.001516 | 5 | 4007 | 0.001246 | 0.762057 | 1 |
| CDKN2A_KMT2D_PIK3CA_TP53 | 5 | 3293 | 0.001516 | 5 | 4007 | 0.001246 | 0.762057 | 1 |
| CDKN2A_KMT2D_SETD2 | 5 | 3293 | 0.001516 | 2 | 4010 | 0.000499 | 0.255537 | 1 |
| CDKN2A_KMT2D_SETD2_TP53 | 5 | 3293 | 0.001516 | 2 | 4010 | 0.000499 | 0.255537 | 1 |
| CDKN2A_KMT2D_SMARCA4 | 5 | 3293 | 0.001516 | 4 | 4008 | 0.000997 | 0.739872 | 1 |
| CDKN2A_KMT2D_SMARCA4_TP53 | 5 | 3293 | 0.001516 | 3 | 4009 | 0.000748 | 0.480506 | 1 |
| CDKN2A_KMT2D_TP53 | 19 | 3279 | 0.005761 | 28 | 3984 | 0.006979 | 0.558804 | 1 |
| CDKN2A_KRAS | 44 | 3254 | 0.013341 | 73 | 3939 | 0.018195 | 0.111303 | 1 |
| CDKN2A_KRAS_PTPRD | 5 | 3293 | 0.001516 | 1 | 4011 | 0.000249 | 0.097192 | 1 |
| CDKN2A_KRAS_SMARCA4 | 7 | 3291 | 0.002122 | 3 | 4009 | 0.000748 | 0.201315 | 1 |
| CDKN2A_KRAS_SMARCA4_TP53 | 5 | 3293 | 0.001516 | 0 | 4012 | 0 | 0.018661 | 1 |
| CDKN2A_KRAS_STK11 | 8 | 3290 | 0.002426 | 12 | 4000 | 0.002991 | 0.822712 | 1 |
| CDKN2A_KRAS_STK11_TP53 | 5 | 3293 | 0.001516 | 2 | 4010 | 0.000499 | 0.255537 | 1 |
| CDKN2A_KRAS_TP53 | 32 | 3266 | 0.009703 | 42 | 3970 | 0.010469 | 0.814677 | 1 |
| CDKN2A_MET | 8 | 3290 | 0.002426 | 15 | 3997 | 0.003739 | 0.402857 | 1 |
| CDKN2A_MET_TP53 | 5 | 3293 | 0.001516 | 9 | 4003 | 0.002243 | 0.595265 | 1 |
| CDKN2A_MGA | 7 | 3291 | 0.002122 | 3 | 4009 | 0.000748 | 0.201315 | 1 |
| CDKN2A_MGA_TP53 | 6 | 3292 | 0.001819 | 3 | 4009 | 0.000748 | 0.315191 | 1 |
| CDKN2A_NF1 | 29 | 3269 | 0.008793 | 15 | 3997 | 0.003739 | 0.006027 | 1 |
| CDKN2A_NF1_PTPRD | 6 | 3292 | 0.001819 | 0 | 4012 | 0 | 0.008412 | 1 |
| CDKN2A_NF1_PTPRD_TP53 | 6 | 3292 | 0.001819 | 0 | 4012 | 0 | 0.008412 | 1 |
| CDKN2A_NF1_RBM10 | 5 | 3293 | 0.001516 | 0 | 4012 | 0 | 0.018661 | 1 |
| CDKN2A_NF1_RBM10_TP53 | 5 | 3293 | 0.001516 | 0 | 4012 | 0 | 0.018661 | 1 |
| CDKN2A_NF1_SETD2 | 6 | 3292 | 0.001819 | 3 | 4009 | 0.000748 | 0.315191 | 1 |
| CDKN2A_NF1_SETD2_TP53 | 6 | 3292 | 0.001819 | 3 | 4009 | 0.000748 | 0.315191 | 1 |
| CDKN2A_NF1_SMARCA4 | 10 | 3288 | 0.003032 | 3 | 4009 | 0.000748 | 0.025437 | 1 |
| CDKN2A_NF1_SMARCA4_TP53 | 9 | 3289 | 0.002729 | 2 | 4010 | 0.000499 | 0.028651 | 1 |
| CDKN2A_NF1_TP53 | 26 | 3272 | 0.007884 | 11 | 4001 | 0.002742 | 0.002501 | 1 |
| CDKN2A_PIK3CA | 14 | 3284 | 0.004245 | 16 | 3996 | 0.003988 | 0.856769 | 1 |
| CDKN2A_PIK3CA_TP53 | 10 | 3288 | 0.003032 | 15 | 3997 | 0.003739 | 0.68975 | 1 |
| CDKN2A_PTPRD | 21 | 3277 | 0.006367 | 10 | 4002 | 0.002493 | 0.017235 | 1 |
| CDKN2A_PTPRD_TP53 | 18 | 3280 | 0.005458 | 10 | 4002 | 0.002493 | 0.055465 | 1 |
| CDKN2A_PTPRT | 8 | 3290 | 0.002426 | 0 | 4012 | 0 | 0.001709 | 1 |
| CDKN2A_PTPRT_TP53 | 7 | 3291 | 0.002122 | 0 | 4012 | 0 | 0.003792 | 1 |
| CDKN2A_RB1 | 7 | 3291 | 0.002122 | 5 | 4007 | 0.001246 | 0.395739 | 1 |
| CDKN2A_RB1_TP53 | 7 | 3291 | 0.002122 | 4 | 4008 | 0.000997 | 0.239779 | 1 |
| CDKN2A_RBM10 | 13 | 3285 | 0.003942 | 7 | 4005 | 0.001745 | 0.11297 | 1 |
| CDKN2A_RBM10_TP53 | 12 | 3286 | 0.003639 | 4 | 4008 | 0.000997 | 0.021618 | 1 |
| CDKN2A_SETD2 | 13 | 3285 | 0.003942 | 9 | 4003 | 0.002243 | 0.20357 | 1 |
| CDKN2A_SETD2_TP53 | 12 | 3286 | 0.003639 | 5 | 4007 | 0.001246 | 0.048542 | 1 |
| CDKN2A_SMARCA4 | 34 | 3264 | 0.010309 | 16 | 3996 | 0.003988 | 0.001461 | 0.994691 |
| CDKN2A_SMARCA4_STK11 | 7 | 3291 | 0.002122 | 7 | 4005 | 0.001745 | 0.791309 | 1 |
| CDKN2A_SMARCA4_STK11_TP53 | 6 | 3292 | 0.001819 | 4 | 4008 | 0.000997 | 0.362025 | 1 |
| CDKN2A_SMARCA4_TP53 | 30 | 3268 | 0.009096 | 11 | 4001 | 0.002742 | 0.000394 | 0.268375 |
| CDKN2A_STK11 | 29 | 3269 | 0.008793 | 28 | 3984 | 0.006979 | 0.423493 | 1 |
| CDKN2A_STK11_TP53 | 21 | 3277 | 0.006367 | 14 | 3998 | 0.00349 | 0.088738 | 1 |
| CDKN2A_TP53 | 154 | 3144 | 0.046695 | 161 | 3851 | 0.04013 | 0.183089 | 1 |
| CTNNB1 | 117 | 3181 | 0.035476 | 111 | 3901 | 0.027667 | 0.05845 | 1 |
| CTNNB1_EGFR | 60 | 3238 | 0.018193 | 38 | 3974 | 0.009472 | 0.001446 | 0.984927 |
| CTNNB1_EGFR_PIK3CA | 9 | 3289 | 0.002729 | 2 | 4010 | 0.000499 | 0.028651 | 1 |
| CTNNB1_EGFR_TP53 | 26 | 3272 | 0.007884 | 11 | 4001 | 0.002742 | 0.002501 | 1 |
| CTNNB1_KEAP1 | 8 | 3290 | 0.002426 | 12 | 4000 | 0.002991 | 0.822712 | 1 |
| CTNNB1_KEAP1_TP53 | 7 | 3291 | 0.002122 | 7 | 4005 | 0.001745 | 0.791309 | 1 |
| CTNNB1_KMT2D | 5 | 3293 | 0.001516 | 9 | 4003 | 0.002243 | 0.595265 | 1 |
| CTNNB1_KRAS | 16 | 3282 | 0.004851 | 30 | 3982 | 0.007478 | 0.18177 | 1 |
| CTNNB1_KRAS_TP53 | 8 | 3290 | 0.002426 | 9 | 4003 | 0.002243 | 1 | 1 |
| CTNNB1_NF1 | 6 | 3292 | 0.001819 | 3 | 4009 | 0.000748 | 0.315191 | 1 |
| CTNNB1_NF1_TP53 | 5 | 3293 | 0.001516 | 1 | 4011 | 0.000249 | 0.097192 | 1 |
| CTNNB1_PIK3CA | 15 | 3283 | 0.004548 | 11 | 4001 | 0.002742 | 0.23713 | 1 |
| CTNNB1_PIK3CA_TP53 | 5 | 3293 | 0.001516 | 6 | 4006 | 0.001496 | 1 | 1 |
| CTNNB1_PTPRD | 7 | 3291 | 0.002122 | 3 | 4009 | 0.000748 | 0.201315 | 1 |
| CTNNB1_PTPRD_TP53 | 7 | 3291 | 0.002122 | 2 | 4010 | 0.000499 | 0.088269 | 1 |
| CTNNB1_SETD2 | 6 | 3292 | 0.001819 | 6 | 4006 | 0.001496 | 0.777757 | 1 |
| CTNNB1_SMARCA4 | 6 | 3292 | 0.001819 | 4 | 4008 | 0.000997 | 0.362025 | 1 |
| CTNNB1_STK11 | 6 | 3292 | 0.001819 | 14 | 3998 | 0.00349 | 0.186431 | 1 |
| CTNNB1_TP53 | 56 | 3242 | 0.01698 | 37 | 3975 | 0.009222 | 0.004381 | 1 |
| EGFR | 748 | 2550 | 0.226804 | 700 | 3312 | 0.174477 | 2.86E-08 | 1.95E-05 |
| EGFR_KEAP1 | 15 | 3283 | 0.004548 | 16 | 3996 | 0.003988 | 0.721522 | 1 |
| EGFR_KEAP1_PTPRD | 7 | 3291 | 0.002122 | 0 | 4012 | 0 | 0.003792 | 1 |
| EGFR_KEAP1_PTPRD_TP53 | 6 | 3292 | 0.001819 | 0 | 4012 | 0 | 0.008412 | 1 |
| EGFR_KEAP1_TP53 | 9 | 3289 | 0.002729 | 13 | 3999 | 0.00324 | 0.830965 | 1 |
| EGFR_KMT2D | 22 | 3276 | 0.006671 | 25 | 3987 | 0.006231 | 0.883419 | 1 |
| EGFR_KMT2D_TP53 | 15 | 3283 | 0.004548 | 18 | 3994 | 0.004487 | 1 | 1 |
| EGFR_KRAS | 19 | 3279 | 0.005761 | 33 | 3979 | 0.008225 | 0.26314 | 1 |
| EGFR_KRAS_RB1 | 5 | 3293 | 0.001516 | 6 | 4006 | 0.001496 | 1 | 1 |
| EGFR_KRAS_RB1_TP53 | 5 | 3293 | 0.001516 | 5 | 4007 | 0.001246 | 0.762057 | 1 |
| EGFR_KRAS_TP53 | 10 | 3288 | 0.003032 | 18 | 3994 | 0.004487 | 0.347198 | 1 |
| EGFR_MET | 14 | 3284 | 0.004245 | 19 | 3993 | 0.004736 | 0.861385 | 1 |
| EGFR_MET_TP53 | 10 | 3288 | 0.003032 | 12 | 4000 | 0.002991 | 1 | 1 |
| EGFR_MGA | 9 | 3289 | 0.002729 | 13 | 3999 | 0.00324 | 0.830965 | 1 |
| EGFR_MGA_TP53 | 7 | 3291 | 0.002122 | 6 | 4006 | 0.001496 | 0.584537 | 1 |
| EGFR_NF1 | 25 | 3273 | 0.00758 | 22 | 3990 | 0.005484 | 0.303907 | 1 |
| EGFR_NF1_RB1 | 5 | 3293 | 0.001516 | 1 | 4011 | 0.000249 | 0.097192 | 1 |
| EGFR_NF1_TP53 | 18 | 3280 | 0.005458 | 15 | 3997 | 0.003739 | 0.296482 | 1 |
| EGFR_PIK3CA | 69 | 3229 | 0.020922 | 39 | 3973 | 0.009721 | 8.63E-05 | 0.058749 |
| EGFR_PIK3CA_RB1 | 9 | 3289 | 0.002729 | 4 | 4008 | 0.000997 | 0.09713 | 1 |
| EGFR_PIK3CA_RB1_TP53 | 8 | 3290 | 0.002426 | 4 | 4008 | 0.000997 | 0.15444 | 1 |
| EGFR_PIK3CA_SETD2 | 5 | 3293 | 0.001516 | 3 | 4009 | 0.000748 | 0.480506 | 1 |
| EGFR_PIK3CA_TP53 | 39 | 3259 | 0.011825 | 19 | 3993 | 0.004736 | 0.000806 | 0.548894 |
| EGFR_PTPN11 | 6 | 3292 | 0.001819 | 7 | 4005 | 0.001745 | 1 | 1 |
| EGFR_PTPN11_TP53 | 5 | 3293 | 0.001516 | 3 | 4009 | 0.000748 | 0.480506 | 1 |
| EGFR_PTPRD | 26 | 3272 | 0.007884 | 14 | 3998 | 0.00349 | 0.015807 | 1 |
| EGFR_PTPRD_SMARCA4 | 5 | 3293 | 0.001516 | 1 | 4011 | 0.000249 | 0.097192 | 1 |
| EGFR_PTPRD_TP53 | 22 | 3276 | 0.006671 | 12 | 4000 | 0.002991 | 0.024488 | 1 |
| EGFR_PTPRT | 8 | 3290 | 0.002426 | 0 | 4012 | 0 | 0.001709 | 1 |
| EGFR_RB1 | 70 | 3228 | 0.021225 | 29 | 3983 | 0.007228 | 3.57E-07 | 0.000243 |
| EGFR_RB1_TP53 | 60 | 3238 | 0.018193 | 24 | 3988 | 0.005982 | 1.27E-06 | 0.000862 |
| EGFR_RBM10 | 27 | 3271 | 0.008187 | 15 | 3997 | 0.003739 | 0.018574 | 1 |
| EGFR_RBM10_TP53 | 13 | 3285 | 0.003942 | 9 | 4003 | 0.002243 | 0.20357 | 1 |
| EGFR_SETD2 | 24 | 3274 | 0.007277 | 24 | 3988 | 0.005982 | 0.561186 | 1 |
| EGFR_SETD2_TP53 | 19 | 3279 | 0.005761 | 12 | 4000 | 0.002991 | 0.073247 | 1 |
| EGFR_SMARCA4 | 31 | 3267 | 0.0094 | 17 | 3995 | 0.004237 | 0.008254 | 1 |
| EGFR_SMARCA4_TP53 | 27 | 3271 | 0.008187 | 12 | 4000 | 0.002991 | 0.003197 | 1 |
| EGFR_STK11 | 17 | 3281 | 0.005155 | 20 | 3992 | 0.004985 | 1 | 1 |
| EGFR_STK11_TP53 | 14 | 3284 | 0.004245 | 11 | 4001 | 0.002742 | 0.316501 | 1 |
| EGFR_TP53 | 463 | 2835 | 0.140388 | 319 | 3693 | 0.079511 | 7.55E-17 | 5.14E-14 |
| KEAP1 | 313 | 2985 | 0.094906 | 242 | 3770 | 0.060319 | 3.36E-08 | 2.29E-05 |
| KEAP1_KMT2D | 40 | 3258 | 0.012129 | 38 | 3974 | 0.009472 | 0.303543 | 1 |
| KEAP1_KMT2D_KRAS | 13 | 3285 | 0.003942 | 9 | 4003 | 0.002243 | 0.20357 | 1 |
| KEAP1_KMT2D_KRAS_STK11 | 10 | 3288 | 0.003032 | 5 | 4007 | 0.001246 | 0.119521 | 1 |
| KEAP1_KMT2D_KRAS_TP53 | 6 | 3292 | 0.001819 | 1 | 4011 | 0.000249 | 0.051102 | 1 |
| KEAP1_KMT2D_NF1 | 8 | 3290 | 0.002426 | 6 | 4006 | 0.001496 | 0.426098 | 1 |
| KEAP1_KMT2D_NF1_TP53 | 8 | 3290 | 0.002426 | 5 | 4007 | 0.001246 | 0.272151 | 1 |
| KEAP1_KMT2D_PIK3CA | 7 | 3291 | 0.002122 | 6 | 4006 | 0.001496 | 0.584537 | 1 |
| KEAP1_KMT2D_PIK3CA_TP53 | 6 | 3292 | 0.001819 | 5 | 4007 | 0.001246 | 0.558601 | 1 |
| KEAP1_KMT2D_PTPRD | 8 | 3290 | 0.002426 | 7 | 4005 | 0.001745 | 0.607342 | 1 |
| KEAP1_KMT2D_PTPRD_STK11 | 5 | 3293 | 0.001516 | 4 | 4008 | 0.000997 | 0.739872 | 1 |
| KEAP1_KMT2D_PTPRD_TP53 | 5 | 3293 | 0.001516 | 6 | 4006 | 0.001496 | 1 | 1 |
| KEAP1_KMT2D_SETD2 | 8 | 3290 | 0.002426 | 6 | 4006 | 0.001496 | 0.426098 | 1 |
| KEAP1_KMT2D_SETD2_TP53 | 6 | 3292 | 0.001819 | 4 | 4008 | 0.000997 | 0.362025 | 1 |
| KEAP1_KMT2D_SMARCA4 | 9 | 3289 | 0.002729 | 9 | 4003 | 0.002243 | 0.813495 | 1 |
| KEAP1_KMT2D_SMARCA4_TP53 | 6 | 3292 | 0.001819 | 5 | 4007 | 0.001246 | 0.558601 | 1 |
| KEAP1_KMT2D_STK11 | 16 | 3282 | 0.004851 | 17 | 3995 | 0.004237 | 0.728293 | 1 |
| KEAP1_KMT2D_STK11_TP53 | 9 | 3289 | 0.002729 | 8 | 4004 | 0.001994 | 0.627537 | 1 |
| KEAP1_KMT2D_TP53 | 30 | 3268 | 0.009096 | 24 | 3988 | 0.005982 | 0.132141 | 1 |
| KEAP1_KRAS | 128 | 3170 | 0.038811 | 79 | 3933 | 0.019691 | 1.18E-06 | 0.000807 |
| KEAP1_KRAS_NF1 | 8 | 3290 | 0.002426 | 3 | 4009 | 0.000748 | 0.075396 | 1 |
| KEAP1_KRAS_NF1_STK11 | 5 | 3293 | 0.001516 | 1 | 4011 | 0.000249 | 0.097192 | 1 |
| KEAP1_KRAS_PTPRD | 21 | 3277 | 0.006367 | 3 | 4009 | 0.000748 | 3.14E-05 | 0.021359 |
| KEAP1_KRAS_PTPRD_STK11 | 11 | 3287 | 0.003335 | 0 | 4012 | 0 | 0.000156 | 0.106373 |
| KEAP1_KRAS_RBM10 | 11 | 3287 | 0.003335 | 5 | 4007 | 0.001246 | 0.076815 | 1 |
| KEAP1_KRAS_RBM10_SMARCA4 | 5 | 3293 | 0.001516 | 3 | 4009 | 0.000748 | 0.480506 | 1 |
| KEAP1_KRAS_RBM10_STK11 | 7 | 3291 | 0.002122 | 3 | 4009 | 0.000748 | 0.201315 | 1 |
| KEAP1_KRAS_SETD2 | 7 | 3291 | 0.002122 | 7 | 4005 | 0.001745 | 0.791309 | 1 |
| KEAP1_KRAS_SETD2_STK11 | 6 | 3292 | 0.001819 | 4 | 4008 | 0.000997 | 0.362025 | 1 |
| KEAP1_KRAS_SMARCA4 | 25 | 3273 | 0.00758 | 22 | 3990 | 0.005484 | 0.303907 | 1 |
| KEAP1_KRAS_SMARCA4_STK11 | 19 | 3279 | 0.005761 | 20 | 3992 | 0.004985 | 0.747446 | 1 |
| KEAP1_KRAS_SMARCA4_TP53 | 5 | 3293 | 0.001516 | 2 | 4010 | 0.000499 | 0.255537 | 1 |
| KEAP1_KRAS_STK11 | 86 | 3212 | 0.026076 | 44 | 3968 | 0.010967 | 1.21E-06 | 0.000826 |
| KEAP1_KRAS_STK11_TP53 | 17 | 3281 | 0.005155 | 5 | 4007 | 0.001246 | 0.004075 | 1 |
| KEAP1_KRAS_TP53 | 33 | 3265 | 0.010006 | 19 | 3993 | 0.004736 | 0.011098 | 1 |
| KEAP1_MET | 12 | 3286 | 0.003639 | 17 | 3995 | 0.004237 | 0.713207 | 1 |
| KEAP1_MET_TP53 | 7 | 3291 | 0.002122 | 12 | 4000 | 0.002991 | 0.499597 | 1 |
| KEAP1_MGA | 13 | 3285 | 0.003942 | 6 | 4006 | 0.001496 | 0.062031 | 1 |
| KEAP1_MGA_STK11 | 8 | 3290 | 0.002426 | 3 | 4009 | 0.000748 | 0.075396 | 1 |
| KEAP1_MGA_STK11_TP53 | 6 | 3292 | 0.001819 | 2 | 4010 | 0.000499 | 0.151704 | 1 |
| KEAP1_MGA_TP53 | 11 | 3287 | 0.003335 | 4 | 4008 | 0.000997 | 0.036242 | 1 |
| KEAP1_NF1 | 53 | 3245 | 0.01607 | 23 | 3989 | 0.005733 | 2.12E-05 | 0.014425 |
| KEAP1_NF1_PIK3CA | 7 | 3291 | 0.002122 | 4 | 4008 | 0.000997 | 0.239779 | 1 |
| KEAP1_NF1_PIK3CA_TP53 | 7 | 3291 | 0.002122 | 4 | 4008 | 0.000997 | 0.239779 | 1 |
| KEAP1_NF1_PTPRD | 11 | 3287 | 0.003335 | 2 | 4010 | 0.000499 | 0.004621 | 1 |
| KEAP1_NF1_PTPRD_TP53 | 9 | 3289 | 0.002729 | 1 | 4011 | 0.000249 | 0.007044 | 1 |
| KEAP1_NF1_RBM10 | 5 | 3293 | 0.001516 | 1 | 4011 | 0.000249 | 0.097192 | 1 |
| KEAP1_NF1_SETD2 | 6 | 3292 | 0.001819 | 4 | 4008 | 0.000997 | 0.362025 | 1 |
| KEAP1_NF1_SETD2_TP53 | 6 | 3292 | 0.001819 | 4 | 4008 | 0.000997 | 0.362025 | 1 |
| KEAP1_NF1_SMARCA4 | 12 | 3286 | 0.003639 | 6 | 4006 | 0.001496 | 0.09464 | 1 |
| KEAP1_NF1_SMARCA4_STK11 | 5 | 3293 | 0.001516 | 1 | 4011 | 0.000249 | 0.097192 | 1 |
| KEAP1_NF1_SMARCA4_TP53 | 8 | 3290 | 0.002426 | 4 | 4008 | 0.000997 | 0.15444 | 1 |
| KEAP1_NF1_STK11 | 20 | 3278 | 0.006064 | 7 | 4005 | 0.001745 | 0.003054 | 1 |
| KEAP1_NF1_STK11_TP53 | 12 | 3286 | 0.003639 | 5 | 4007 | 0.001246 | 0.048542 | 1 |
| KEAP1_NF1_TP53 | 37 | 3261 | 0.011219 | 16 | 3996 | 0.003988 | 0.000419 | 0.285585 |
| KEAP1_PIK3CA | 20 | 3278 | 0.006064 | 18 | 3994 | 0.004487 | 0.414462 | 1 |
| KEAP1_PIK3CA_PTPRD | 6 | 3292 | 0.001819 | 1 | 4011 | 0.000249 | 0.051102 | 1 |
| KEAP1_PIK3CA_PTPRD_TP53 | 6 | 3292 | 0.001819 | 0 | 4012 | 0 | 0.008412 | 1 |
| KEAP1_PIK3CA_STK11 | 8 | 3290 | 0.002426 | 6 | 4006 | 0.001496 | 0.426098 | 1 |
| KEAP1_PIK3CA_STK11_TP53 | 6 | 3292 | 0.001819 | 3 | 4009 | 0.000748 | 0.315191 | 1 |
| KEAP1_PIK3CA_TP53 | 17 | 3281 | 0.005155 | 13 | 3999 | 0.00324 | 0.269746 | 1 |
| KEAP1_PTPN11 | 5 | 3293 | 0.001516 | 7 | 4005 | 0.001745 | 1 | 1 |
| KEAP1_PTPRD | 54 | 3244 | 0.016374 | 20 | 3992 | 0.004985 | 1.49E-06 | 0.001013 |
| KEAP1_PTPRD_PTPRT | 7 | 3291 | 0.002122 | 0 | 4012 | 0 | 0.003792 | 1 |
| KEAP1_PTPRD_PTPRT_TP53 | 5 | 3293 | 0.001516 | 0 | 4012 | 0 | 0.018661 | 1 |
| KEAP1_PTPRD_SETD2 | 6 | 3292 | 0.001819 | 3 | 4009 | 0.000748 | 0.315191 | 1 |
| KEAP1_PTPRD_SETD2_TP53 | 5 | 3293 | 0.001516 | 0 | 4012 | 0 | 0.018661 | 1 |
| KEAP1_PTPRD_SMARCA4 | 9 | 3289 | 0.002729 | 4 | 4008 | 0.000997 | 0.09713 | 1 |
| KEAP1_PTPRD_SMARCA4_STK11 | 5 | 3293 | 0.001516 | 4 | 4008 | 0.000997 | 0.739872 | 1 |
| KEAP1_PTPRD_STK11 | 18 | 3280 | 0.005458 | 11 | 4001 | 0.002742 | 0.090828 | 1 |
| KEAP1_PTPRD_STK11_TP53 | 6 | 3292 | 0.001819 | 7 | 4005 | 0.001745 | 1 | 1 |
| KEAP1_PTPRD_TP53 | 32 | 3266 | 0.009703 | 14 | 3998 | 0.00349 | 0.000938 | 0.638491 |
| KEAP1_PTPRT | 16 | 3282 | 0.004851 | 1 | 4011 | 0.000249 | 2.83E-05 | 0.019277 |
| KEAP1_PTPRT_SMARCA4 | 5 | 3293 | 0.001516 | 1 | 4011 | 0.000249 | 0.097192 | 1 |
| KEAP1_PTPRT_STK11 | 7 | 3291 | 0.002122 | 1 | 4011 | 0.000249 | 0.026579 | 1 |
| KEAP1_PTPRT_TP53 | 11 | 3287 | 0.003335 | 0 | 4012 | 0 | 0.000156 | 0.106373 |
| KEAP1_RB1 | 22 | 3276 | 0.006671 | 15 | 3997 | 0.003739 | 0.097014 | 1 |
| KEAP1_RB1_SETD2 | 6 | 3292 | 0.001819 | 1 | 4011 | 0.000249 | 0.051102 | 1 |
| KEAP1_RB1_SETD2_TP53 | 5 | 3293 | 0.001516 | 1 | 4011 | 0.000249 | 0.097192 | 1 |
| KEAP1_RB1_STK11 | 5 | 3293 | 0.001516 | 1 | 4011 | 0.000249 | 0.097192 | 1 |
| KEAP1_RB1_TP53 | 17 | 3281 | 0.005155 | 13 | 3999 | 0.00324 | 0.269746 | 1 |
| KEAP1_RBM10 | 23 | 3275 | 0.006974 | 12 | 4000 | 0.002991 | 0.016608 | 1 |
| KEAP1_RBM10_SMARCA4 | 9 | 3289 | 0.002729 | 6 | 4006 | 0.001496 | 0.302316 | 1 |
| KEAP1_RBM10_SMARCA4_STK11 | 7 | 3291 | 0.002122 | 3 | 4009 | 0.000748 | 0.201315 | 1 |
| KEAP1_RBM10_STK11 | 12 | 3286 | 0.003639 | 6 | 4006 | 0.001496 | 0.09464 | 1 |
| KEAP1_RBM10_TP53 | 10 | 3288 | 0.003032 | 7 | 4005 | 0.001745 | 0.330321 | 1 |
| KEAP1_SETD2 | 28 | 3270 | 0.00849 | 28 | 3984 | 0.006979 | 0.50145 | 1 |
| KEAP1_SETD2_SMARCA4 | 6 | 3292 | 0.001819 | 7 | 4005 | 0.001745 | 1 | 1 |
| KEAP1_SETD2_STK11 | 9 | 3289 | 0.002729 | 14 | 3998 | 0.00349 | 0.676407 | 1 |
| KEAP1_SETD2_TP53 | 22 | 3276 | 0.006671 | 12 | 4000 | 0.002991 | 0.024488 | 1 |
| KEAP1_SMARCA4 | 57 | 3241 | 0.017283 | 50 | 3962 | 0.012463 | 0.096208 | 1 |
| KEAP1_SMARCA4_STK11 | 33 | 3265 | 0.010006 | 34 | 3978 | 0.008475 | 0.538211 | 1 |
| KEAP1_SMARCA4_STK11_TP53 | 11 | 3287 | 0.003335 | 10 | 4002 | 0.002493 | 0.518426 | 1 |
| KEAP1_SMARCA4_TP53 | 29 | 3269 | 0.008793 | 22 | 3990 | 0.005484 | 0.119515 | 1 |
| KEAP1_STK11 | 149 | 3149 | 0.045179 | 105 | 3907 | 0.026171 | 1.16E-05 | 0.007925 |
| KEAP1_STK11_TP53 | 59 | 3239 | 0.01789 | 39 | 3973 | 0.009721 | 0.002908 | 1 |
| KEAP1_TP53 | 170 | 3128 | 0.051546 | 136 | 3876 | 0.033898 | 0.000209 | 0.142382 |
| KMT2D | 172 | 3126 | 0.052153 | 247 | 3765 | 0.061565 | 0.085851 | 1 |
| KMT2D_KRAS | 44 | 3254 | 0.013341 | 57 | 3955 | 0.014207 | 0.763846 | 1 |
| KMT2D_KRAS_PIK3CA | 5 | 3293 | 0.001516 | 5 | 4007 | 0.001246 | 0.762057 | 1 |
| KMT2D_KRAS_PTPRD | 6 | 3292 | 0.001819 | 2 | 4010 | 0.000499 | 0.151704 | 1 |
| KMT2D_KRAS_SMARCA4 | 5 | 3293 | 0.001516 | 7 | 4005 | 0.001745 | 1 | 1 |
| KMT2D_KRAS_STK11 | 14 | 3284 | 0.004245 | 13 | 3999 | 0.00324 | 0.562461 | 1 |
| KMT2D_KRAS_STK11_TP53 | 6 | 3292 | 0.001819 | 1 | 4011 | 0.000249 | 0.051102 | 1 |
| KMT2D_KRAS_TP53 | 27 | 3271 | 0.008187 | 24 | 3988 | 0.005982 | 0.262814 | 1 |
| KMT2D_MET | 10 | 3288 | 0.003032 | 17 | 3995 | 0.004237 | 0.443295 | 1 |
| KMT2D_MET_TP53 | 8 | 3290 | 0.002426 | 13 | 3999 | 0.00324 | 0.661751 | 1 |
| KMT2D_NF1 | 24 | 3274 | 0.007277 | 35 | 3977 | 0.008724 | 0.51426 | 1 |
| KMT2D_NF1_PIK3CA | 5 | 3293 | 0.001516 | 5 | 4007 | 0.001246 | 0.762057 | 1 |
| KMT2D_NF1_PTPRD | 6 | 3292 | 0.001819 | 6 | 4006 | 0.001496 | 0.777757 | 1 |
| KMT2D_NF1_PTPRD_TP53 | 6 | 3292 | 0.001819 | 3 | 4009 | 0.000748 | 0.315191 | 1 |
| KMT2D_NF1_SETD2 | 7 | 3291 | 0.002122 | 5 | 4007 | 0.001246 | 0.395739 | 1 |
| KMT2D_NF1_SETD2_TP53 | 7 | 3291 | 0.002122 | 5 | 4007 | 0.001246 | 0.395739 | 1 |
| KMT2D_NF1_TP53 | 23 | 3275 | 0.006974 | 25 | 3987 | 0.006231 | 0.771492 | 1 |
| KMT2D_PIK3CA | 17 | 3281 | 0.005155 | 30 | 3982 | 0.007478 | 0.241239 | 1 |
| KMT2D_PIK3CA_PTPRD | 5 | 3293 | 0.001516 | 0 | 4012 | 0 | 0.018661 | 1 |
| KMT2D_PIK3CA_TP53 | 12 | 3286 | 0.003639 | 21 | 3991 | 0.005234 | 0.381647 | 1 |
| KMT2D_PTPRD | 21 | 3277 | 0.006367 | 19 | 3993 | 0.004736 | 0.426057 | 1 |
| KMT2D_PTPRD_SMARCA4 | 6 | 3292 | 0.001819 | 3 | 4009 | 0.000748 | 0.315191 | 1 |
| KMT2D_PTPRD_SMARCA4_TP53 | 5 | 3293 | 0.001516 | 3 | 4009 | 0.000748 | 0.480506 | 1 |
| KMT2D_PTPRD_STK11 | 5 | 3293 | 0.001516 | 6 | 4006 | 0.001496 | 1 | 1 |
| KMT2D_PTPRD_TP53 | 16 | 3282 | 0.004851 | 15 | 3997 | 0.003739 | 0.475654 | 1 |
| KMT2D_RB1 | 14 | 3284 | 0.004245 | 14 | 3998 | 0.00349 | 0.704437 | 1 |
| KMT2D_RB1_SMARCA4 | 5 | 3293 | 0.001516 | 0 | 4012 | 0 | 0.018661 | 1 |
| KMT2D_RB1_SMARCA4_TP53 | 5 | 3293 | 0.001516 | 0 | 4012 | 0 | 0.018661 | 1 |
| KMT2D_RB1_TP53 | 12 | 3286 | 0.003639 | 11 | 4001 | 0.002742 | 0.533855 | 1 |
| KMT2D_RBM10 | 8 | 3290 | 0.002426 | 12 | 4000 | 0.002991 | 0.822712 | 1 |
| KMT2D_RBM10_TP53 | 6 | 3292 | 0.001819 | 9 | 4003 | 0.002243 | 0.798223 | 1 |
| KMT2D_SETD2 | 21 | 3277 | 0.006367 | 29 | 3983 | 0.007228 | 0.672193 | 1 |
| KMT2D_SETD2_TP53 | 16 | 3282 | 0.004851 | 19 | 3993 | 0.004736 | 1 | 1 |
| KMT2D_SMARCA4 | 29 | 3269 | 0.008793 | 27 | 3985 | 0.00673 | 0.346403 | 1 |
| KMT2D_SMARCA4_TP53 | 21 | 3277 | 0.006367 | 18 | 3994 | 0.004487 | 0.333208 | 1 |
| KMT2D_STK11 | 25 | 3273 | 0.00758 | 39 | 3973 | 0.009721 | 0.37771 | 1 |
| KMT2D_STK11_TP53 | 16 | 3282 | 0.004851 | 17 | 3995 | 0.004237 | 0.728293 | 1 |
| KMT2D_TP53 | 120 | 3178 | 0.036386 | 159 | 3853 | 0.039631 | 0.500002 | 1 |
| KRAS | 867 | 2431 | 0.262887 | 1281 | 2731 | 0.319292 | 1.40E-07 | 9.51E-05 |
| KRAS_MET | 18 | 3280 | 0.005458 | 27 | 3985 | 0.00673 | 0.549335 | 1 |
| KRAS_MET_TP53 | 10 | 3288 | 0.003032 | 12 | 4000 | 0.002991 | 1 | 1 |
| KRAS_MGA | 18 | 3280 | 0.005458 | 25 | 3987 | 0.006231 | 0.759203 | 1 |
| KRAS_MGA_TP53 | 9 | 3289 | 0.002729 | 10 | 4002 | 0.002493 | 1 | 1 |
| KRAS_NF1 | 38 | 3260 | 0.011522 | 52 | 3960 | 0.012961 | 0.595955 | 1 |
| KRAS_NF1_PTPRD | 8 | 3290 | 0.002426 | 4 | 4008 | 0.000997 | 0.15444 | 1 |
| KRAS_NF1_PTPRD_TP53 | 8 | 3290 | 0.002426 | 2 | 4010 | 0.000499 | 0.050581 | 1 |
| KRAS_NF1_SMARCA4 | 6 | 3292 | 0.001819 | 6 | 4006 | 0.001496 | 0.777757 | 1 |
| KRAS_NF1_STK11 | 7 | 3291 | 0.002122 | 7 | 4005 | 0.001745 | 0.791309 | 1 |
| KRAS_NF1_TP53 | 23 | 3275 | 0.006974 | 23 | 3989 | 0.005733 | 0.553416 | 1 |
| KRAS_PIK3CA | 66 | 3232 | 0.020012 | 64 | 3948 | 0.015952 | 0.21312 | 1 |
| KRAS_PIK3CA_RB1 | 6 | 3292 | 0.001819 | 4 | 4008 | 0.000997 | 0.362025 | 1 |
| KRAS_PIK3CA_RB1_TP53 | 6 | 3292 | 0.001819 | 3 | 4009 | 0.000748 | 0.315191 | 1 |
| KRAS_PIK3CA_RBM10 | 5 | 3293 | 0.001516 | 3 | 4009 | 0.000748 | 0.480506 | 1 |
| KRAS_PIK3CA_STK11 | 8 | 3290 | 0.002426 | 9 | 4003 | 0.002243 | 1 | 1 |
| KRAS_PIK3CA_TP53 | 35 | 3263 | 0.010612 | 29 | 3983 | 0.007228 | 0.131021 | 1 |
| KRAS_PTPN11 | 12 | 3286 | 0.003639 | 10 | 4002 | 0.002493 | 0.397723 | 1 |
| KRAS_PTPN11_TP53 | 8 | 3290 | 0.002426 | 4 | 4008 | 0.000997 | 0.15444 | 1 |
| KRAS_PTPRD | 73 | 3225 | 0.022135 | 40 | 3972 | 0.00997 | 3.44E-05 | 0.023427 |
| KRAS_PTPRD_RBM10 | 7 | 3291 | 0.002122 | 2 | 4010 | 0.000499 | 0.088269 | 1 |
| KRAS_PTPRD_SETD2 | 7 | 3291 | 0.002122 | 2 | 4010 | 0.000499 | 0.088269 | 1 |
| KRAS_PTPRD_SMARCA4 | 10 | 3288 | 0.003032 | 6 | 4006 | 0.001496 | 0.209439 | 1 |
| KRAS_PTPRD_SMARCA4_TP53 | 7 | 3291 | 0.002122 | 1 | 4011 | 0.000249 | 0.026579 | 1 |
| KRAS_PTPRD_STK11 | 15 | 3283 | 0.004548 | 7 | 4005 | 0.001745 | 0.032787 | 1 |
| KRAS_PTPRD_TP53 | 38 | 3260 | 0.011522 | 17 | 3995 | 0.004237 | 0.000356 | 0.242415 |
| KRAS_PTPRT | 15 | 3283 | 0.004548 | 3 | 4009 | 0.000748 | 0.001336 | 0.909988 |
| KRAS_PTPRT_TP53 | 7 | 3291 | 0.002122 | 3 | 4009 | 0.000748 | 0.201315 | 1 |
| KRAS_RB1 | 33 | 3265 | 0.010006 | 39 | 3973 | 0.009721 | 0.905876 | 1 |
| KRAS_RB1_STK11 | 10 | 3288 | 0.003032 | 4 | 4008 | 0.000997 | 0.059853 | 1 |
| KRAS_RB1_TP53 | 23 | 3275 | 0.006974 | 23 | 3989 | 0.005733 | 0.553416 | 1 |
| KRAS_RBM10 | 49 | 3249 | 0.014857 | 35 | 3977 | 0.008724 | 0.015267 | 1 |
| KRAS_RBM10_SMARCA4 | 8 | 3290 | 0.002426 | 6 | 4006 | 0.001496 | 0.426098 | 1 |
| KRAS_RBM10_STK11 | 10 | 3288 | 0.003032 | 7 | 4005 | 0.001745 | 0.330321 | 1 |
| KRAS_RBM10_TP53 | 20 | 3278 | 0.006064 | 10 | 4002 | 0.002493 | 0.025714 | 1 |
| KRAS_SETD2 | 36 | 3262 | 0.010916 | 47 | 3965 | 0.011715 | 0.824634 | 1 |
| KRAS_SETD2_SMARCA4 | 5 | 3293 | 0.001516 | 9 | 4003 | 0.002243 | 0.595265 | 1 |
| KRAS_SETD2_STK11 | 8 | 3290 | 0.002426 | 9 | 4003 | 0.002243 | 1 | 1 |
| KRAS_SETD2_TP53 | 20 | 3278 | 0.006064 | 15 | 3997 | 0.003739 | 0.174142 | 1 |
| KRAS_SMARCA4 | 62 | 3236 | 0.018799 | 51 | 3961 | 0.012712 | 0.044967 | 1 |
| KRAS_SMARCA4_STK11 | 31 | 3267 | 0.0094 | 25 | 3987 | 0.006231 | 0.138351 | 1 |
| KRAS_SMARCA4_STK11_TP53 | 8 | 3290 | 0.002426 | 3 | 4009 | 0.000748 | 0.075396 | 1 |
| KRAS_SMARCA4_TP53 | 27 | 3271 | 0.008187 | 10 | 4002 | 0.002493 | 0.000744 | 0.50692 |
| KRAS_STK11 | 190 | 3108 | 0.057611 | 181 | 3831 | 0.045115 | 0.01598 | 1 |
| KRAS_STK11_TP53 | 56 | 3242 | 0.01698 | 28 | 3984 | 0.006979 | 9.05E-05 | 0.061659 |
| KRAS_TP53 | 381 | 2917 | 0.115525 | 401 | 3611 | 0.09995 | 0.033231 | 1 |
| MET | 126 | 3172 | 0.038205 | 141 | 3871 | 0.035145 | 0.491412 | 1 |
| MET_NF1 | 12 | 3286 | 0.003639 | 20 | 3992 | 0.004985 | 0.47729 | 1 |
| MET_NF1_TP53 | 8 | 3290 | 0.002426 | 15 | 3997 | 0.003739 | 0.402857 | 1 |
| MET_PIK3CA | 7 | 3291 | 0.002122 | 6 | 4006 | 0.001496 | 0.584537 | 1 |
| MET_PTPRD | 11 | 3287 | 0.003335 | 14 | 3998 | 0.00349 | 1 | 1 |
| MET_PTPRD_TP53 | 8 | 3290 | 0.002426 | 12 | 4000 | 0.002991 | 0.822712 | 1 |
| MET_RB1 | 11 | 3287 | 0.003335 | 17 | 3995 | 0.004237 | 0.573572 | 1 |
| MET_RB1_TP53 | 8 | 3290 | 0.002426 | 10 | 4002 | 0.002493 | 1 | 1 |
| MET_RBM10 | 8 | 3290 | 0.002426 | 6 | 4006 | 0.001496 | 0.426098 | 1 |
| MET_SETD2 | 6 | 3292 | 0.001819 | 11 | 4001 | 0.002742 | 0.472426 | 1 |
| MET_SMARCA4 | 13 | 3285 | 0.003942 | 9 | 4003 | 0.002243 | 0.20357 | 1 |
| MET_SMARCA4_TP53 | 7 | 3291 | 0.002122 | 7 | 4005 | 0.001745 | 0.791309 | 1 |
| MET_STK11 | 8 | 3290 | 0.002426 | 17 | 3995 | 0.004237 | 0.228698 | 1 |
| MET_STK11_TP53 | 5 | 3293 | 0.001516 | 10 | 4002 | 0.002493 | 0.441537 | 1 |
| MET_TP53 | 67 | 3231 | 0.020315 | 71 | 3941 | 0.017697 | 0.437487 | 1 |
| MGA | 49 | 3249 | 0.014857 | 57 | 3955 | 0.014207 | 0.844459 | 1 |
| MGA_NF1 | 7 | 3291 | 0.002122 | 8 | 4004 | 0.001994 | 1 | 1 |
| MGA_NF1_TP53 | 7 | 3291 | 0.002122 | 4 | 4008 | 0.000997 | 0.239779 | 1 |
| MGA_PIK3CA | 5 | 3293 | 0.001516 | 5 | 4007 | 0.001246 | 0.762057 | 1 |
| MGA_PTPRD | 6 | 3292 | 0.001819 | 1 | 4011 | 0.000249 | 0.051102 | 1 |
| MGA_PTPRD_TP53 | 5 | 3293 | 0.001516 | 1 | 4011 | 0.000249 | 0.097192 | 1 |
| MGA_PTPRT | 5 | 3293 | 0.001516 | 0 | 4012 | 0 | 0.018661 | 1 |
| MGA_RBM10 | 8 | 3290 | 0.002426 | 3 | 4009 | 0.000748 | 0.075396 | 1 |
| MGA_RBM10_TP53 | 6 | 3292 | 0.001819 | 3 | 4009 | 0.000748 | 0.315191 | 1 |
| MGA_SETD2 | 6 | 3292 | 0.001819 | 9 | 4003 | 0.002243 | 0.798223 | 1 |
| MGA_SMARCA4 | 7 | 3291 | 0.002122 | 4 | 4008 | 0.000997 | 0.239779 | 1 |
| MGA_SMARCA4_TP53 | 5 | 3293 | 0.001516 | 2 | 4010 | 0.000499 | 0.255537 | 1 |
| MGA_STK11 | 9 | 3289 | 0.002729 | 10 | 4002 | 0.002493 | 1 | 1 |
| MGA_STK11_TP53 | 7 | 3291 | 0.002122 | 4 | 4008 | 0.000997 | 0.239779 | 1 |
| MGA_TP53 | 35 | 3263 | 0.010612 | 28 | 3984 | 0.006979 | 0.099496 | 1 |
| NF1 | 205 | 3093 | 0.062159 | 201 | 3811 | 0.0501 | 0.027273 | 1 |
| NF1_PIK3CA | 17 | 3281 | 0.005155 | 18 | 3994 | 0.004487 | 0.734877 | 1 |
| NF1_PIK3CA_TP53 | 13 | 3285 | 0.003942 | 15 | 3997 | 0.003739 | 1 | 1 |
| NF1_PTPRD | 33 | 3265 | 0.010006 | 19 | 3993 | 0.004736 | 0.011098 | 1 |
| NF1_PTPRD_SMARCA4 | 8 | 3290 | 0.002426 | 3 | 4009 | 0.000748 | 0.075396 | 1 |
| NF1_PTPRD_SMARCA4_TP53 | 8 | 3290 | 0.002426 | 3 | 4009 | 0.000748 | 0.075396 | 1 |
| NF1_PTPRD_TP53 | 29 | 3269 | 0.008793 | 13 | 3999 | 0.00324 | 0.002672 | 1 |
| NF1_PTPRT | 10 | 3288 | 0.003032 | 0 | 4012 | 0 | 0.000347 | 0.236169 |
| NF1_PTPRT_TP53 | 9 | 3289 | 0.002729 | 0 | 4012 | 0 | 0.00077 | 0.524254 |
| NF1_RB1 | 20 | 3278 | 0.006064 | 11 | 4001 | 0.002742 | 0.044855 | 1 |
| NF1_RB1_TP53 | 18 | 3280 | 0.005458 | 8 | 4004 | 0.001994 | 0.016765 | 1 |
| NF1_RBM10 | 10 | 3288 | 0.003032 | 9 | 4003 | 0.002243 | 0.645555 | 1 |
| NF1_RBM10_TP53 | 7 | 3291 | 0.002122 | 6 | 4006 | 0.001496 | 0.584537 | 1 |
| NF1_SETD2 | 17 | 3281 | 0.005155 | 17 | 3995 | 0.004237 | 0.606819 | 1 |
| NF1_SETD2_TP53 | 15 | 3283 | 0.004548 | 11 | 4001 | 0.002742 | 0.23713 | 1 |
| NF1_SMARCA4 | 36 | 3262 | 0.010916 | 29 | 3983 | 0.007228 | 0.104012 | 1 |
| NF1_SMARCA4_STK11 | 8 | 3290 | 0.002426 | 7 | 4005 | 0.001745 | 0.607342 | 1 |
| NF1_SMARCA4_STK11_TP53 | 6 | 3292 | 0.001819 | 6 | 4006 | 0.001496 | 0.777757 | 1 |
| NF1_SMARCA4_TP53 | 30 | 3268 | 0.009096 | 23 | 3989 | 0.005733 | 0.097653 | 1 |
| NF1_STK11 | 29 | 3269 | 0.008793 | 32 | 3980 | 0.007976 | 0.700782 | 1 |
| NF1_STK11_TP53 | 19 | 3279 | 0.005761 | 18 | 3994 | 0.004487 | 0.508746 | 1 |
| NF1_TP53 | 155 | 3143 | 0.046998 | 128 | 3884 | 0.031904 | 0.000979 | 0.666641 |
| PIK3CA | 238 | 3060 | 0.072165 | 242 | 3770 | 0.060319 | 0.04619 | 1 |
| PIK3CA_PTPRD | 12 | 3286 | 0.003639 | 4 | 4008 | 0.000997 | 0.021618 | 1 |
| PIK3CA_PTPRD_TP53 | 11 | 3287 | 0.003335 | 2 | 4010 | 0.000499 | 0.004621 | 1 |
| PIK3CA_PTPRT | 7 | 3291 | 0.002122 | 0 | 4012 | 0 | 0.003792 | 1 |
| PIK3CA_PTPRT_TP53 | 5 | 3293 | 0.001516 | 0 | 4012 | 0 | 0.018661 | 1 |
| PIK3CA_RB1 | 17 | 3281 | 0.005155 | 16 | 3996 | 0.003988 | 0.486899 | 1 |
| PIK3CA_RB1_TP53 | 16 | 3282 | 0.004851 | 13 | 3999 | 0.00324 | 0.350151 | 1 |
| PIK3CA_RBM10 | 11 | 3287 | 0.003335 | 7 | 4005 | 0.001745 | 0.235379 | 1 |
| PIK3CA_SETD2 | 14 | 3284 | 0.004245 | 13 | 3999 | 0.00324 | 0.562461 | 1 |
| PIK3CA_SETD2_TP53 | 10 | 3288 | 0.003032 | 8 | 4004 | 0.001994 | 0.478233 | 1 |
| PIK3CA_SMARCA4 | 10 | 3288 | 0.003032 | 11 | 4001 | 0.002742 | 0.829722 | 1 |
| PIK3CA_SMARCA4_TP53 | 6 | 3292 | 0.001819 | 6 | 4006 | 0.001496 | 0.777757 | 1 |
| PIK3CA_STK11 | 20 | 3278 | 0.006064 | 18 | 3994 | 0.004487 | 0.414462 | 1 |
| PIK3CA_STK11_TP53 | 13 | 3285 | 0.003942 | 10 | 4002 | 0.002493 | 0.29892 | 1 |
| PIK3CA_TP53 | 141 | 3157 | 0.042753 | 139 | 3873 | 0.034646 | 0.075832 | 1 |
| PTPN11 | 31 | 3267 | 0.0094 | 32 | 3980 | 0.007976 | 0.527205 | 1 |
| PTPN11_RB1 | 5 | 3293 | 0.001516 | 4 | 4008 | 0.000997 | 0.739872 | 1 |
| PTPN11_RB1_TP53 | 5 | 3293 | 0.001516 | 3 | 4009 | 0.000748 | 0.480506 | 1 |
| PTPN11_TP53 | 21 | 3277 | 0.006367 | 16 | 3996 | 0.003988 | 0.185164 | 1 |
| PTPRD | 169 | 3129 | 0.051243 | 117 | 3895 | 0.029163 | 1.48E-06 | 0.001009 |
| PTPRD_PTPRT | 17 | 3281 | 0.005155 | 0 | 4012 | 0 | 1.30E-06 | 0.000885 |
| PTPRD_PTPRT_RBM10 | 5 | 3293 | 0.001516 | 0 | 4012 | 0 | 0.018661 | 1 |
| PTPRD_PTPRT_SMARCA4 | 7 | 3291 | 0.002122 | 0 | 4012 | 0 | 0.003792 | 1 |
| PTPRD_PTPRT_SMARCA4_TP53 | 5 | 3293 | 0.001516 | 0 | 4012 | 0 | 0.018661 | 1 |
| PTPRD_PTPRT_TP53 | 14 | 3284 | 0.004245 | 0 | 4012 | 0 | 1.43E-05 | 0.00971 |
| PTPRD_RB1 | 12 | 3286 | 0.003639 | 9 | 4003 | 0.002243 | 0.280904 | 1 |
| PTPRD_RB1_SETD2 | 5 | 3293 | 0.001516 | 1 | 4011 | 0.000249 | 0.097192 | 1 |
| PTPRD_RB1_SETD2_TP53 | 5 | 3293 | 0.001516 | 1 | 4011 | 0.000249 | 0.097192 | 1 |
| PTPRD_RB1_TP53 | 12 | 3286 | 0.003639 | 7 | 4005 | 0.001745 | 0.164482 | 1 |
| PTPRD_RBM10 | 14 | 3284 | 0.004245 | 3 | 4009 | 0.000748 | 0.002455 | 1 |
| PTPRD_RBM10_TP53 | 9 | 3289 | 0.002729 | 1 | 4011 | 0.000249 | 0.007044 | 1 |
| PTPRD_SETD2 | 20 | 3278 | 0.006064 | 9 | 4003 | 0.002243 | 0.013728 | 1 |
| PTPRD_SETD2_TP53 | 15 | 3283 | 0.004548 | 5 | 4007 | 0.001246 | 0.011309 | 1 |
| PTPRD_SMARCA4 | 34 | 3264 | 0.010309 | 15 | 3997 | 0.003739 | 0.000767 | 0.522002 |
| PTPRD_SMARCA4_STK11 | 8 | 3290 | 0.002426 | 5 | 4007 | 0.001246 | 0.272151 | 1 |
| PTPRD_SMARCA4_TP53 | 26 | 3272 | 0.007884 | 9 | 4003 | 0.002243 | 0.000542 | 0.369427 |
| PTPRD_STK11 | 27 | 3271 | 0.008187 | 29 | 3983 | 0.007228 | 0.686812 | 1 |
| PTPRD_STK11_TP53 | 10 | 3288 | 0.003032 | 17 | 3995 | 0.004237 | 0.443295 | 1 |
| PTPRD_TP53 | 116 | 3182 | 0.035173 | 77 | 3935 | 0.019192 | 2.63E-05 | 0.017916 |
| PTPRT | 61 | 3237 | 0.018496 | 7 | 4005 | 0.001745 | 1.26E-14 | 8.61E-12 |
| PTPRT_RBM10 | 6 | 3292 | 0.001819 | 0 | 4012 | 0 | 0.008412 | 1 |
| PTPRT_SMARCA4 | 15 | 3283 | 0.004548 | 1 | 4011 | 0.000249 | 5.94E-05 | 0.040426 |
| PTPRT_SMARCA4_STK11 | 5 | 3293 | 0.001516 | 1 | 4011 | 0.000249 | 0.097192 | 1 |
| PTPRT_SMARCA4_TP53 | 11 | 3287 | 0.003335 | 0 | 4012 | 0 | 0.000156 | 0.106373 |
| PTPRT_STK11 | 13 | 3285 | 0.003942 | 2 | 4010 | 0.000499 | 0.001251 | 0.852126 |
| PTPRT_STK11_TP53 | 7 | 3291 | 0.002122 | 1 | 4011 | 0.000249 | 0.026579 | 1 |
| PTPRT_TP53 | 42 | 3256 | 0.012735 | 5 | 4007 | 0.001246 | 2.47E-10 | 1.68E-07 |
| RB1 | 179 | 3119 | 0.054275 | 152 | 3860 | 0.037886 | 0.000843 | 0.573938 |
| RB1_SETD2 | 13 | 3285 | 0.003942 | 4 | 4008 | 0.000997 | 0.01273 | 1 |
| RB1_SETD2_TP53 | 12 | 3286 | 0.003639 | 4 | 4008 | 0.000997 | 0.021618 | 1 |
| RB1_SMARCA4 | 16 | 3282 | 0.004851 | 10 | 4002 | 0.002493 | 0.113889 | 1 |
| RB1_SMARCA4_TP53 | 13 | 3285 | 0.003942 | 8 | 4004 | 0.001994 | 0.130069 | 1 |
| RB1_STK11 | 17 | 3281 | 0.005155 | 14 | 3998 | 0.00349 | 0.284119 | 1 |
| RB1_STK11_TP53 | 11 | 3287 | 0.003335 | 8 | 4004 | 0.001994 | 0.356301 | 1 |
| RB1_TP53 | 146 | 3152 | 0.044269 | 104 | 3908 | 0.025922 | 2.38E-05 | 0.016216 |
| RBM10 | 107 | 3191 | 0.032444 | 80 | 3932 | 0.01994 | 0.000793 | 0.53985 |
| RBM10_SETD2 | 10 | 3288 | 0.003032 | 9 | 4003 | 0.002243 | 0.645555 | 1 |
| RBM10_SETD2_TP53 | 6 | 3292 | 0.001819 | 6 | 4006 | 0.001496 | 0.777757 | 1 |
| RBM10_SMARCA4 | 17 | 3281 | 0.005155 | 11 | 4001 | 0.002742 | 0.126871 | 1 |
| RBM10_SMARCA4_STK11 | 8 | 3290 | 0.002426 | 5 | 4007 | 0.001246 | 0.272151 | 1 |
| RBM10_SMARCA4_TP53 | 7 | 3291 | 0.002122 | 4 | 4008 | 0.000997 | 0.239779 | 1 |
| RBM10_STK11 | 18 | 3280 | 0.005458 | 16 | 3996 | 0.003988 | 0.390645 | 1 |
| RBM10_STK11_TP53 | 5 | 3293 | 0.001516 | 6 | 4006 | 0.001496 | 1 | 1 |
| RBM10_TP53 | 51 | 3247 | 0.015464 | 39 | 3973 | 0.009721 | 0.03254 | 1 |
| SETD2 | 154 | 3144 | 0.046695 | 150 | 3862 | 0.037388 | 0.051941 | 1 |
| SETD2_SMARCA4 | 19 | 3279 | 0.005761 | 21 | 3991 | 0.005234 | 0.753451 | 1 |
| SETD2_SMARCA4_STK11 | 5 | 3293 | 0.001516 | 8 | 4004 | 0.001994 | 0.782726 | 1 |
| SETD2_SMARCA4_TP53 | 15 | 3283 | 0.004548 | 12 | 4000 | 0.002991 | 0.333589 | 1 |
| SETD2_STK11 | 17 | 3281 | 0.005155 | 28 | 3984 | 0.006979 | 0.368646 | 1 |
| SETD2_STK11_TP53 | 7 | 3291 | 0.002122 | 13 | 3999 | 0.00324 | 0.500739 | 1 |
| SETD2_TP53 | 95 | 3203 | 0.028805 | 63 | 3949 | 0.015703 | 0.000137 | 0.093067 |
| SMARCA4 | 208 | 3090 | 0.063069 | 154 | 3858 | 0.038385 | 1.68E-06 | 0.001143 |
| SMARCA4_STK11 | 63 | 3235 | 0.019102 | 55 | 3957 | 0.013709 | 0.07627 | 1 |
| SMARCA4_STK11_TP53 | 29 | 3269 | 0.008793 | 21 | 3991 | 0.005234 | 0.086073 | 1 |
| SMARCA4_TP53 | 140 | 3158 | 0.04245 | 79 | 3933 | 0.019691 | 1.66E-08 | 1.13E-05 |
| STK11 | 347 | 2951 | 0.105215 | 389 | 3623 | 0.096959 | 0.257361 | 1 |
| STK11_TP53 | 166 | 3132 | 0.050334 | 136 | 3876 | 0.033898 | 0.000482 | 0.32835 |
| TP53 | 1738 | 1560 | 0.526986 | 1665 | 2347 | 0.415005 | 1.29E-21 | 8.77E-19 |

**Supplementary** **Table S5.**

Differences in the mutational frequency between genes (or gene modules) in early and metastatic Skin Cancer Non-Melanoma (SKCNM).

| Gene_Module | #mut_mSkin_Cancer._Non.Melanoma | #wild_mSkin_Cancer._Non.Melanoma | mRatio | #mut_earlySkin_Cancer._Non.Melanoma | #wild_earlySkin_Cancer._Non.Melanoma | early_Ratio | pvalue | qvalue |
| --- | --- | --- | --- | --- | --- | --- | --- | --- |
| PTPRD | 9 | 105 | 0.078947 | 15 | 136 | 0.099338 | 0.667762 | 1 |
| PTPRT | 6 | 108 | 0.052632 | 0 | 151 | 0 | 0.00587 | 0.01174 |

**Supplementary** **Table S6.**

Differences in the mutational frequency between genes (or gene modules) in early and metastatic Skin Cancer Non-Melanoma (SKCM).

| Gene_Module | #mut_mMelanoma | #wild_mMelanoma | mRatio | #mut_earlyMelanoma | #wild_earlyMelanoma | early_Ratio | pvalue | qvalue |
| --- | --- | --- | --- | --- | --- | --- | --- | --- |
| ARID2 | 76 | 1072 | 0.066202 | 37 | 632 | 0.055306 | 0.366948 | 1 |
| ARID2_BRAF | 32 | 1116 | 0.027875 | 17 | 652 | 0.025411 | 0.880835 | 1 |
| ARID2_BRAF_CDKN2A | 7 | 1141 | 0.006098 | 3 | 666 | 0.004484 | 0.753841 | 1 |
| ARID2_BRAF_MECOM | 6 | 1142 | 0.005226 | 6 | 663 | 0.008969 | 0.376039 | 1 |
| ARID2_BRAF_MECOM_NRAS | 5 | 1143 | 0.004355 | 1 | 668 | 0.001495 | 0.423413 | 1 |
| ARID2_BRAF_NF1 | 12 | 1136 | 0.010453 | 10 | 659 | 0.014948 | 0.505205 | 1 |
| ARID2_BRAF_NF1_NRAS | 6 | 1142 | 0.005226 | 3 | 666 | 0.004484 | 1 | 1 |
| ARID2_BRAF_NF1_PTPRD | 5 | 1143 | 0.004355 | 2 | 667 | 0.00299 | 1 | 1 |
| ARID2_BRAF_NRAS | 9 | 1139 | 0.00784 | 3 | 666 | 0.004484 | 0.552287 | 1 |
| ARID2_BRAF_PTPN11 | 6 | 1142 | 0.005226 | 2 | 667 | 0.00299 | 0.717967 | 1 |
| ARID2_BRAF_PTPRD | 5 | 1143 | 0.004355 | 3 | 666 | 0.004484 | 1 | 1 |
| ARID2_BRAF_TP53 | 8 | 1140 | 0.006969 | 6 | 663 | 0.008969 | 0.781853 | 1 |
| ARID2_CDKN2A | 16 | 1132 | 0.013937 | 5 | 664 | 0.007474 | 0.259995 | 1 |
| ARID2_CDKN2A_NF1 | 9 | 1139 | 0.00784 | 3 | 666 | 0.004484 | 0.552287 | 1 |
| ARID2_CDKN2A_NF1_PTPRD | 5 | 1143 | 0.004355 | 0 | 669 | 0 | 0.164911 | 1 |
| ARID2_CDKN2A_NF1_TP53 | 6 | 1142 | 0.005226 | 1 | 668 | 0.001495 | 0.434109 | 1 |
| ARID2_CDKN2A_NRAS | 7 | 1141 | 0.006098 | 1 | 668 | 0.001495 | 0.271076 | 1 |
| ARID2_CDKN2A_PTPRD | 5 | 1143 | 0.004355 | 1 | 668 | 0.001495 | 0.423413 | 1 |
| ARID2_CDKN2A_TP53 | 6 | 1142 | 0.005226 | 2 | 667 | 0.00299 | 0.717967 | 1 |
| ARID2_CTNNB1 | 5 | 1143 | 0.004355 | 1 | 668 | 0.001495 | 0.423413 | 1 |
| ARID2_KIT | 5 | 1143 | 0.004355 | 2 | 667 | 0.00299 | 1 | 1 |
| ARID2_MAP2K1 | 7 | 1141 | 0.006098 | 2 | 667 | 0.00299 | 0.499075 | 1 |
| ARID2_MECOM | 11 | 1137 | 0.009582 | 15 | 654 | 0.022422 | 0.038452 | 1 |
| ARID2_MECOM_NF1 | 9 | 1139 | 0.00784 | 8 | 661 | 0.011958 | 0.450232 | 1 |
| ARID2_MECOM_NF1_TP53 | 5 | 1143 | 0.004355 | 4 | 665 | 0.005979 | 0.7327 | 1 |
| ARID2_MECOM_NRAS | 6 | 1142 | 0.005226 | 5 | 664 | 0.007474 | 0.546124 | 1 |
| ARID2_MECOM_TP53 | 6 | 1142 | 0.005226 | 7 | 662 | 0.010463 | 0.249954 | 1 |
| ARID2_NF1 | 34 | 1114 | 0.029617 | 20 | 649 | 0.029895 | 1 | 1 |
| ARID2_NF1_NRAS | 8 | 1140 | 0.006969 | 4 | 665 | 0.005979 | 1 | 1 |
| ARID2_NF1_PTPN11 | 7 | 1141 | 0.006098 | 3 | 666 | 0.004484 | 0.753841 | 1 |
| ARID2_NF1_PTPRD | 9 | 1139 | 0.00784 | 2 | 667 | 0.00299 | 0.346827 | 1 |
| ARID2_NF1_TP53 | 13 | 1135 | 0.011324 | 8 | 661 | 0.011958 | 1 | 1 |
| ARID2_NRAS | 28 | 1120 | 0.02439 | 11 | 658 | 0.016442 | 0.315073 | 1 |
| ARID2_NRAS_PTPRD | 5 | 1143 | 0.004355 | 1 | 668 | 0.001495 | 0.423413 | 1 |
| ARID2_NRAS_TP53 | 9 | 1139 | 0.00784 | 0 | 669 | 0 | 0.030874 | 1 |
| ARID2_PTPN11 | 9 | 1139 | 0.00784 | 3 | 666 | 0.004484 | 0.552287 | 1 |
| ARID2_PTPRD | 10 | 1138 | 0.008711 | 4 | 665 | 0.005979 | 0.591262 | 1 |
| ARID2_RB1 | 5 | 1143 | 0.004355 | 2 | 667 | 0.00299 | 1 | 1 |
| ARID2_TP53 | 22 | 1126 | 0.019164 | 13 | 656 | 0.019432 | 1 | 1 |
| BRAF | 486 | 662 | 0.423345 | 231 | 438 | 0.345291 | 0.001027 | 0.165271 |
| BRAF_CDKN2A | 67 | 1081 | 0.058362 | 31 | 638 | 0.046338 | 0.284144 | 1 |
| BRAF_CDKN2A_CTNNB1 | 7 | 1141 | 0.006098 | 4 | 665 | 0.005979 | 1 | 1 |
| BRAF_CDKN2A_NF1 | 12 | 1136 | 0.010453 | 3 | 666 | 0.004484 | 0.281469 | 1 |
| BRAF_CDKN2A_NF1_TP53 | 8 | 1140 | 0.006969 | 1 | 668 | 0.001495 | 0.167133 | 1 |
| BRAF_CDKN2A_PTEN | 10 | 1138 | 0.008711 | 4 | 665 | 0.005979 | 0.591262 | 1 |
| BRAF_CDKN2A_PTPRD | 9 | 1139 | 0.00784 | 1 | 668 | 0.001495 | 0.102938 | 1 |
| BRAF_CDKN2A_PTPRT | 5 | 1143 | 0.004355 | 0 | 669 | 0 | 0.164911 | 1 |
| BRAF_CDKN2A_TP53 | 19 | 1129 | 0.016551 | 11 | 658 | 0.016442 | 1 | 1 |
| BRAF_CTNNB1 | 26 | 1122 | 0.022648 | 13 | 656 | 0.019432 | 0.738463 | 1 |
| BRAF_CTNNB1_TP53 | 5 | 1143 | 0.004355 | 2 | 667 | 0.00299 | 1 | 1 |
| BRAF_IDH1 | 12 | 1136 | 0.010453 | 8 | 661 | 0.011958 | 0.817263 | 1 |
| BRAF_KIT | 16 | 1132 | 0.013937 | 10 | 659 | 0.014948 | 0.840674 | 1 |
| BRAF_KIT_TP53 | 6 | 1142 | 0.005226 | 2 | 667 | 0.00299 | 0.717967 | 1 |
| BRAF_MAP2K1 | 12 | 1136 | 0.010453 | 5 | 664 | 0.007474 | 0.620056 | 1 |
| BRAF_MECOM | 24 | 1124 | 0.020906 | 13 | 656 | 0.019432 | 1 | 1 |
| BRAF_MECOM_NF1 | 14 | 1134 | 0.012195 | 7 | 662 | 0.010463 | 0.823288 | 1 |
| BRAF_MECOM_NF1_NRAS | 5 | 1143 | 0.004355 | 3 | 666 | 0.004484 | 1 | 1 |
| BRAF_MECOM_NF1_TP53 | 6 | 1142 | 0.005226 | 3 | 666 | 0.004484 | 1 | 1 |
| BRAF_MECOM_NRAS | 9 | 1139 | 0.00784 | 3 | 666 | 0.004484 | 0.552287 | 1 |
| BRAF_MECOM_TP53 | 10 | 1138 | 0.008711 | 7 | 662 | 0.010463 | 0.801649 | 1 |
| BRAF_NF1 | 43 | 1105 | 0.037456 | 28 | 641 | 0.041854 | 0.706718 | 1 |
| BRAF_NF1_NRAS | 13 | 1135 | 0.011324 | 6 | 663 | 0.008969 | 0.812003 | 1 |
| BRAF_NF1_PTEN | 5 | 1143 | 0.004355 | 7 | 662 | 0.010463 | 0.139199 | 1 |
| BRAF_NF1_PTPN11 | 6 | 1142 | 0.005226 | 2 | 667 | 0.00299 | 0.717967 | 1 |
| BRAF_NF1_PTPRD | 7 | 1141 | 0.006098 | 3 | 666 | 0.004484 | 0.753841 | 1 |
| BRAF_NF1_RB1 | 5 | 1143 | 0.004355 | 2 | 667 | 0.00299 | 1 | 1 |
| BRAF_NF1_TP53 | 14 | 1134 | 0.012195 | 7 | 662 | 0.010463 | 0.823288 | 1 |
| BRAF_NRAS | 32 | 1116 | 0.027875 | 11 | 658 | 0.016442 | 0.149684 | 1 |
| BRAF_NRAS_TP53 | 5 | 1143 | 0.004355 | 5 | 664 | 0.007474 | 0.512646 | 1 |
| BRAF_PTEN | 48 | 1100 | 0.041812 | 28 | 641 | 0.041854 | 1 | 1 |
| BRAF_PTPN11 | 10 | 1138 | 0.008711 | 3 | 666 | 0.004484 | 0.394537 | 1 |
| BRAF_PTPRD | 21 | 1127 | 0.018293 | 8 | 661 | 0.011958 | 0.337733 | 1 |
| BRAF_PTPRD_PTPRT | 5 | 1143 | 0.004355 | 0 | 669 | 0 | 0.164911 | 1 |
| BRAF_PTPRD_TP53 | 8 | 1140 | 0.006969 | 4 | 665 | 0.005979 | 1 | 1 |
| BRAF_PTPRT | 16 | 1132 | 0.013937 | 0 | 669 | 0 | 0.000967 | 0.155621 |
| BRAF_RB1 | 16 | 1132 | 0.013937 | 5 | 664 | 0.007474 | 0.259995 | 1 |
| BRAF_RB1_TP53 | 7 | 1141 | 0.006098 | 3 | 666 | 0.004484 | 0.753841 | 1 |
| BRAF_TP53 | 93 | 1055 | 0.08101 | 38 | 631 | 0.056801 | 0.059883 | 1 |
| CDKN2A | 131 | 1017 | 0.114111 | 73 | 596 | 0.109118 | 0.758691 | 1 |
| CDKN2A_CTNNB1 | 11 | 1137 | 0.009582 | 8 | 661 | 0.011958 | 0.638554 | 1 |
| CDKN2A_KIT | 5 | 1143 | 0.004355 | 3 | 666 | 0.004484 | 1 | 1 |
| CDKN2A_MAP2K1 | 8 | 1140 | 0.006969 | 3 | 666 | 0.004484 | 0.755535 | 1 |
| CDKN2A_MECOM | 13 | 1135 | 0.011324 | 12 | 657 | 0.017937 | 0.296532 | 1 |
| CDKN2A_MECOM_NF1 | 6 | 1142 | 0.005226 | 5 | 664 | 0.007474 | 0.546124 | 1 |
| CDKN2A_MECOM_NF1_TP53 | 6 | 1142 | 0.005226 | 4 | 665 | 0.005979 | 1 | 1 |
| CDKN2A_MECOM_NRAS | 5 | 1143 | 0.004355 | 8 | 661 | 0.011958 | 0.08271 | 1 |
| CDKN2A_MECOM_TP53 | 11 | 1137 | 0.009582 | 7 | 662 | 0.010463 | 0.812062 | 1 |
| CDKN2A_NF1 | 29 | 1119 | 0.025261 | 10 | 659 | 0.014948 | 0.17931 | 1 |
| CDKN2A_NF1_NRAS | 7 | 1141 | 0.006098 | 2 | 667 | 0.00299 | 0.499075 | 1 |
| CDKN2A_NF1_PTPRD | 6 | 1142 | 0.005226 | 1 | 668 | 0.001495 | 0.434109 | 1 |
| CDKN2A_NF1_TP53 | 15 | 1133 | 0.013066 | 8 | 661 | 0.011958 | 1 | 1 |
| CDKN2A_NRAS | 34 | 1114 | 0.029617 | 23 | 646 | 0.03438 | 0.579083 | 1 |
| CDKN2A_NRAS_TP53 | 8 | 1140 | 0.006969 | 9 | 660 | 0.013453 | 0.206724 | 1 |
| CDKN2A_PTEN | 14 | 1134 | 0.012195 | 6 | 663 | 0.008969 | 0.644218 | 1 |
| CDKN2A_PTPRD | 13 | 1135 | 0.011324 | 3 | 666 | 0.004484 | 0.192479 | 1 |
| CDKN2A_PTPRD_TP53 | 7 | 1141 | 0.006098 | 2 | 667 | 0.00299 | 0.499075 | 1 |
| CDKN2A_PTPRT | 5 | 1143 | 0.004355 | 0 | 669 | 0 | 0.164911 | 1 |
| CDKN2A_TP53 | 42 | 1106 | 0.036585 | 30 | 639 | 0.044843 | 0.385383 | 1 |
| CTNNB1 | 48 | 1100 | 0.041812 | 25 | 644 | 0.037369 | 0.710892 | 1 |
| CTNNB1_IDH1 | 6 | 1142 | 0.005226 | 3 | 666 | 0.004484 | 1 | 1 |
| CTNNB1_NF1 | 7 | 1141 | 0.006098 | 3 | 666 | 0.004484 | 0.753841 | 1 |
| CTNNB1_NRAS | 14 | 1134 | 0.012195 | 5 | 664 | 0.007474 | 0.474366 | 1 |
| CTNNB1_PTPRD | 5 | 1143 | 0.004355 | 0 | 669 | 0 | 0.164911 | 1 |
| CTNNB1_TP53 | 7 | 1141 | 0.006098 | 3 | 666 | 0.004484 | 0.753841 | 1 |
| IDH1 | 34 | 1114 | 0.029617 | 16 | 653 | 0.023916 | 0.553013 | 1 |
| IDH1_NRAS | 15 | 1133 | 0.013066 | 8 | 661 | 0.011958 | 1 | 1 |
| IDH1_TP53 | 7 | 1141 | 0.006098 | 3 | 666 | 0.004484 | 0.753841 | 1 |
| KIT | 46 | 1102 | 0.04007 | 55 | 614 | 0.082212 | 0.000267 | 0.043062 |
| KIT_MECOM | 7 | 1141 | 0.006098 | 2 | 667 | 0.00299 | 0.499075 | 1 |
| KIT_MECOM_NF1 | 6 | 1142 | 0.005226 | 2 | 667 | 0.00299 | 0.717967 | 1 |
| KIT_NF1 | 13 | 1135 | 0.011324 | 9 | 660 | 0.013453 | 0.664185 | 1 |
| KIT_NF1_TP53 | 6 | 1142 | 0.005226 | 2 | 667 | 0.00299 | 0.717967 | 1 |
| KIT_NRAS | 6 | 1142 | 0.005226 | 5 | 664 | 0.007474 | 0.546124 | 1 |
| KIT_TP53 | 12 | 1136 | 0.010453 | 12 | 657 | 0.017937 | 0.202809 | 1 |
| MAP2K1 | 27 | 1121 | 0.023519 | 15 | 654 | 0.022422 | 1 | 1 |
| MAP2K1_NF1 | 9 | 1139 | 0.00784 | 4 | 665 | 0.005979 | 0.778081 | 1 |
| MAP2K1_NRAS | 6 | 1142 | 0.005226 | 6 | 663 | 0.008969 | 0.376039 | 1 |
| MAP2K1_TP53 | 5 | 1143 | 0.004355 | 2 | 667 | 0.00299 | 1 | 1 |
| MECOM | 52 | 1096 | 0.045296 | 41 | 628 | 0.061286 | 0.151317 | 1 |
| MECOM_NF1 | 26 | 1122 | 0.022648 | 18 | 651 | 0.026906 | 0.635477 | 1 |
| MECOM_NF1_NRAS | 8 | 1140 | 0.006969 | 5 | 664 | 0.007474 | 1 | 1 |
| MECOM_NF1_NRAS_TP53 | 5 | 1143 | 0.004355 | 2 | 667 | 0.00299 | 1 | 1 |
| MECOM_NF1_PTPRD | 5 | 1143 | 0.004355 | 5 | 664 | 0.007474 | 0.512646 | 1 |
| MECOM_NF1_TP53 | 14 | 1134 | 0.012195 | 9 | 660 | 0.013453 | 0.830005 | 1 |
| MECOM_NRAS | 21 | 1127 | 0.018293 | 18 | 651 | 0.026906 | 0.241649 | 1 |
| MECOM_NRAS_TP53 | 8 | 1140 | 0.006969 | 6 | 663 | 0.008969 | 0.781853 | 1 |
| MECOM_PTPRD | 11 | 1137 | 0.009582 | 6 | 663 | 0.008969 | 1 | 1 |
| MECOM_PTPRD_TP53 | 6 | 1142 | 0.005226 | 4 | 665 | 0.005979 | 1 | 1 |
| MECOM_RB1 | 6 | 1142 | 0.005226 | 3 | 666 | 0.004484 | 1 | 1 |
| MECOM_TP53 | 24 | 1124 | 0.020906 | 19 | 650 | 0.028401 | 0.338325 | 1 |
| NF1 | 117 | 1031 | 0.101916 | 66 | 603 | 0.098655 | 0.871725 | 1 |
| NF1_NRAS | 24 | 1124 | 0.020906 | 12 | 657 | 0.017937 | 0.729556 | 1 |
| NF1_NRAS_PTPRD | 5 | 1143 | 0.004355 | 1 | 668 | 0.001495 | 0.423413 | 1 |
| NF1_NRAS_TP53 | 7 | 1141 | 0.006098 | 3 | 666 | 0.004484 | 0.753841 | 1 |
| NF1_PTEN | 12 | 1136 | 0.010453 | 8 | 661 | 0.011958 | 0.817263 | 1 |
| NF1_PTPN11 | 10 | 1138 | 0.008711 | 9 | 660 | 0.013453 | 0.347303 | 1 |
| NF1_PTPN11_TP53 | 6 | 1142 | 0.005226 | 3 | 666 | 0.004484 | 1 | 1 |
| NF1_PTPRD | 15 | 1133 | 0.013066 | 7 | 662 | 0.010463 | 0.824511 | 1 |
| NF1_PTPRD_TP53 | 7 | 1141 | 0.006098 | 5 | 664 | 0.007474 | 0.768326 | 1 |
| NF1_RB1 | 12 | 1136 | 0.010453 | 5 | 664 | 0.007474 | 0.620056 | 1 |
| NF1_RB1_TP53 | 6 | 1142 | 0.005226 | 3 | 666 | 0.004484 | 1 | 1 |
| NF1_TP53 | 36 | 1112 | 0.031359 | 24 | 645 | 0.035874 | 0.589533 | 1 |
| NRAS | 269 | 879 | 0.234321 | 149 | 520 | 0.22272 | 0.603104 | 1 |
| NRAS_PTEN | 11 | 1137 | 0.009582 | 5 | 664 | 0.007474 | 0.796881 | 1 |
| NRAS_PTPN11 | 5 | 1143 | 0.004355 | 2 | 667 | 0.00299 | 1 | 1 |
| NRAS_PTPRD | 10 | 1138 | 0.008711 | 7 | 662 | 0.010463 | 0.801649 | 1 |
| NRAS_RB1 | 12 | 1136 | 0.010453 | 2 | 667 | 0.00299 | 0.097598 | 1 |
| NRAS_RB1_TP53 | 8 | 1140 | 0.006969 | 0 | 669 | 0 | 0.030055 | 1 |
| NRAS_TP53 | 59 | 1089 | 0.051394 | 24 | 645 | 0.035874 | 0.131669 | 1 |
| PTEN | 70 | 1078 | 0.060976 | 43 | 626 | 0.064275 | 0.763795 | 1 |
| PTEN_TP53 | 10 | 1138 | 0.008711 | 11 | 658 | 0.016442 | 0.171572 | 1 |
| PTPN11 | 20 | 1128 | 0.017422 | 17 | 652 | 0.025411 | 0.301187 | 1 |
| PTPN11_TP53 | 8 | 1140 | 0.006969 | 8 | 661 | 0.011958 | 0.302823 | 1 |
| PTPRD | 37 | 1111 | 0.03223 | 18 | 651 | 0.026906 | 0.572405 | 1 |
| PTPRD_PTPRT | 5 | 1143 | 0.004355 | 0 | 669 | 0 | 0.164911 | 1 |
| PTPRD_RB1 | 6 | 1142 | 0.005226 | 5 | 664 | 0.007474 | 0.546124 | 1 |
| PTPRD_TP53 | 16 | 1132 | 0.013937 | 8 | 661 | 0.011958 | 0.832974 | 1 |
| PTPRT | 16 | 1132 | 0.013937 | 0 | 669 | 0 | 0.000967 | 0.155621 |
| RB1 | 38 | 1110 | 0.033101 | 13 | 656 | 0.019432 | 0.105059 | 1 |
| RB1_TP53 | 18 | 1130 | 0.015679 | 4 | 665 | 0.005979 | 0.076979 | 1 |
| TP53 | 217 | 931 | 0.189024 | 120 | 549 | 0.179372 | 0.617388 | 1 |
